# Kinetic Resolution of Epimeric Proteins Enables Stereoselective Chemical Mutagenesis

**DOI:** 10.1021/jacs.4c07103

**Published:** 2024-07-31

**Authors:** Guljannat Ablat, Neev Lawton, Ruqaiya Alam, Bethany A. Haynes, Sabrina Hossain, Thomas Hicks, Sasha L. Evans, James A. Jarvis, Timothy J. Nott, Rivka L. Isaacson, Manuel M. Müller

**Affiliations:** † Department of Chemistry, King’s College London, Britannia House, 7 Trinity Street, London SE1 1DB, U.K.; ‡ Randall Centre for Cell and Molecular Biophysics and Centre for Biomolecular Spectroscopy, King’s College London, New Hunts House, London SE1 1UL, U.K.

## Abstract

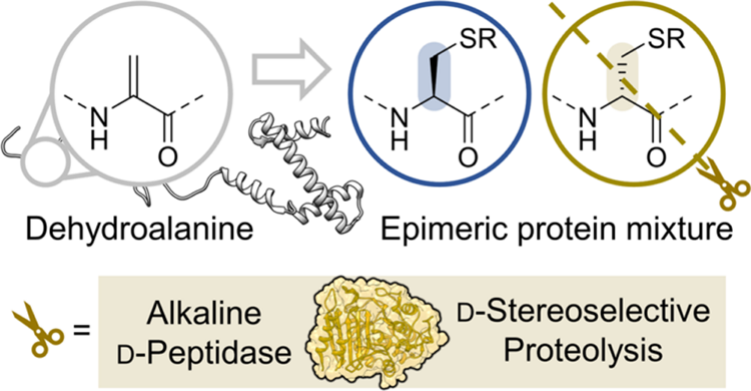

Chemical mutagenesis via dehydroalanine (Dha) is a powerful method to tailor protein structure and function, allowing the site-specific installation of post-translational modifications and non-natural functional groups. Despite the impressive versatility of this method, applications have been limited, as products are formed as epimeric mixtures, whereby the modified amino acid is present as both the desired l-configuration and a roughly equal amount of the undesired d-isomer. Here, we describe a simple remedy for this issue: removal of the d-isomer via proteolysis using a d-stereoselective peptidase, alkaline d-peptidase (AD-P). We demonstrate that AD-P can selectively cleave the d-isomer of epimeric residues within histone H3, GFP, Ddx4, and SGTA, allowing the installation of non-natural amino acids with stereochemical control. Given the breadth of modifications that can be introduced via Dha and the simplicity of our method, we believe that stereoselective chemoenzymatic mutagenesis will find broad utility in protein engineering and chemical biology applications.

## Introduction

Post-translational modifications (PTMs) dramatically enhance the functional diversity of proteins. They introduce new chemical groups that are not directly accessible within the standard proteinogenic amino acid alphabets and can act as reversible switches to control protein activity.^1^ Chemists have recognized the potential of emulating this process and have since developed creative ways to selectively modify proteins,^2,3^ enabling installation of new-to-nature functionalities, as well as mimicking natural PTMs.^4^ These efforts have yielded powerful tools to engineer proteins^5^ and to dissect the role of endogenous PTMs in biology and disease.^6,7^

**Figure 1:**
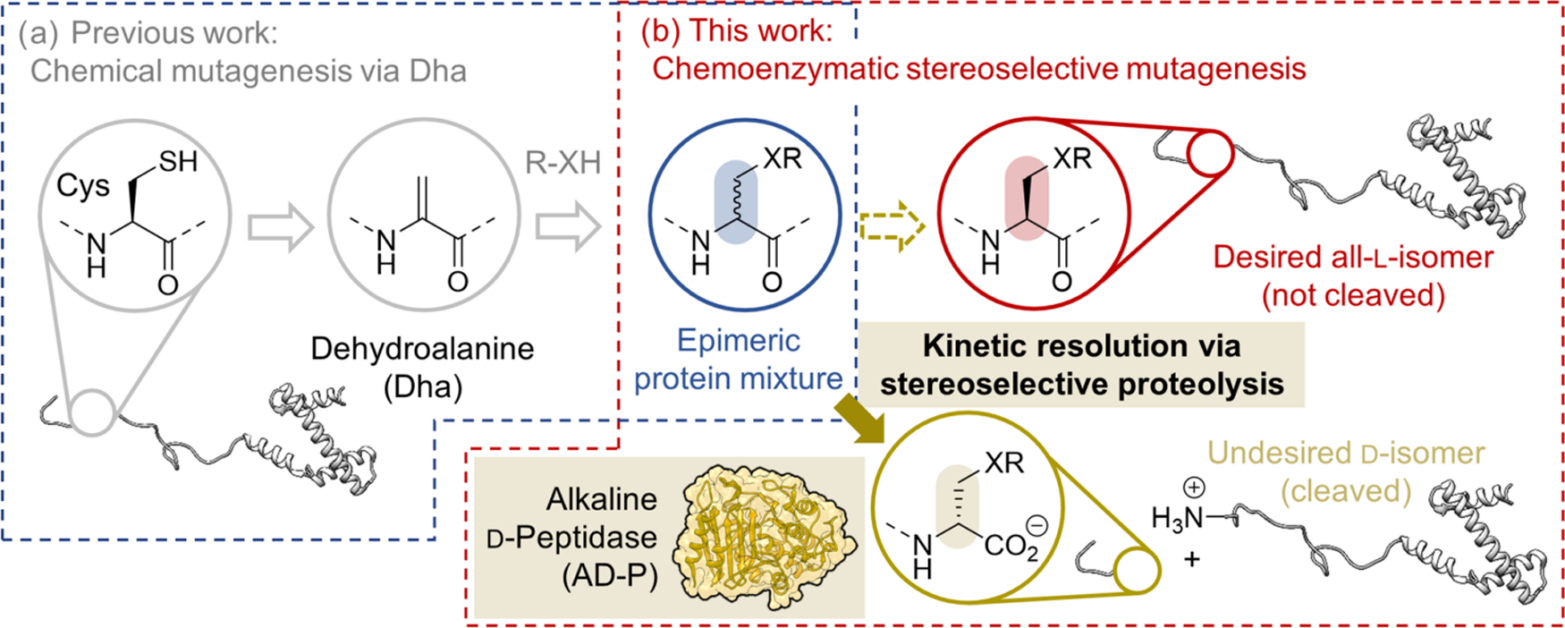
Outline of the stereoselective chemoenzymatic protein mutagenesis concept. (a) Previous work enables the selective conversion of Cys into a plethora of side chains in the form of epimeric mixtures. (b) Kinetic resolution via stereoselective proteolysis removes the undesirable d-isomer (beige), leaving the desired all-l-isomer intact (red).

Dehydroalanine (Dha) has emerged as a particularly versatile synthetic intermediate (Figure 1a).^8,9^ It can be readily installed onto proteins in a site-selective manner,^10^ typically using Cys^11^ or non-natural amino acids as reactive precursors.^12,13^ The unique reactivity of Dha has facilitated the biocompatible installation of various desired functional groups. For example, its α,β-unsaturated carbonyl moiety can undergo conjugate additions to connect Cβ to N-, Se-, or S-nucleophiles,^8^ as well as allow metal-catalyzed Cβ–C,^14,15^ Cβ–B,^16^ and Cβ–Si^17^ bond formation. Dha is also an efficient radical acceptor which has been exploited for the chemoselective formation of C–C bonds on proteins.^9,13,18^

Chemical mutagenesis via Dha has enabled a range of applications. For example, designer chromatin-bearing biochemically relevant PTMs and their analogues have been generated by functionalizing Dha-containing histones; such investigations have furthered the understanding of how these PTMs are interpreted and edited.^9,13,19,20^ Similarly, mechanistic studies on the site-specific incorporation of phosphorylated Ser and Thr analogues into protein kinases have highlighted the chemical and geometrical features of phosphorylation and kinase activation in regulatory loops.^21,22^ The same strategy has also been applied to probe the effect of PTMs in modulating amyloid-β peptide aggregation, which plays a role in Alzheimer’s disease.^23^ Non-natural amino acids that can be installed via Dha are also beneficial for protein engineering efforts; incorporation of nonstandard functional groups into active sites^24^ and binding pockets have been exploited to probe the role of hydrogen bonding in catalysis,^25^ to improve substrate recognition in enzymes^26^ and antibodies,^27^ and to generate de novo binding proteins,^16^ among others.

Despite its utility for protein engineering, chemical mutagenesis via Dha has a major drawback in that this method lacks stereocontrol. Instead, the process typically generates epimeric product mixtures, whereby the modified amino acid is present as both the l- and d-configuration at a roughly 1:1 ratio (Figure 1a).^8^ Epimerization at the mutation site is a significant limitation as relative contributions of each stereoisomer cannot easily be discerned, which makes quantitative biophysical measurements of PTM function challenging, as well as hampering Dha’s application toward, e.g., synthesizing pharmaceutical bioconjugates.^28^ We therefore set out to develop a strategy to remove the undesirable d-isomer.

Epimeric mixtures of proteins are challenging to separate. Although there are examples of serendipitous differential refolding/crystallization,^26^ a general method for protein diastereoisomer separation is required. Given the exquisite native stereoselectivity of many enzymes, we envisaged a chemoenzymatic editing approach. We therefore sought proteases that can stereoselectively cleave d-amino acids in the context of l-proteins. In this context, alkaline d-peptidase (AD-P) from *Bacillus cereus*(29) was of particular interest for developing this strategy due to its relatively large, open, active site,^30^ as well as its concomitant ability to process large substrates (evidenced by its suitability in protein ligation endeavors).^31^ Here, we describe the application of AD-P for the kinetic resolution of epimeric protein mixtures, a process that equips chemical mutagenesis via Dha with highly coveted stereocontrol.

## Results and Discussion

**Figure 2:**
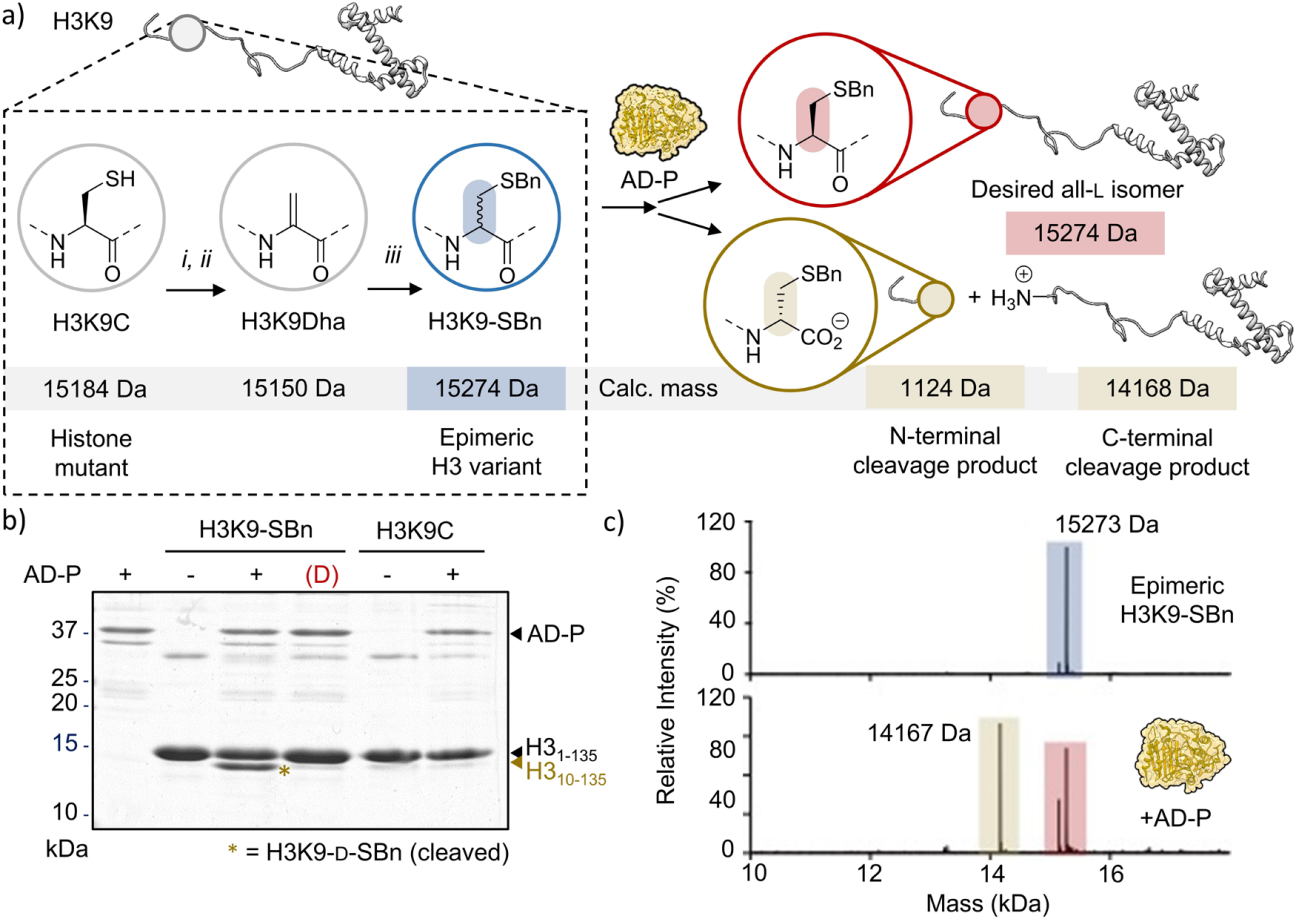
Stereoselective chemoenzymatic protein mutagenesis via kinetic resolution. (a) Schematic representation of the chemical conversion of histone H3K9C to H3K9-SBn. Reagents: (i) DTT; (ii) DBHDA; (iii) Benzyl mercaptan. (b) SDS-PAGE analysis of H3K9-SBn cleavage by AD-P. The C-terminal cleavage product (H3_10–135_) is marked with a brown asterisk. The red (D) denotes a denatured AD-P sample. (c) HRMS analysis of epimeric H3K9-SBn (top, blue) and products of the kinetic resolution reaction (bottom, cleaved: beige; uncleaved: red). For the epimeric mixture: calculated mass = 15 274 Da, observed mass = 15 273 Da. For the C-terminal cleavage product (d-isomer): calculated mass = 14 168 Da; observed mass = 14 167 Da.

We first set out to establish the feasibility of enzymatically resolving protein diastereoisomers. We chose histone H3 as a model system because histones are frequently manipulated with chemical mutagenesis techniques^9,18,20,32^ due to the importance of histone PTMs in gene regulation.^33^ Moreover, mutations at the sites of histone PTMs contribute to the development of several cancers^34^ and synthetic analogues are useful tools for establishing the mechanisms by which these mutations perturb epigenetic processes.^35,36^ We therefore produced a mutant of H3 with a unique Cys substituted for Lys9 (H3K9C) because of the significance of PTMs at this site for epigenetic regulation,^37,38^ and converted the engineered Cys residue to Dha with 2,5-dibromohexanediamide (DBHDA), resulting in H3K9Dha. Due to the reported selectivity of AD-P for d-Phe residues,^30^ we chose to functionalize H3K9Dha with benzyl mercaptan, resulting in an epimeric mixture of H3 diastereoisomers at the benzylated K9C residue in both the l- and the d-configuration (H3K9-SBn, Figures 2a and S2). H3K9-SBn (84 μM) was then subjected to cleavage with AD-P (7 μM). The latter was purified from *Escherichia coli* (Figure S3a,b) and confirmed to be proteolytically active, selectively cleaving C-terminal to a d-Leu residue in a model peptide (Figure S3c–e). Analysis of the AD-P-dependent H3 cleavage reaction by sodium dodecyl-sulfate polyacrylamide gel electrophoresis (SDS-PAGE) revealed the appearance of a new band at ∼14 kDa, located just below H3, consistent with site-specific cleavage at position 9 (Figure 2b, lane 3). As expected, control reactions in the absence of AD-P, with heat-denatured AD-P, or using unmodified H3K9C did not yield discernible cleavage events (Figures 2b, lanes 2,4–6 and S2d). Furthermore, HRMS analysis confirmed that the new species had a mass of 14 167 Da (Figures 2c and S2c), consistent with the removal of 9 amino acids from the N-terminus of H3. The site-specificity and approximately 1:1 ratio between the cleaved and uncleaved proteins, as inferred from both SDS-PAGE and MS analyses, are indicative of successful stereoselective targeting of the d-isomer. Using 3.7 μM AD-P, the half-time of the cleavage reaction of epimeric H3K9-SBn is approximately 6 min at 30 °C (Figure S4). AD-P was also active at 4 °C in the presence of up to 0.1 M l-Arg or 5% DMSO (Figures S5, S6 and Table S1). Addition of 0.1 M GdmCl, 1% DMF, or 0.17 M l-Arg diminished the activity of AD-P, as did heating the reactions to 40 °C. In the presence of ≥0.5 M GdmCl or 5% DMF, only trace amounts of AD-P activity were observed.

**Figure 3:**
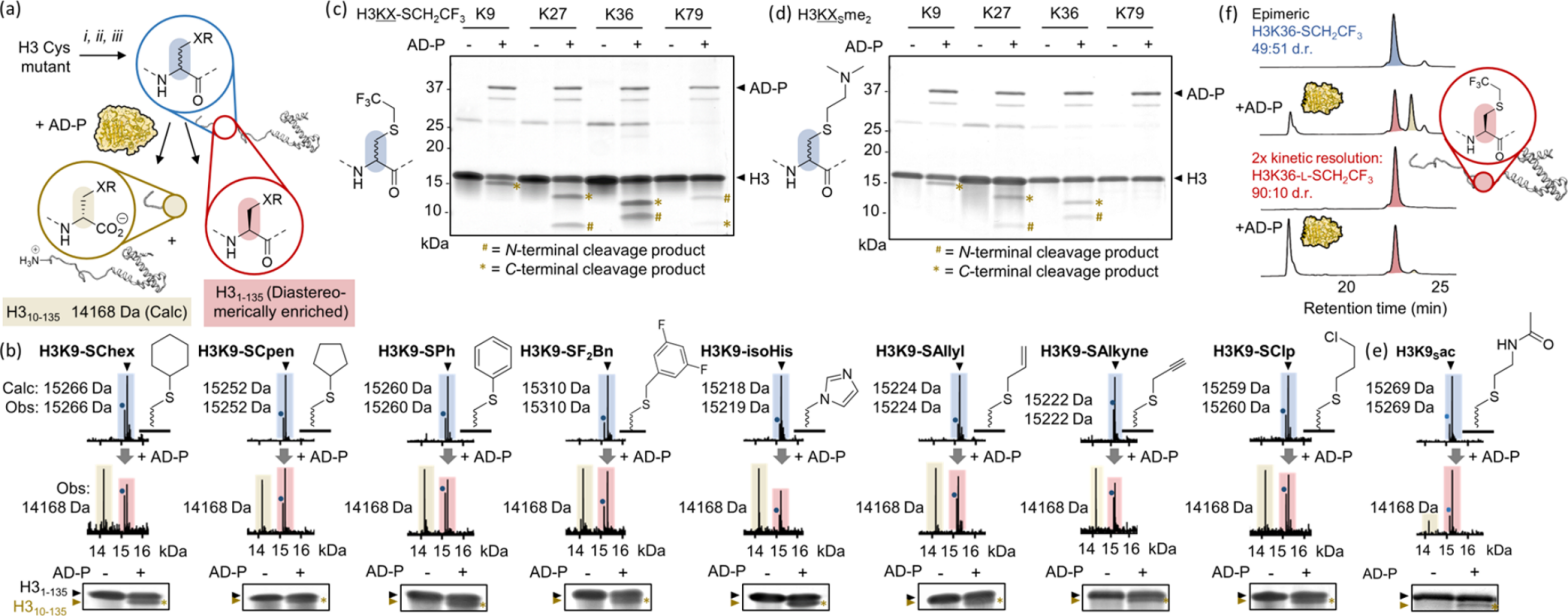
Substrate scope of stereoselective chemoenzymatic mutagenesis on histone H3. (a) Schematic representation of the synthesis and kinetic resolution of epimeric histones from Cys-containing H3 mutants. Reagents: (i) DTT; (ii) DBDHA; (iii) RSH or imidazole. (b) AD-P promiscuously cleaves hydrophobic d-amino acids within the H3 tail. HRMS (top) and SDS-PAGE analysis (bottom) of epimeric mixtures (blue) and the C-terminal cleavage products upon AD-P cleavage (H3_10–135_, beige MS peak; brown asterisk on gel). Blue circles in the MS denote residual H3K9Dha. (c, d) Selective cleavage of H3 variants with an artificial d-amino acid featuring a trifluoroethyl group (-SCH_2_CF_3_, c) or a dimethylysine analog (K_S_me_2_, d) at K9, K27, K36, and K79 by AD-P. N- and C-terminal cleavage products are indicated by brown hashes and asterisks, respectively. (e) Partial cleavage of acetyllysine analog H3K9_S_ac. (f) 2-step kinetic resolution to generate diastereomerically enriched H3K36-SCH_2_CF_3_.

We next explored the substrate scope of AD-P. We screened a range of hydrophobic side chains, including various geometries and electronic properties with potential utility in molecular recognition^27,35^ (cyclohexyl thiol, cyclopentyl thiol, thiophenol, difluorothiobenzene), mechanistic probes (isohistidine),^25^ and reactive handles (allyl thiol,^39^ propargyl thiol, chloropropyl thiol^40^). All epimeric proteins were cleaved by AD-P in approximately 50% yield as judged by mass spectrometry, highlighting AD-P’s versatility in cleaving hydrophobic d-amino acids (Figures 3a,b and S7–S10).

Furthermore, we installed isopropyl thiol (Figures S12–S14) and trifluoroethanethiol (Figures 3c and S15–S17) modifications onto several sites within histone H3 (K9, K27, K36 and K79). Such modifications can act as inhibitors of histone methyltransferases and therefore represent useful probe molecules.^35,36,42^ Moreover, trifluoromethyl groups provide sensitive handles for protein observation with ^19^F-NMR.^43^ AD-P successfully cleaved all sites within the disordered H3 tail to approximately 50%, as anticipated with a 1:1 epimeric mixture (Figures 3c and S12–S17). Cleavages at K79 were only partially successful, presumably due to the residue being encapsulated within the structured region of H3, and therefore less accessible to AD-P under the conditions of the cleavage. Despite its preference for hydrophobic residues, AD-P could also cleave H3 variants featuring a d-dimethyllysine analog at positions 9, 27, and 36, albeit at slower rates (Figures 3d, S17, and S18). A d-acetyllysine analog was a poor substrate (Figures 3e and S11), indicating that there are limits to the size and polarity of side chains that can be accommodated in the AD-P active site.

We scaled up the kinetic resolution reaction to 2.1 mg of epimeric H3K36-SCH_2_CF_3_ (49:51 d.r. based on HPLC analysis of the cleavage reaction), allowing a 0.7 mg isolation of diastereomerically enriched H3K36-l-SCH_2_CF_3_ with 77:23 d.r. upon AD-P cleavage and purification (Figures 3 and S19). Presumably, the limited solubility of individual histones under the reaction conditions precluded quantitative cleavage of the undesirable d-isomer; however, this was remedied by an additional round of cleavage and purification, leading to an improved d.r. of 90:10 (Figures 3f and S19). Such results demonstrated the feasibility for using chemoenzymatic mutagenesis toward generating histones bearing unnatural side chains in high stereochemical purity.

**Figure 4:**
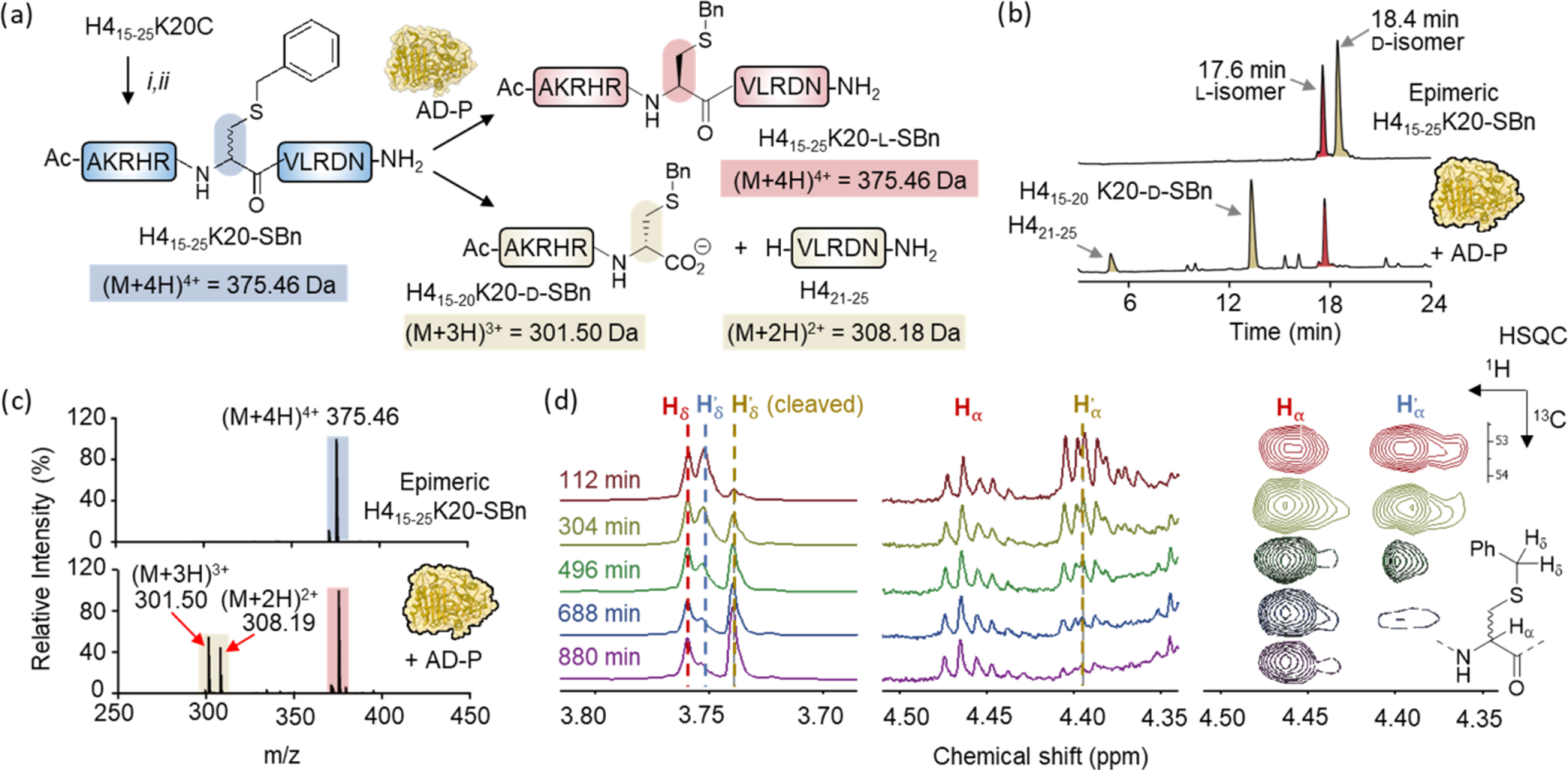
Mechanistic investigation of the kinetic resolution of an epimeric peptide. (a) Schematic representation of the synthesis and kinetic resolution of H4_15–25_K20-SBn from the histone peptide H4_15–25_ K20C. Reagents: DBHDA; (ii) Benzyl mercaptan. (b) RP-HPLC analysis confirming selective cleavage of the d-containing diastereoisomer (beige) by AD-P. (c) HRMS analysis of H4_15–25_ K20-SBn cleavage by AD-P. For epimeric H4_15–25_K20-SBn (blue) and its all-l-isoform (red), calculated mass = 1497.81 Da, observed mass = 1497.82 Da; for N-terminal cleaved product H4_15–20_K20-SBn (beige), calculated mass = 901.47, observed mass = 901.47; for C-terminal cleaved product H4_21–25_ (beige), calculated mass = 614.35, observed mass = 614.36. (d) NMR analysis of H4_15–25_K20-SBn kinetic resolution by AD-P. 800 MHz ^1^H NMR spectra encompassing H_δ_ (left) and H_α_ (middle) peaks as well as ^1^H–^13^C HSQC peaks encompassing H_α_ (right) of the benzyl cysteine residue are shown. The 2D Data (right) ranges from 4.51 to 4.34 ppm in the ^1^H dimension and from 54 to 52 ppm in the ^13^C dimension.

To investigate the stereoselectivity of AD-P cleavage, we used a peptide derived from the N-terminal tail of histone H4 due to the synthetic and analytical tractability of short peptides as compared to full-length proteins. We synthesized an 11-mer peptide encompassing residues 15–25 of human H4 (H4_15–25_K20C). As before, Cys20 was converted to Dha, and subsequently functionalized with benzyl mercaptan, yielding H4_15–25_K20-SBn as an epimeric mixture (Figures 4a and S20). The diastereoisomers of H4_15–25_K20-SBn elute from reverse phase HPLC as independent peaks, allowing direct monitoring of the kinetic resolution step (Figure 4b). A spike-in of the all-l-isomer H4_15–25_K20-l-SBn, synthesized via reaction of H4_15–25_K20C with BnBr (Figure S20g,h), allowed assignment of the peak at 17.6 min as H4_15–25_K20-l-SBn (Figure S21a,b). Accordingly, the 18.4 min peak can be assigned to the d-epimer at residue 20 (H4_15–25_K20-d-SBn). Upon incubation with 20.4 μM AD-P for 12 h, complete digestion of H4_15–25_K20-d-SBn is observed (Figure 4b,c). Concomitantly, both products expected from cleavage C-terminal to the d-SBn residue were observed by HPLC and confirmed by mass spectrometry (Figure 4b,c; H4_15–20_K20-d-SBn, at 13.3 min RT, (M + 3H)^3+^ observed = 301.50 Da, calculated = 301.50 Da; H4_21–25_ at 4.9 min RT, (M + 2H)^2+^ observed = 308.19 Da, calculated = 308.18 Da). A time course experiment over the first 3 h of the cleavage reaction revealed a gradual decrease in the level of H4-d-SBn, while that of H4-l-SBn remained intact (Figures 4 and S21c,d). These observations reinforce the expected stereoselectivity of AD-P and the application of this enzyme for kinetic resolutions.

Further insight into the cleavage selectivity was obtained by NMR spectroscopy. Given that the diastereotopic H_α_ (4.40 and 4.45 ppm) and H_δ_ atoms (3.75 and 3.76 ppm) of the modified -SBn residue exhibit subtly distinct chemical shifts at 800 MHz, we monitored the kinetic resolution by 1D- and 2D-NMR. The cleavage reaction showed clear diastereoselectivity, with cleavage causing the disappearance of the peaks at 4.40 ppm (H’_α_) and 3.75 ppm (H’_δ_), as well as the formation of a new peak at 3.74 ppm assigned to H_δ_ of the newly formed C-terminal residue (Figures 4d and S22). By contrast, the corresponding peaks of the l-isomer (4.46 ppm for H_α_ and 3.76 ppm for H_δ_) are retained throughout the kinetic resolution experiment. Collectively, these experiments demonstrate that AD-P cleavage is stereoselective and, due to the absence of other d-amino acids in recombinant proteins, also site-specific. AD-P thus provides an elegant means to kinetically resolve epimeric peptides and proteins.

**Figure 5:**
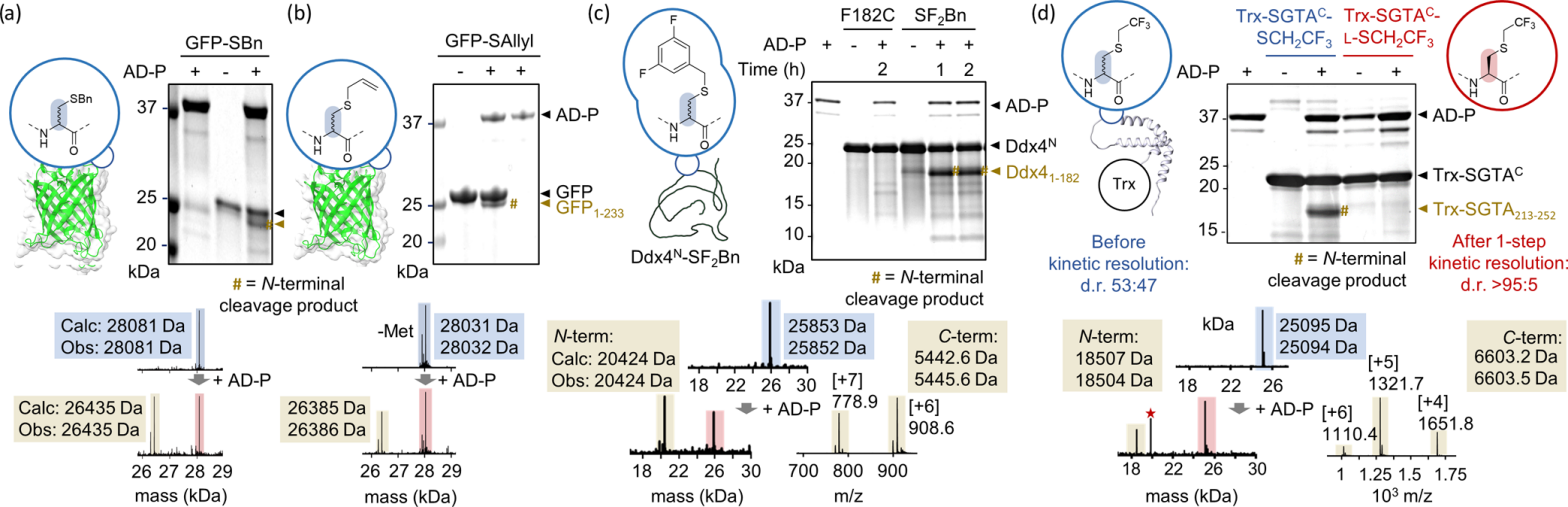
Kinetic resolution of epimeric proteins with AD-P. (a) GFP-SBn, (b) GFP-SAllyl, (c) Ddx4^N^-SF_2_Bn, and (d) Trx-SGTA^C^-SCH_2_CF_3_. In panel (d), cleavage of epimeric Trx-SGTA^C^-SCH_2_CF_3_ (left, blue) and recleavage of semipurified Trx-SGTA^C^-l-SCH_2_CF_3_ (right, red) are shown in the SDS-PAGE analysis. For all variants, SDS-PAGE (top) and MS analyses (bottom) are shown. The N-terminal cleavage products (GFP_1–233_, Ddx4_1–182_, or Trx-SGTA_213–252_) are marked with a brown hash in the gels. For Ddx4 and SGTA cleavages, both cleavage fragments can be detected by MS (deconvoluted and raw spectra are shown for the N- and C-terminal fragments, respectively). The red star indicates a peak corresponding to the (M + 2H)^2+^ species of AD-P (calculated *m*/*z* = 19 857; observed m/z = 19 856).

Next, we explored the applicability of AD-P to the kinetic resolution of diverse protein substrates. As model proteins, we chose green fluorescent protein (GFP), the substrate recognition domain of the quality control protein SGTA,^44,45^ and the intrinsically disordered region of Ddx4–a paradigm for protein liquid–liquid phase separation.^46^ All proteins were equipped with a single solvent-accessible Cys residue (GFP-M233C; Ddx4-F182C; SGTA-S252C) which was subsequently converted to Dha (Figures S23, S26, and S27). Gratifyingly, AD-P efficiently cleaved GFP functionalized with benzyl (GFP-SBn) and allyl mercaptan (GFP-SAllyl) moieties (Figures 5a,b, S24, and S25a). A variant of GFP containing β-thioglucose at position 233 (GFP_S_Glc) was not cleaved by AD-P, further indicating that large, polar modifications are not accommodated in AD-P’s hydrophobic binding pocket (Figure S25b–d). A Ddx4 variant with a difluorobenzyl group (Ddx4^N^-SF_2_Bn), which could have further applications as a probe of cation-π interactions in Ddx4 phase separation,^46^ was readily cleaved at the epimeric position by AD-P (Figures 5c and S26). In the case of Ddx4, minor levels of background cleavage of the unmodified parent protein were observed, possibly via miscleavage in Gly-rich regions, as Gly can adopt conformations that are otherwise restricted to d-amino acids. Finally, we generated a trifluoroethylated variant of SGTA (Trx-SGTA^C^-SCH_2_CF_3_), which also has further utility in probing substrate recognition via ^19^F-NMR. This construct was selectively and efficiently cleaved by AD-P within 1h (Figures 5d, S27, and S28). Analysis of partially purified Trx-SGTA^C^-l-SCH_2_CF_3_ by recleavage with AD-P revealed that for this protein, a single kinetic resolution step resulted in a d.r. of >95:5, as judged by SDS-PAGE (Figure 5d), demonstrating the efficiency and versatility of our approach.

## Conclusions

Chemical modification provides a powerful avenue to engineer the properties of proteins for basic research and therapeutic applications.^47^ Cys residues are ideal reactive handles for site-specific modifications due to their unique reactivity. A chemical challenge, especially in the context of PTM research, is the requirement for minimal perturbation of linkages between the protein and new functional groups. Creative solutions for this problem are Cys alkylation, frequently used to install methyllysine analogues,^48^ and recent desulfurative^49−52^ strategies to incorporate useful functional groups such as spin labels, PTMs, and their analogues.

Chemical mutagenesis via Dha is particularly versatile due to the diversity and yields of the products generated.^8^ However, with a few notable exceptions relying on stereoselective protein refolding,^26^ or modification with chiral Ni-complexes at the peptide level,^53^ this method is restricted due to a loss of stereochemical integrity at the modification site. Here, we provide an elegant solution to this problem, enabling us to generate stereoselectively modified proteins via a kinetic resolution step catalyzed by alkaline d-peptidase. This enzyme selectively proteolyzes the undesired d-isomer in solvent-exposed positions of histone H3, H4 peptides, GFP, Ddx4, and SGTA. AD-P exhibits a preference for a broad range of hydrophobic residues at the P1 site, including (hetero)aromatic, aliphatic, chlorinated, and fluorinated d-amino acids, and is able to cleave (to a lesser extent) the d-isomer of a dimethyllysine analog. The enzyme is remarkably promiscuous at the P2 (Gly, Arg, and hydrophobic amino acids) and P1’ sites (charged, polar, and small amino acids). This promiscuity supports the applicability of AD-P for the kinetic resolution of diverse protein substrates. We expect that further optimization of reaction conditions, as well as targeted engineering efforts of AD-P activity and specificity, will allow expansion of this technology to enable the stereoselective installation of a multitude of PTMs and their analogues into diverse protein targets. Indeed, the discovery of d-peptidases with inherent specificities for various sequences^54^ and residues (including d-Asp^55^ and d-Arg^56^) bodes well for the expansion of a powerful toolkit toward the stereoselective chemoenzymatic mutagenesis of proteins.

## Supporting Information Available

Supporting Tables and Figures; experimental procedures; characterization data (PDF)
