# Supplementary material for: Kinetic Resolution of Epimeric Proteins Enables Stereoselective Chemical Mutagenesis
Source: J Am Chem Soc. 2024 Jul 31;146(32):22622–8. doi: 10.1021/jacs.4c07103 (PMC11328163; doi:10.1021/jacs.4c07103)
Supplement: Supplementary file 1 — ja4c07103_si_001.pdf [file ja4c07103_si_001.pdf]

Supporting Information for:

Kinetic Resolution of Epimeric Proteins enables Stereoselective Chemical Mutagenesis

Guljannat Ablat[1], Neev Lawton[1], Ruqaiya Alam[1], Bethany A. Haynes[1], Sabrina Hossain[1], Thomas Hicks[1], Sasha L. Evans[1], James A. Jarvis[2], Timothy J. Nott[1], Rivka L. Isaacson[1] and Manuel M. Müller[1]*

[1]*Department of Chemistry, King's College London, Britannia House, 7 Trinity Street, London, SE1 1DB, UK*

[2]*Randall Centre for Cell and Molecular Biophysics and Centre for Biomolecular Spectroscopy, King's College London, New Hunts House, SE1 1UL, UK*

*Correspondence to M.M.M. (manuel.muller@kcl.ac.uk)

Contents

Kinetic Resolution of Epimeric Proteins enables Stereoselective Chemical Mutagenesis _____ 1

Contents _____ 2

Supporting Tables _____ 6

Table S1: Overview of conditions for the cleavage of peptides and proteins containing D-amino acid by Alkaline D-Peptidase (AD-P). _____ 6

Table S2: Primers used in this study. _____ 7

Supporting Figures _____ 8

Fig. S1: Production of H3 K9C. _____ 8

Fig. S2: Installation of benzyl modification on H3K9C and subsequent cleavage by AD-P. _____ 10

Fig. S3: Purification and activity test of alkaline D-peptidase (AD-P). _____ 12

Fig. S4: Cleavage of H3K9-SBn by AD-P. _____ 13

Fig. S5: Evaluation of AD-P activity under partially denaturing conditions. _____ 14

Fig. S6: Evaluation of AD-P activity under different cleavage conditions. _____ 16

Fig. S7: Installation of SChex and SCpen modifications on H3K9C and subsequent cleavage by AD-P. _____ 17

Fig. S8: Installation of SPh and SF_2Bn modifications on H3K9C and subsequent cleavage by AD-P. 19

Fig. S9: Installation of isoHis and SAllyl modifications on H3K9C and subsequent cleavage by AD-P. _____ 21

Fig. S10: *Installation of SClp and SAlkyne modifications on H3K9C and subsequent cleavage by AD-P.* _____ 23

Fig. S11: *Installation of $K_s$ac modification on H3K9C and subsequent cleavage by AD-P.* _____ 24

Fig. S12: Cleavage of SLeu-modified H3 variants (K9-SLeu, K27-SLeu, K36-SLeu, K79-SLeu) by AD-P. _____ 25

Fig. S13: Cleavage of SLeu-modified H3 variants (K9-SLeu and K27-SLeu) by AD-P. _____ 26

Fig. S14: Cleavage of SLeu-modified H3 variants (K36-SLeu and K79-SLeu) by AD-P. _____ 27

Fig. S15: Cleavage of $-SCH_2CF_3$-modified H3 variants (K9-SCH_2CF_3 and K27-SCH_2CF_3) by AD-P. _____ 29

Fig. S16: Cleavage of -SCH_2CF_3 modified H3 variants (K36-SCH_2CF_3 and K79-SCH_2CF_3) by AD-P. ___32

Fig. S17: Cleavage of dimethyllysine analog H3 variants (K9$_S$me$_2$ and K27$_S$me$_2$) by AD-P. ___34

Fig. S18: Cleavage of dimethyl-lysine analog H3 variants (K36$_S$me$_2$ and K79$_S$me$_2$) by AD-P. ___36

Fig. S19: Stereoselective preparation of H3K36-*L*-SCH_2CF_3. ___37

Fig. S20: Chemical modification process of H4$_{15\text{-}25}$K20C to form epimeric H4-SBn. ___39

Fig. S21: Confirmation of D-stereoselectivity in AD-P cleavage. ___40

Fig. S22: NMR assignment of diastereotopic peptide peaks. ___41

Fig. S23: Purification of GFP-M233C and installation of Dha. ___44

Fig. S24: Incorporation of -SBn modification on GFP-M233C and subsequent cleavage by AD-P. ___46

Fig. S25: Cleavage of Sallyl and $_S$Glc modifications on GFP by AD-P. ___47

Fig. S26: Incorporation of SF_2Bn modification on Ddx4^N and subsequent cleavage by AD-P. ___49

Fig. S27: Incorporation of -SCH_2CF_3 modification on Trx-SGTA^C and subsequent cleavage by AD-P. ___50

Fig. S28: Incorporation of -SCH_2CF_3 PTM on Trx-SGTA^C protein and Stereoselective preparation of Trx-SGTA^C-*L*-SCH_2CF_3. ___51

Experimental Procedures ___*53*

Materials and General Methods ___53

General Procedure for Reverse-Phase Chromatography ___53

Mass Spectrometry ___54

Methods ___54

Protein and peptide preparation ___54

Site-directed mutagenesis of GFP, Ddx4^N and H3 proteins ___54

AD-P expression and purification ___54

H3 protein expression and purification ___55

GFP expression and purification ___56

Ddx4^N-F182C expression and purification ___56

Trx-SGTA^C protein expression and purification ___57

Peptide synthesis and purification0020 ___57

Chemical mutagenesis of peptide and proteins ___58

Preparation of H4 K20-L/D-SBn ___58

Preparation of H4K20-L-SBn ____ 58

Fig. S29: General scheme for alkylation of $H4_{15-25}$ K20C. ____ 58

Chemical mutagenesis on H3 variants (K9C, K27C, K36C, K79C) ____ 59

Installing Dha ____ 59

Functionalizing Dha with trifluoroethane thiol (-SCH_2CF_3) ____ 59

Functionalising H3 (K9C, K27C, K36C, K79C)-Dha with other thiol reagents ____ 60

Chemical mutagenesis on GFP-M233C ____ 62

Installing Dha ____ 62

Functionalising Dha with benzyl mercaptan ____ 63

GFP-SAllyl ____ 63

GFP_SGlc ____ 63

Chemical mutagenesis on Trx-$SGTA^C$ protein ____ 63

Installing Dha ____ 63

Chemical mutagenesis on $Ddx4^N$-F182C ____ 64

Installing Dha ____ 64

Fig. S30: HPLC analysis of chemical modifications of H3K9 species. ____ 65

Fig. S31: Analysis of chemical modifications of H3K27 species. ____ 66

Fig. S32: Analysis of chemical modifications of H3K36. ____ 67

Fig. S33: Analysis of chemical modifications of H3K79 species. ____ 68

Cleavage reactions with AD-P ____ 69

Analytical cleavage of H3 variants by AD-P ____ 69

Preparative cleavage of H3 variants by AD-P ____ 69

$H4K20_{15-25}SBn$ peptide cleavage with AD-P ____ 69

GFP-SBn cleavage by AD-P ____ 70

GFP-SAllyl cleavage by AD-P ____ 70

GFP_SGlc cleavage by AD-P ____ 70

Analytical cleavage of $Ddx4^N$-SF_2Bn by AD-P ____ 70

Analytical cleavage of Trx-$SGTA^C$-SCH_2CF_3 by AD-P ____ 70

Preparative cleavage of Trx-$SGTA^C$-SCH_2CF_3 by AD-P ____ 71

NMR analysis methods ____ 71

NMR analysis of diastereotopic peptides ____ 71

^{19}F-NMR analysis of H3K36-SCH_2CF_3 ____ 72

Synthesis of prop-2-ynyl *S*-thioacetate ____ 72

Fig. S34: ^{1}H-NMR spectra of purified prop-2-ynyl *S*-thioacetate rotated at 20 Hz in $CDCl_3$ at 298 K. ____ 73

Fig. S35: ^{13}C-NMR spectra of purified prop-2-ynyl *S*-thioacetate in $CDCl_3$ at 298 K. ____ 73

Fig. S36: HPLC analysis of prop-2-ynyl *S*-thioacetate. ____ 74

Synthesis of propargyl thiol ____ 74

Protein Sequences ____ 74

ADP-6His ____ 74

DNA sequence ____ 74

Protein sequence ____ 75

GFP-M233C ____ 75

H3K9C ____ 75

H3K27C ____ 75

H3K36C ____ 76

H3K79C ____ 76

Trx-SGTA^{C} (residues 213-313) ____ 76

Ddx4^{N}-F182C (residues 1-236) ____ 76

Supplementary References ____ 76

Supporting Tables

Table S1: Overview of conditions for the cleavage of peptides and proteins containing D-amino acid by Alkaline D-Peptidase (AD-P).

	Conditions		Substrate	Cleavage efficiency	Data
**Default conditions** (HRMS)	0.1 M Sodium Carbonate, pH 9.3 30 °C, overnight, 84 µM Substrate, 7 µM AD-P		GFP-SAllyl	**	Fig. 5b
			GFP-SBn (2h)	***	Fig. 5a
			H3K9-SBn	***	Fig. 2, S2
**Buffer** (HRMS)	0.1 M HEPES, pH 8 (4h)		H3K9-SBn	*	Fig. S6i
**Temperature** (HRMS)	4 °C (1h)		H3K9-SLeu	***	Fig. S6a,g
	30 °C (1h)		H3K9-SLeu	***	Fig. S6a,b
	40 °C (1h)		H3K9-SLeu	**	Fig. S6a,h
**L-Arg** (HRMS)	0 mM (1h)		H3K9-SLeu	***	Fig. S6a,c
	50 mM (1h)		H3K9-SLeu	***	Fig. S6a,b
	100 mM (1h)		H3K9-SLeu	**	Fig. S6a,d
	167 mM (1h)		H3K9-SLeu	**	Fig. S6a,e
**Ionic strength** (HRMS)	500 mM KCL (1h)		H3K9-SLeu	**	Fig. S6f
**Denaturant** (HPLC)	35 µM Substrate, 7 µM AD-P, 15 min	0 M GdmCl	Orexin B peptide analog	**	Fig. S5a
		0.1 M GdmCl		*	
		0.25 M GdmCl		*	
		0.5 M GdmCl		-	
		1 M GdmCl		-	
**Solvents** (HPLC)	35 µM Substrate, 7 µM AD-P, 30 min	No solvent	Orexin B peptide	***	Fig. S5b,c
		DMF (1%)		**	
		DMF (5%)		-	
		DMSO (1%)		**	
		DMSO (5%)		**	
**Time**	84 µM Substrate, 3.7 µM AD-P (SDS-PAGE)	5 mins	H3K9-SBn	**	Fig. S4
		15 mins		**	
		30 mins		***	
		60 mins		***	
	217 µM Substrate, 20.4 µM AD-P (HPLC)	60 min	$H4_{15\text{-}25}$K20-SBn	*	Fig. S21c,d
		2 h		**	
		3 h		**	
	347 µM Substrate, 20.4 µM AD-P (HPLC)	Overnight		***	Fig. 4b,c

Standard conditions are indicated in the top row. Additional information on the effect of pH and temperature on the activity of AD-P has been reported previously.[1] Cleavage efficiencies were approximated from HPLC peak area (peptides), MS peak intensity (proteins) or SDS-PAGE band intensity (proteins) as follows: - for <10%; * for 10-50%; ** for 50-90%; *** for >90% cleavage based on estimated amounts of D-isomer in the sample.

Table S2: Primers used in this study.

No.	Primer name	Sequence
1	GFP-C70M-F	TGGTGTTCAAATGTTCAGCAGGTACCCTGAC
2	GFP-C70M-R	TAAGTCAGGGTGGTCACC
3	GFP-His-C70M/C48S-F	GAAGTTCATTTCTACAACAGGAAAG
4	GFP-His-C70M/C48S-R	AGAGTCAGCTTTCCATTAG
5	(GFP-His-C70M/C48S)-M233C-F	TACTCTGGGATGTGATGAGCTGTACAAGAGC
6	(GFP-His-C70M/C48S)-M233C-R	ATTCCTGCTGCTGTCACA
7	H3K9C-F	AACCGCGCGTTGCTCCACCGGCG
8	H3K9C-R	TGTTTGGTACGAGCCATATG
9	H3K27C-R	TTGGTCGCCAGCTG
10	H3K27C-F	AGCGGCACGCTGCTCTGCGCCTG
11	H3K36C-R	GTCGCAGGCGCAGAT
12	H3K36C-F	CGGTGGTGTGTGCAAACCGCACC
13	H3K79C-F	GCAGGACTTCTGCACGGACCTGC
14	H3K79C-R	GCGATTTCACGTACCAG
15	Ddx4-F182C-R	GTGCGCTGCATGCTTTCATCCGGGTCCAG
16	Ddx4-F182C-F	CGGCGGCCTGTGTGGCAGCCGCC

Supporting Figures

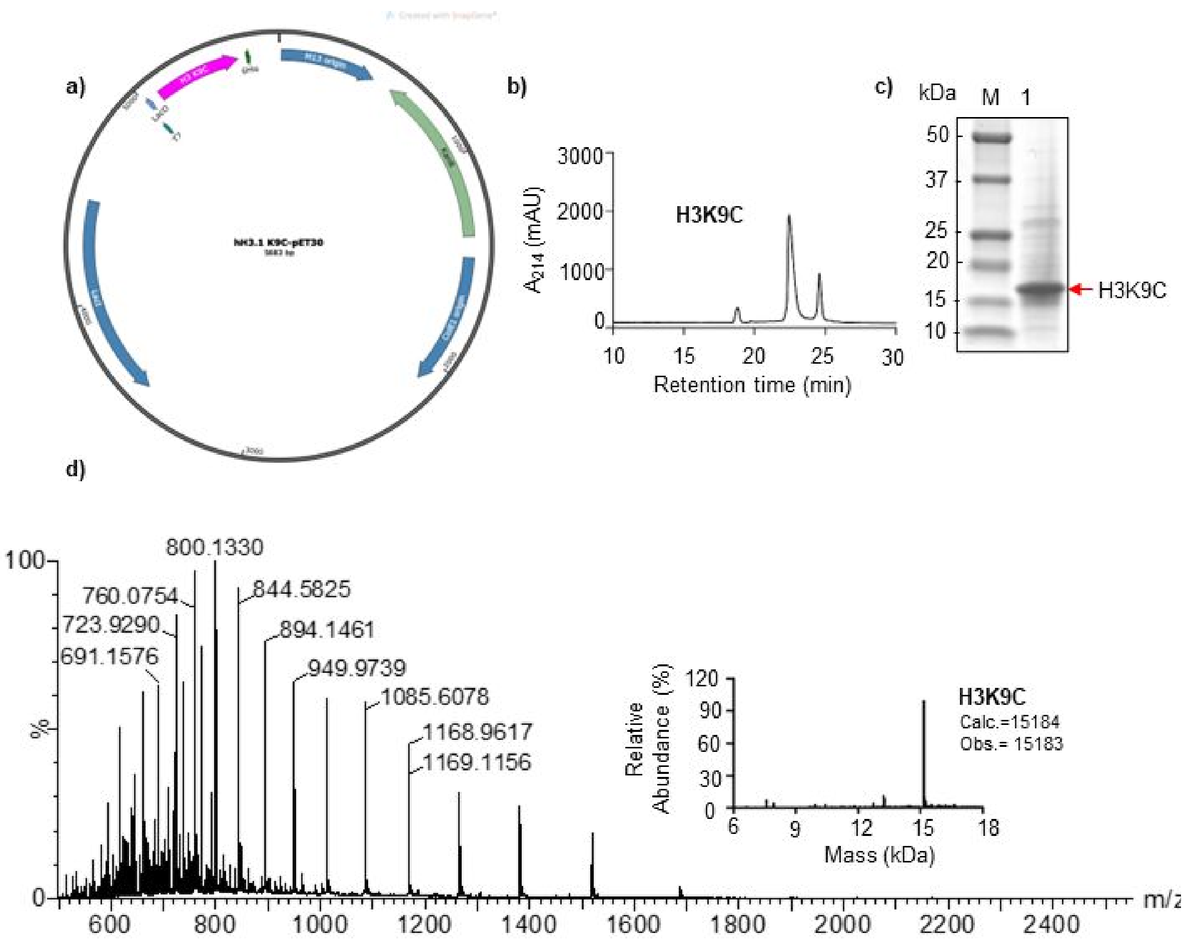

Fig. S1: Production of H3 K9C.

(a) Schematic diagram illustrating the expression vector pET-30 H3K9C. (b) HPLC analysis of H3K9C. (c) SDS-PAGE analysis of purified H3K9C protein. Lane M - Protein marker; Lane 1 - H3K9C protein. (d) Mass spectrum of H3K9C (calculated mass = 15184 Da; observed mass = 15183 Da).

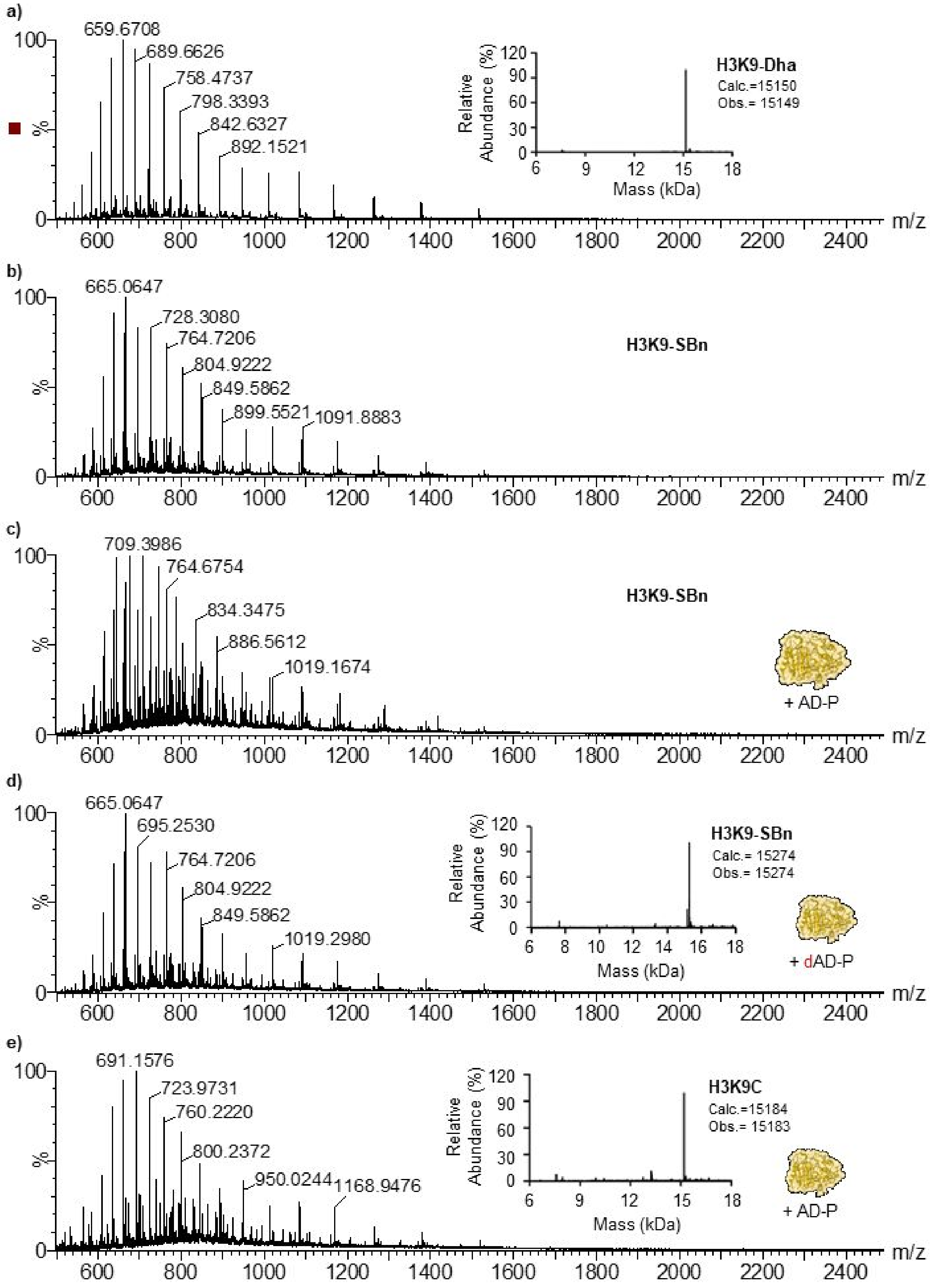

a)
659.6708
689.6626
758.4737
798.3393
842.6327
892.1521
H3K9-Dha
Calc.=15150
Obs.= 15149
Relative Abundance (%)
Mass (kDa)
m/z
b)
665.0647
728.3080
764.7206
804.9222
849.5862
899.5521
1091.8883
H3K9-SBn
c)
709.3986
764.6754
834.3475
886.5612
1019.1674
H3K9-SBn
+ AD-P
d)
665.0647
695.2530
764.7206
804.9222
849.5862
1019.2980
H3K9-SBn
Calc.= 15274
Obs.= 15274
+ dAD-P
e)
691.1576
723.9731
760.2220
800.2372
950.0244
1168.9476
H3K9C
Calc.=15184
Obs.= 15183
+ AD-P

Fig. S2: Installation of benzyl modification on H3K9C and subsequent cleavage by AD-P.

(a) Mass spectrum of H3K9-Dha (calculated mass = 15150 Da, observed mass = 15149 Da. (b,c) MS analysis of epimeric H3K9-SBn (top) and cleavage products (bottom). For the H3K9-SBn L/D-isomer mixture, calculated mass = 15274 Da, observed mass = 15273 Da. For C-terminal cleaved D-isomer, calculated mass = 14168 Da, observed mass = 14167 Da. (d) Mass spectrum of H3K9-SBn incubated with denatured AD-P (dAD-P), calculated mass (H3K9-SBn) = 15274 Da, observed mass = 15274 Da). (e) Mass spectrum of H3K9C and AD-P cleavage (calculated mass = 15184 Da, observed mass = 15183 Da).

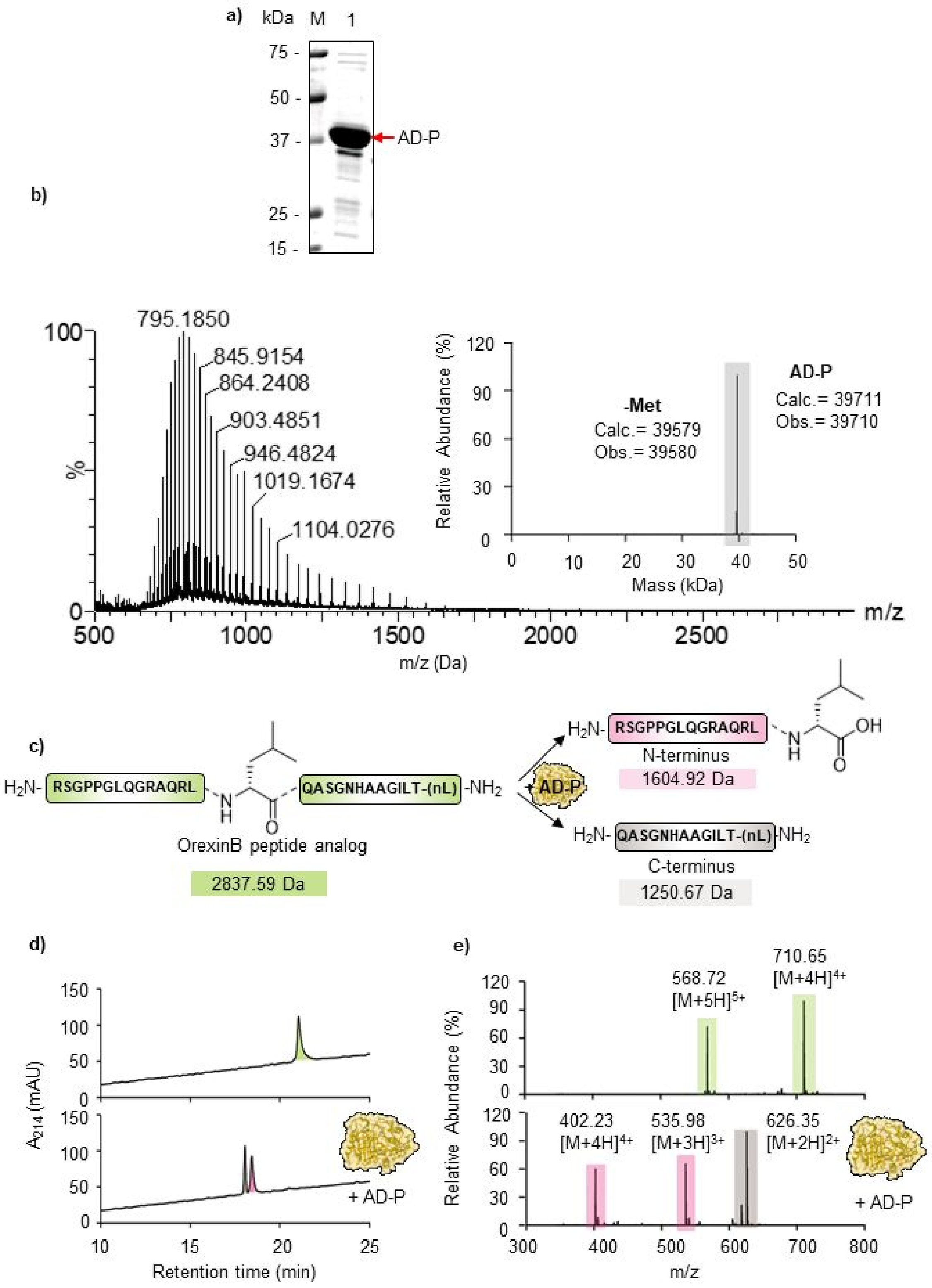

a)
kDa M 1
75 -
50 -
37 -
25 -
15 -
AD-P
b)
795.1850
845.9154
864.2408
903.4851
946.4824
1019.1674
1104.0276
100
%
0
500
1000
1500
2000
2500
m/z
m/z (Da)
Relative Abundance (%)
120
90
60
30
0
-Met
Calc.= 39579
Obs.= 39580
AD-P
Calc.= 39711
Obs.= 39710
0 10 20 30 40 50
Mass (kDa)
c)
H2N- RSGPPGLQGRAQRL
QASGNHAAGILT-(nL) -NH2
OrexinB peptide analog
2837.59 Da
AD-P
H2N- RSGPPGLQGRAQRL
OH
N-terminus
1604.92 Da
H2N- QASGNHAAGILT-(nL) -NH2
C-terminus
1250.67 Da
d)
150
100
50
0
A214 (mAU)
+ AD-P
10 15 20 25
Retention time (min)
e)
568.72
[M+5H]5+
710.65
[M+4H]4+
402.23
[M+4H]4+
535.98
[M+3H]3+
626.35
[M+2H]2+
Relative Abundance (%)
120
90
60
30
0
+ AD-P
300 400 500 600 700 800
m/z

Fig. S3: Purification and activity test of alkaline D-peptidase (AD-P).

(a) SDS-PAGE analysis of AD-P purification by Ni-NTA chromatography. Lane M - Protein marker; Lane 1 – AD-P. (b) Mass spectrum of AD-P (calculated mass Δ1-32 = 39711 Da, observed mass = 39710 Da). Of note, previous studies[2] have indicated that the N-terminal 32 residues of AD-P represent a signal peptide that is cleaved during protein production, without affecting the enzymatic activity of AD-P. (c) Peptide sequence of an OrexinB analog and schematic diagram illustrating peptide cleavage by AD-P. nL = norleucine. (d) HPLC analysis of OrexinB analog before (top) and after cleavage by AD-P (bottom). (e) HRMS analysis of OrexinB analog before (top) and after cleavage by AD-P (bottom). Calculated mass of OrexinB analog = 2838.59 Da, observed mass = 2838.57 Da; Calculated mass of the N-terminal cleavage product = 1604.92 Da, observed mass = 1604.92 Da (pink): Calculated mass for C-terminal cleavage product = 1250.67 Da, observed mass=1250.68 Da (grey).

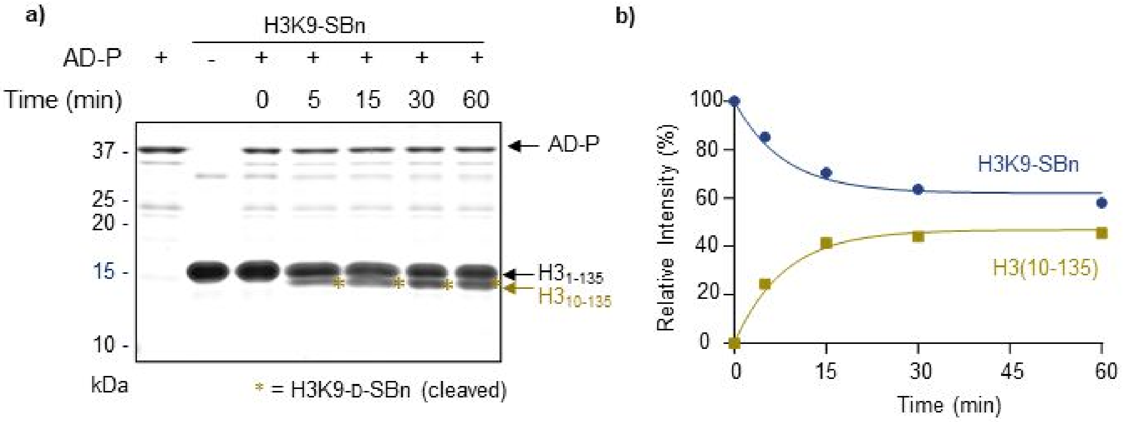

Fig. S4: Cleavage of H3K9-SBn by AD-P.

a) SDS-PAGE monitoring of the time course of the H3K9-SBn cleavage reaction. C-terminal cleavage products are indicated by a beige asterisk. (b) Quantification of the cleavage of H3K9-SBn by AD-P based on SDS-PAGE analysis (full-length H3K9-SBn: blue, cleaved $H3_{10-135}$: beige). Time course reactions were quantified by measuring band intensities with ImageJ, and subsequently calculating the relative percentages of the cleaved and uncleaved species. Relative concentrations were calculated by dividing the relative band intensities by the molecular weight of the cleaved and uncleaved species. The reaction progress was fit to an exponential decay function (solid lines), yielding an apparent rate constant k_{app} = 0.092 – 0.17 min^{-1} (95% CI).

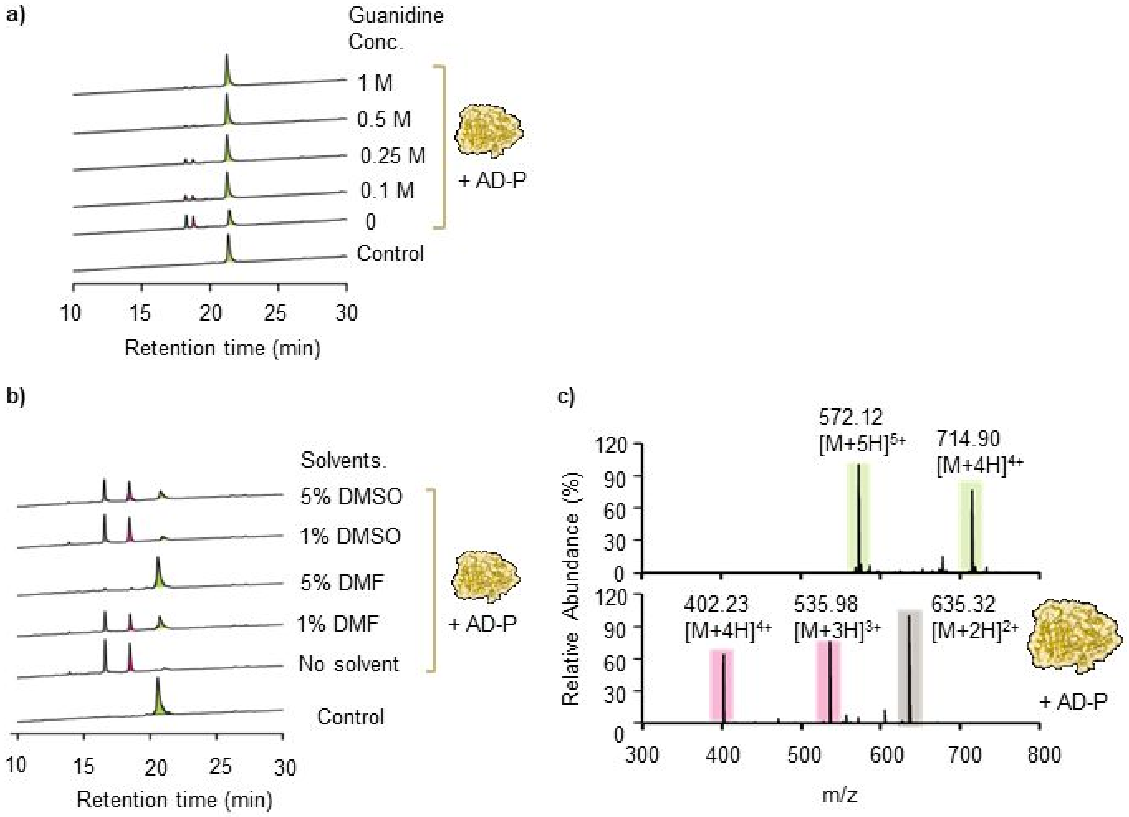

Fig. S5: Evaluation of AD-P activity under partially denaturing conditions.

(a) HPLC monitoring of AD-P activity on a D-Leu-containing isomer of OrexinB analog in the presence of varying concentrations of GdmCl, (b) HPLC monitoring of AD-P activity on the OrexinB peptide isomer in the presence of organic solvent dimethylformamide (DMF) and dimethyl sulfoxide (DMSO). (c) HRMS analysis of OrexinB before (top) and after cleavage by AD-P (bottom). The orexin B peptide sequence used in this experiment corresponded to: RSGPPGLQGRAQRL-D-Leu-QASGNHAAGILTM-NH_2, OrexinB: calculated mass = 2855.54 Da, observed mass = 2855.6 Da (green bars); *N*-terminal cleavage product: calculated mass = 1604.92 Da, observed mass = 1604.94 Da (pink bars); *C*-terminal cleavage product: calculated mass = 1268.63 Da, observed mass = 1268.64 Da (grey bar).

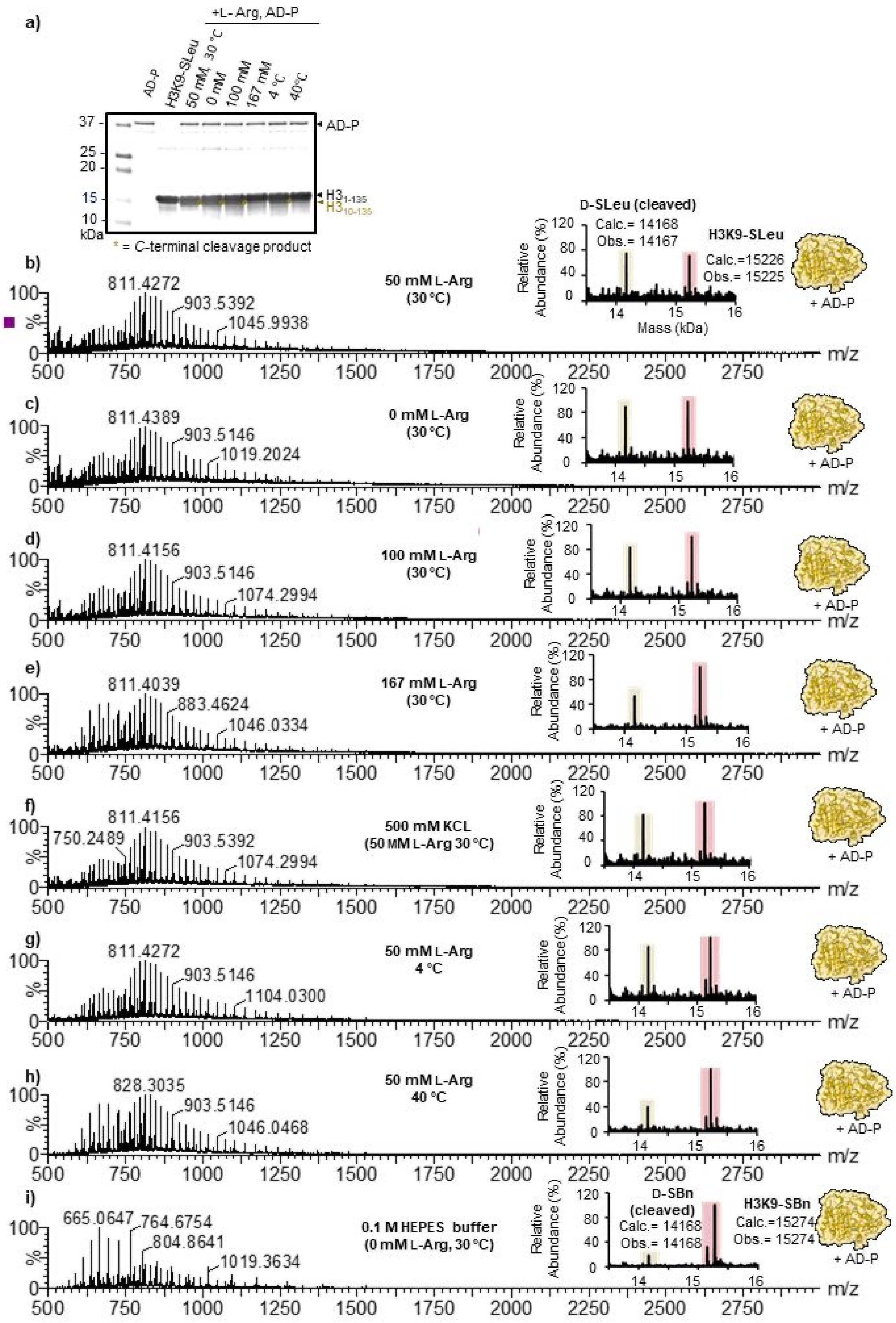
a)
+L-Arg, AD-P
AD-P
H3K9-SLeu
50 mM, 30 °C
0 mM
100 mM
167 mM
4 °C
40°C
37
25
20
15
10
kDa
AD-P
H3 1-135
H3 10-135
* = C-terminal cleavage product
b)
811.4272
903.5392
1045.9938
50 mM L-Arg (30 °C)
D-SLeu (cleaved)
Calc.= 14168
Obs.= 14167
H3K9-SLeu
Calc.=15226
Obs.= 15225
Relative Abundance (%)
Mass (kDa)
+ AD-P
m/z
c)
811.4389
903.5146
1019.2024
0 mM L-Arg (30 °C)
d)
811.4156
903.5146
1074.2994
100 mM L-Arg (30 °C)
e)
811.4039
883.4624
1046.0334
167 mM L-Arg (30 °C)
f)
811.4156
750.2489
903.5392
1074.2994
500 mM KCL (50 MM L-Arg 30 °C)
g)
811.4272
903.5146
1104.0300
50 mM L-Arg 4 °C
h)
828.3035
903.5146
1046.0468
50 mM L-Arg 40 °C
i)
665.0647
764.6754
804.8641
1019.3634
0.1 M HEPES buffer (0 mM L-Arg, 30 °C)
D-SBn (cleaved)
Calc.= 14168
Obs.= 14168
H3K9-SBn
Calc.=15274
Obs.= 15274

Fig. S6: Evaluation of AD-P activity under different cleavage conditions.

a) SDS-PAGE analysis of AD-P's cleavage efficiency on H3K9-SLeu. C-terminal cleavage products are indicated by a beige asterisk. (b-h) Mass spectrum of AD-P cleavage on H3K9-SLeu: (b) cleavage reaction with L-Arg final concentration = 50 mM; (c) 0 mM; (d) 100 mM; (e) 167 mM; (f) 50 mM L-Arg, 500 mM KCl. The reaction temperature for these reactions was 30 °C. (g) and (h) show mass spectrum analyses of AD-P cleavage on H3K9-SLeu under 4 °C and 40 °C, respectively, with final concentration of L-Arg = 50 mM. (i) Mass spectrum analysis of AD-P cleavage on H3K9-SBn in 0.1 M HEPES buffer pH 8.0, under 30 °C. Calculated mass for C-terminal cleavage product = 14168 Da, observed mass (for (b-h)) = 14167 Da, observed mass (for (i)) = 14168 Da. Colored bars correspond to C-terminal cleaved product (beige) and uncleaved species after kinetic resolution (red).

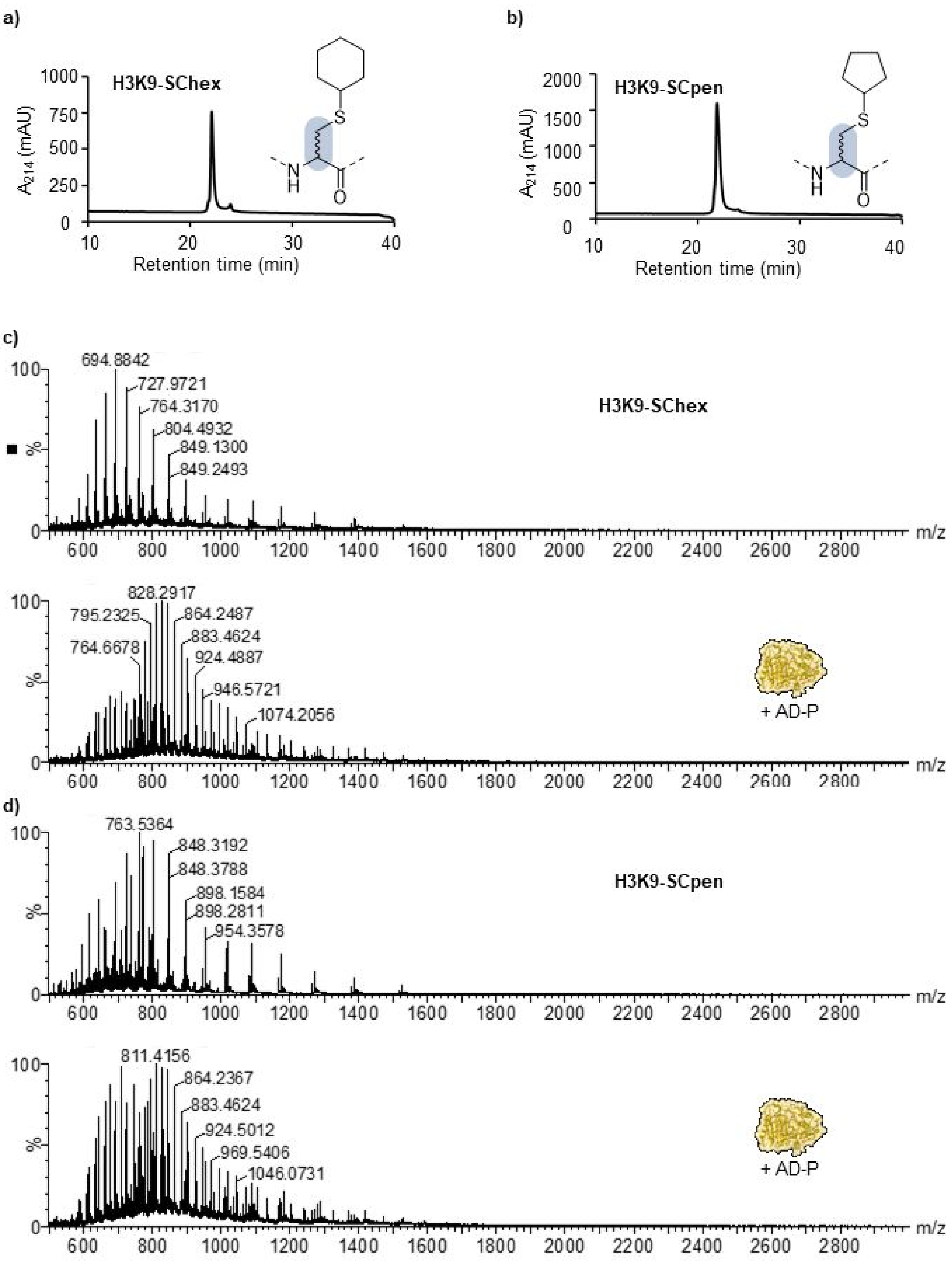

Fig. S7: Installation of SChex and SCpen modifications on H3K9C and subsequent cleavage by AD-P.

(a) HPLC analysis of H3K9-SChex. (b) HPLC analysis of H3K9-SCpen. (c) Mass spectrum of AD-P cleavage on H3K9-SChex. Control (top): calculated mass = 15266 Da, observed mass = 15266 Da.

C-terminal cleaved product (bottom): calculated mass = 14168 Da, observed mass = 14168 Da. (d) Mass spectrum of AD-P cleavage on H3K9-SCpen. Control (top): calculated mass = 15252 Da, observed mass = 15252 Da. *C*-terminal cleaved product (bottom): calculated mass = 14168 Da, observed mass = 14168 Da.

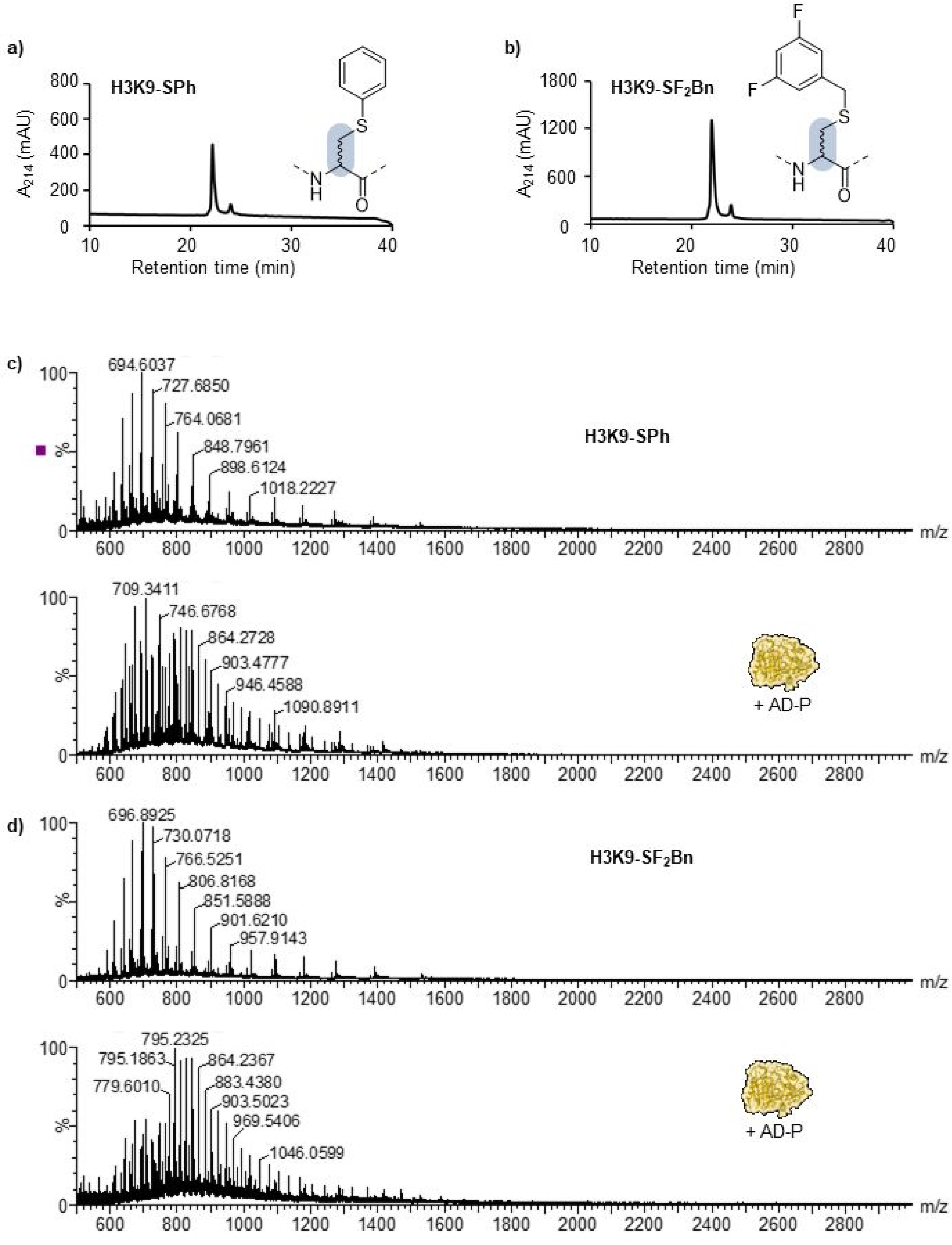

Fig. S8: Installation of SPh and SF$_2$Bn modifications on H3K9C and subsequent cleavage by AD-P.

(a) HPLC analysis of H3K9-SPh. (b) HPLC analysis of H3K9-SF$_2$Bn. (c) Mass spectrum of AD-P cleavage on H3K9-SPh. Control (top): calculated mass = 15260 Da, observed mass = 15260 Da.

C-terminal cleaved product (bottom): calculated mass = 14168 Da, observed mass = 14168 Da. (d) Mass spectrum of AD-P cleavage on H3K9-SF_2Bn. Control (top): calculated mass = 15310 Da, observed mass = 15310 Da. *C*-terminal cleaved product (bottom): calculated mass = 14168 Da, observed mass = 14168 Da.

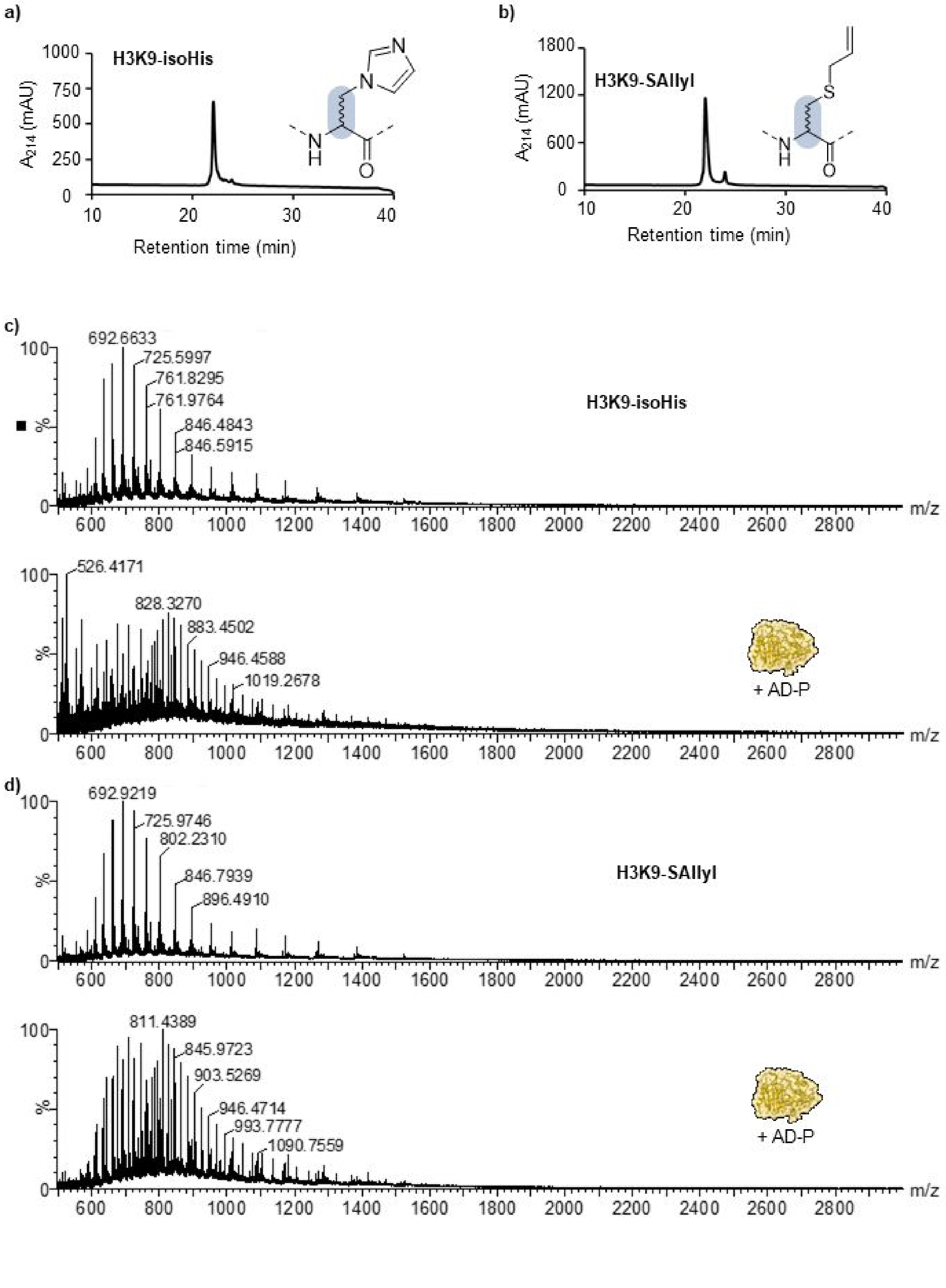

Fig. S9: Installation of isoHis and SAllyl modifications on H3K9C and subsequent cleavage by AD-P.

(a) HPLC analysis of H3K9-isoHis. (b) HPLC analysis of H3K9-SAllyl. (c) Mass spectrum of AD-P cleavage on H3K9-isoHis. Control (top): calculated mass = 15218 Da, observed mass = 15219 Da.

C-terminal cleaved product (bottom): calculated mass = 14168 Da, observed mass = 14168 Da. (d) Mass spectrum of AD-P cleavage on H3K9-SAllyl. Control (top): calculated mass = 15224 Da, observed mass = 15224 Da. *C*-terminal cleaved product (bottom): calculated mass = 14168 Da, observed mass = 14168 Da.

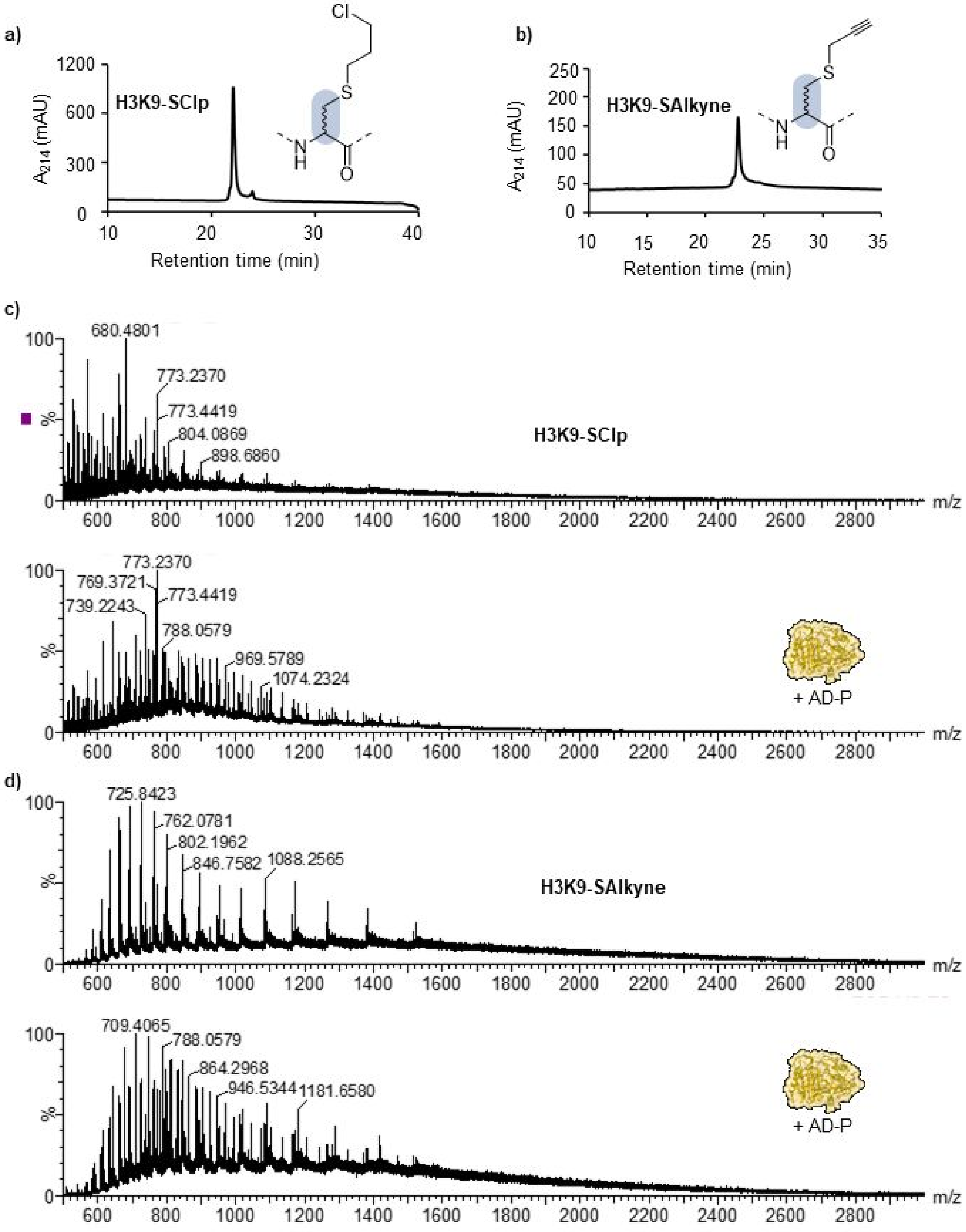

Fig. S10: ***Installation of SClp and SAlkyne modifications on H3K9C and subsequent cleavage by AD-P.***

(a) HPLC analysis of H3K9-SClp. (b) HPLC analysis of H3K9-SAllyne. (c) Mass spectrum of AD-P cleavage on H3K9-SClp. Control (top): calculated mass = 15259 Da, observed mass = 15260 Da. *C*-terminal cleaved product (bottom): calculated mass = 14168 Da, observed mass = 14168 Da. (d) Mass spectrum of AD-P cleavage on H3K9-SAllyne. Control (top): calculated mass = 15222 Da, observed mass = 15222 Da. C-terminal cleaved product (bottom): calculated mass = 14168 Da, observed mass = 14168 Da.

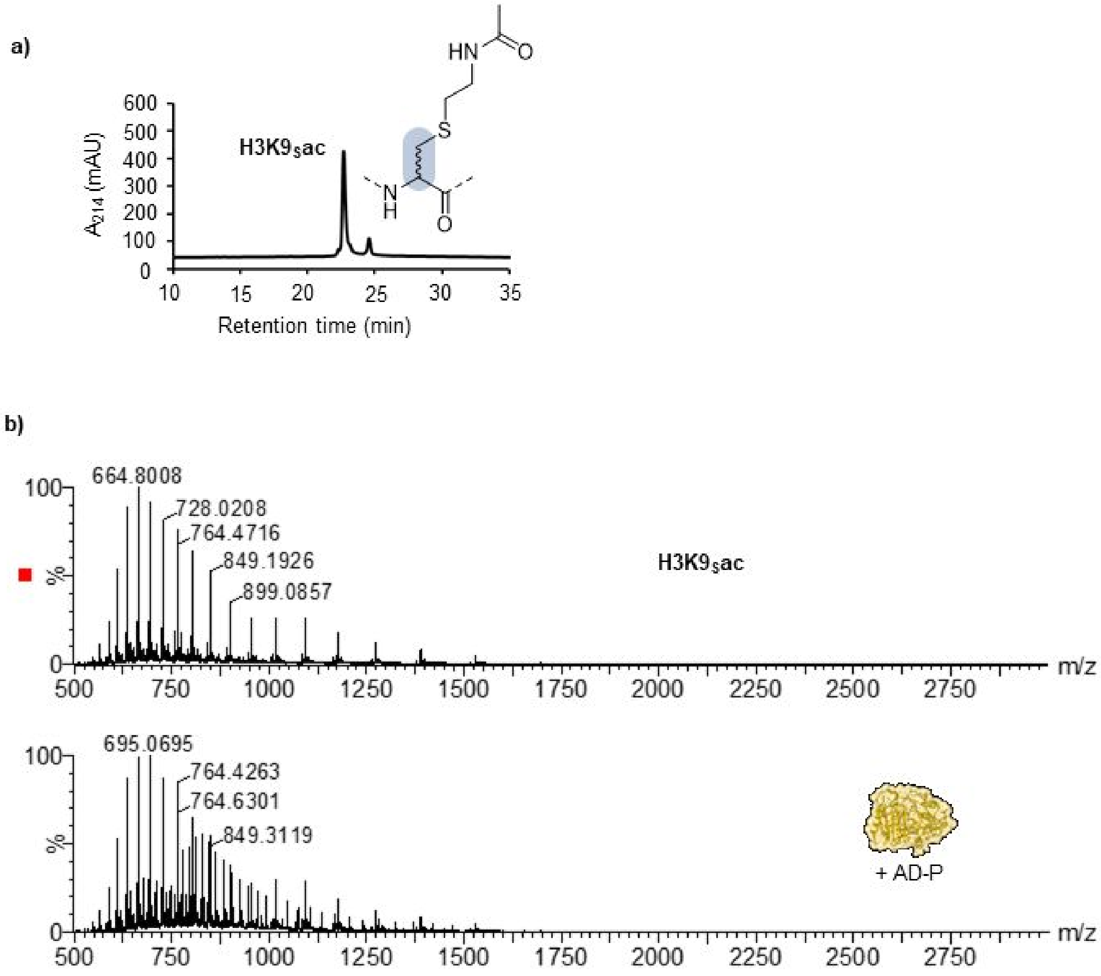

Fig. S11: ***Installation of $K_s$ac modification on H3K9C and subsequent cleavage by AD-P.***

(a) *HPLC analysis of H3K9$_S$ac* (b) Mass spectrum of AD-P cleavage on *H3K9$_S$ac*. Control (top): calculated mass = 15269 Da, observed mass = 15269 Da; *C*-terminal cleaved product (bottom): calculated mass = 14168 Da, observed mass = 14168 Da.

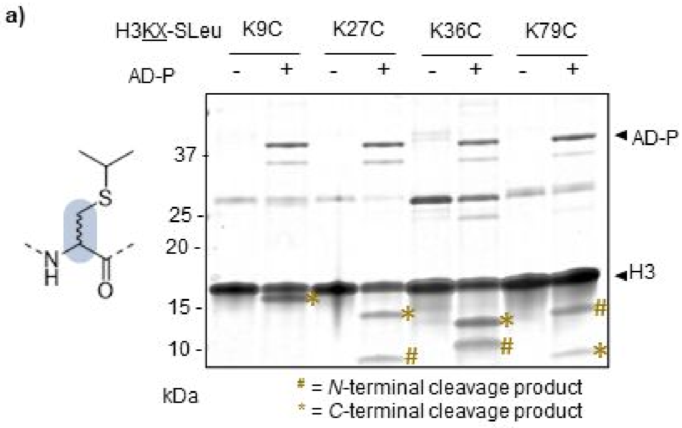

Fig. S12: Cleavage of SLeu-modified H3 variants (K9-SLeu, K27-SLeu, K36-SLeu, K79-SLeu) by AD-P.

SDS-PAGE analysis of SLeu modified H3 variants by AD-P. *N*- and *C*-terminal cleavage products are indicated by a brown hash and asterisk, respectively.

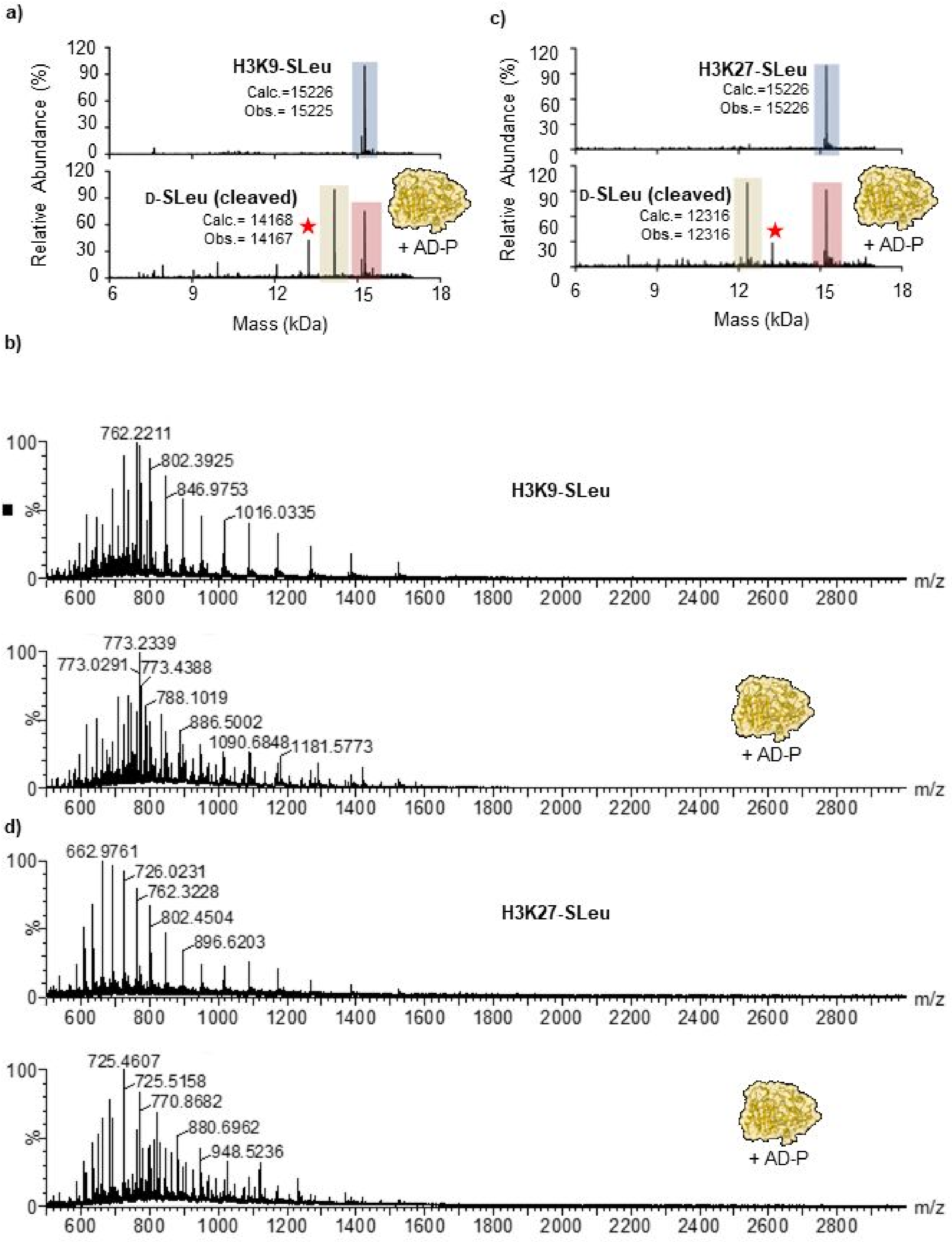

Fig. S13: Cleavage of SLeu-modified H3 variants (K9-SLeu and K27-SLeu) by AD-P.

(a,b) Mass spectrum of AD-P cleavage on H3K9-SLeu. Control (top): calculated mass = 15226 Da, observed mass = 15225 Da. *C*-terminal cleaved product (bottom): calculated mass = 14168 Da, observed mass = 14167 Da. (c,d) Mass spectrum of AD-P cleavage on H3K27-SLeu. Control (top): calculated mass = 15226 Da, observed mass = 15226 Da. C-terminal cleaved product (bottom): calculated mass = 12316 Da, observed mass = 12316 Da. The red star indicates a peak originating from AD-P. Colored bars correspond to epimeric starting material before AD-P treatment (blue), *C*-terminal cleaved product (beige) and uncleaved species after kinetic resolution (red).

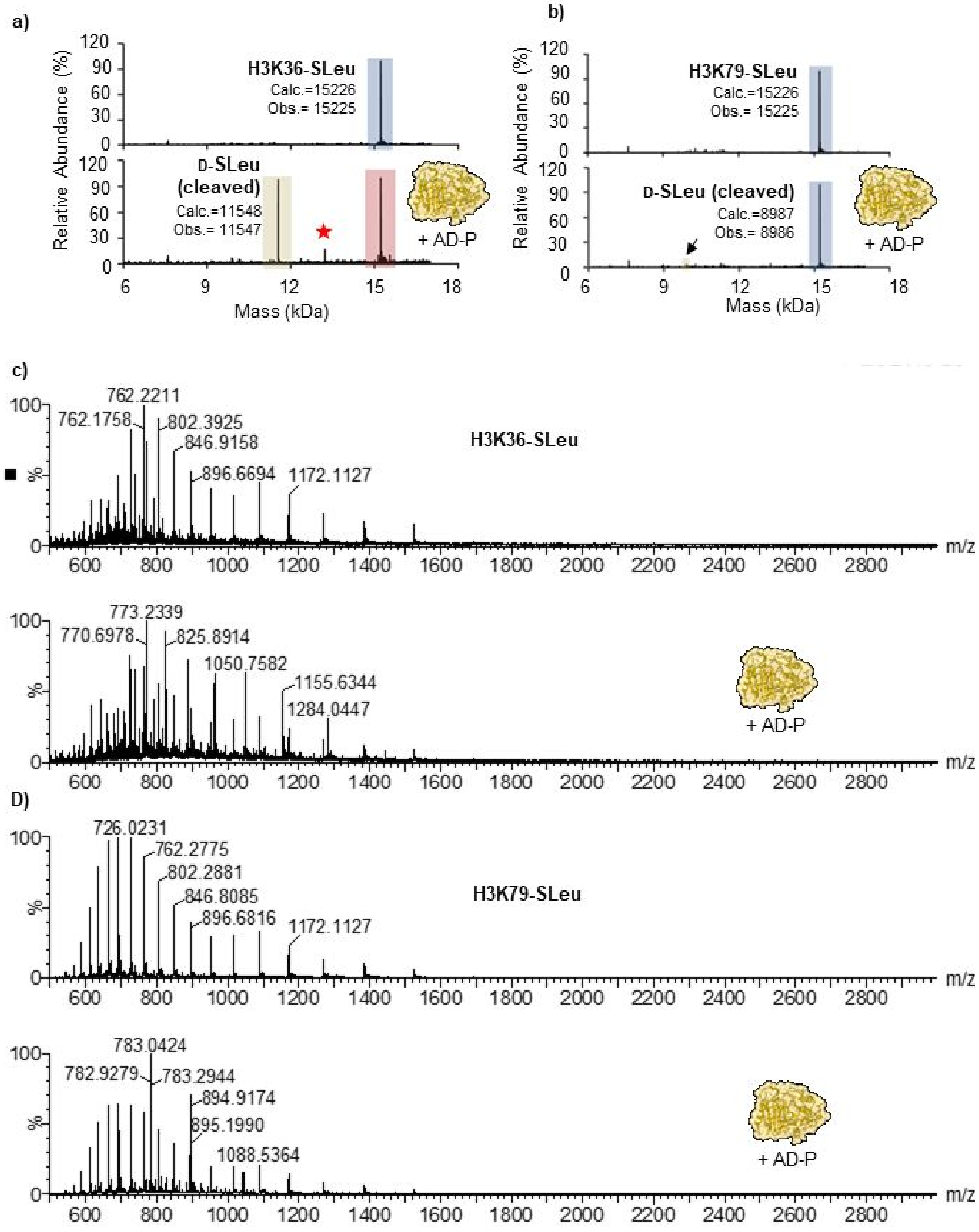

Fig. S14: Cleavage of SLeu-modified H3 variants (K36-SLeu and K79-SLeu) by AD-P.

(a,c) Mass spectrum of AD-P cleavage on H3K36-SLeu. Control (top): calculated mass = 15226 Da, observed mass = 15225 Da. C-terminal cleaved product (bottom): calculated mass = 11548 Da, observed mass = 11547 Da. (c,d) Mass spectrum of AD-P cleavage on H3K79-SLeu. Control (top):

calculated mass = 15226 Da, observed mass = 15225 Da. C-terminal cleaved product (bottom): calculated mass = 8987 Da, observed mass = 8986 Da. The red star indicates a peak originating from AD-P. Colored bars correspond to epimeric starting material before AD-P treatment (blue), C-terminal cleaved product (beige) and uncleaved species after kinetic resolution (red).

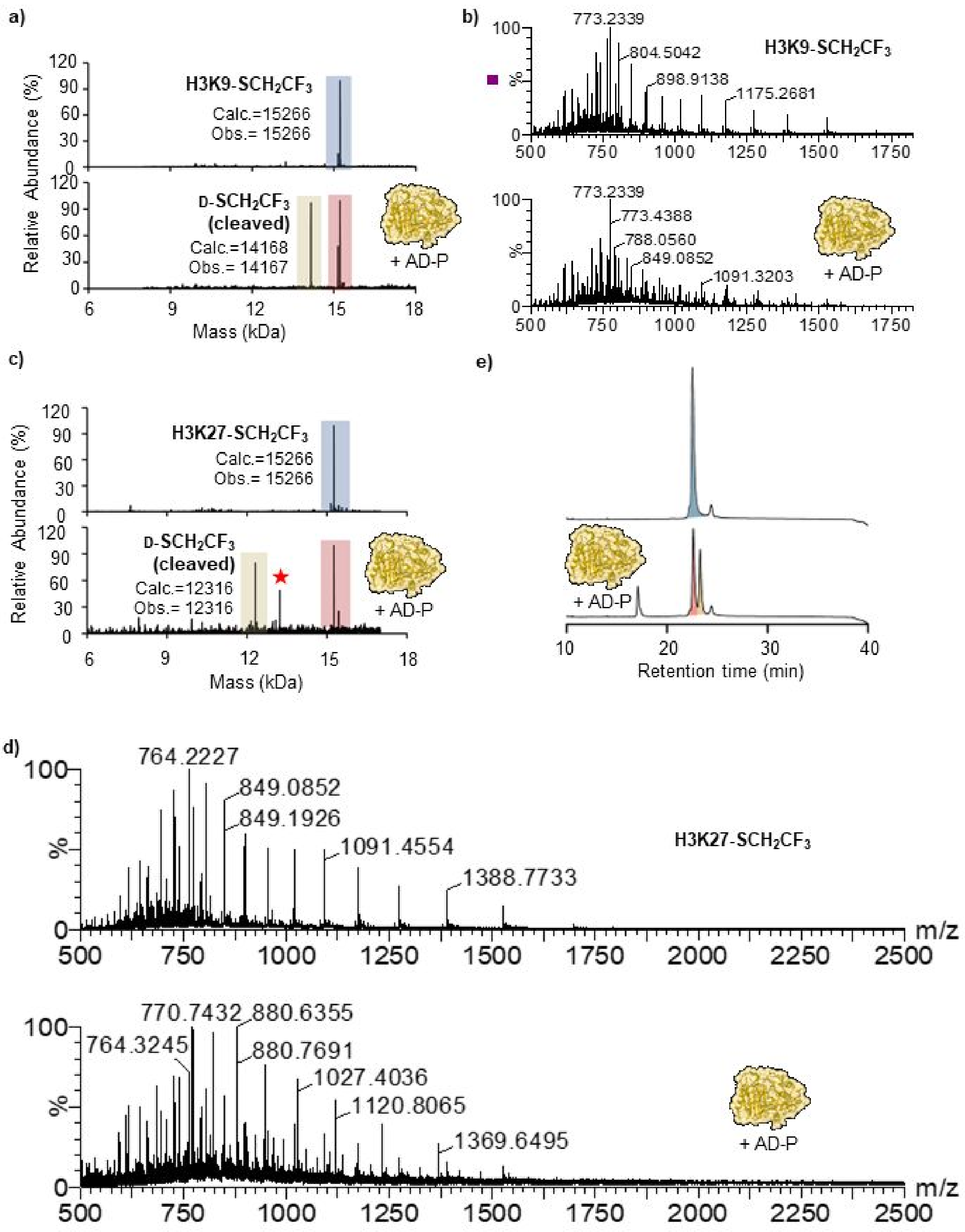

Fig. S15: Cleavage of -SCH_2CF_3-modified H3 variants (K9-SCH_2CF_3 and K27-SCH_2CF_3) by AD-P.

(a,b) Mass spectrum of AD-P cleavage on H3K9-SCH_2CF_3. Control (top): calculated mass = 15266 Da, observed mass = 15266 Da. C-terminal cleaved product (bottom): calculated mass = 14168 Da, observed mass = 14167 Da. (c,d) Mass spectrum of AD-P cleavage on H3K27-SCH_2CF_3. Control

(top): calculated mass = 15266 Da, observed mass = 15266 Da. C-terminal cleaved product (bottom): calculated mass = 12316 Da, observed mass = 12316 Da. The red star indicates a peak originating from AD-P. Colored bars correspond to epimeric starting material before AD-P treatment (blue), C-terminal cleaved product (beige) and uncleaved species after kinetic resolution (red). (e) HPLC analysis of AD-P cleavage on H3K27-SCH_2CF_3. Control (top) and AD-P cleavage on D-isomer (bottom). Epimeric mixtures are indicated in blue, enriched H3K27-L-SCH_2CF_3 in red, and cleaved D-isomer in beige.

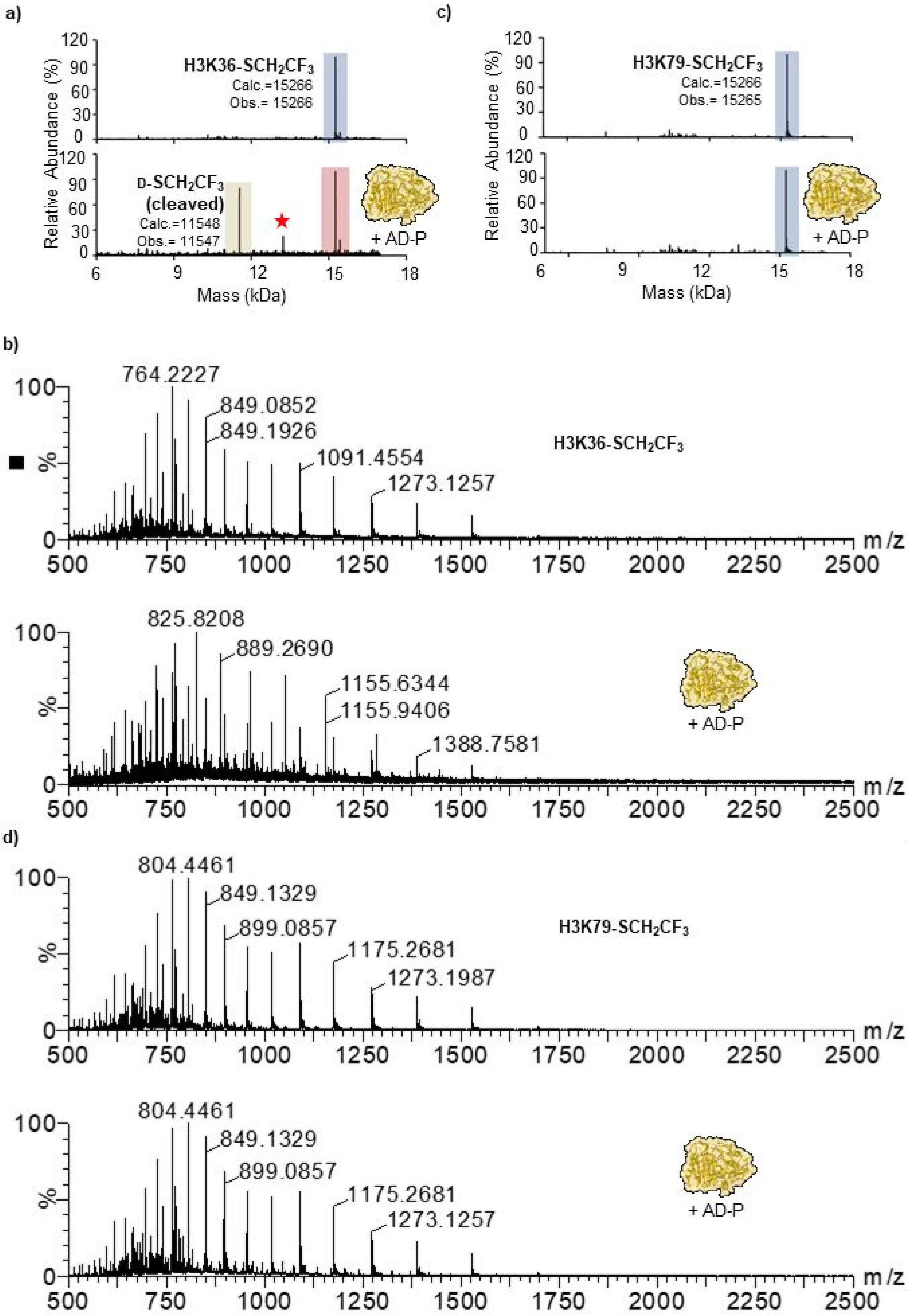
a)
H3K36-SCH$_2$CF$_3$
Calc.=15266
Obs.= 15266
D-SCH$_2$CF$_3$
(cleaved)
Calc.=11548
Obs.= 11547
+ AD-P
Relative Abundance (%)
Mass (kDa)
c)
H3K79-SCH$_2$CF$_3$
Calc.=15266
Obs.= 15265
+ AD-P
Relative Abundance (%)
Mass (kDa)
b)
764.2227
849.0852
849.1926
1091.4554
1273.1257
H3K36-SCH$_2$CF$_3$
825.8208
889.2690
1155.6344
1155.9406
1388.7581
+ AD-P
m/z
%
d)
804.4461
849.1329
899.0857
1175.2681
1273.1987
H3K79-SCH$_2$CF$_3$
804.4461
849.1329
899.0857
1175.2681
1273.1257
+ AD-P
m/z
%

Fig. S16: Cleavage of -SCH_2CF_3 modified H3 variants (K36-SCH_2CF_3 and K79-SCH_2CF_3) by AD-P.

(a,b) Mass spectrum of AD-P cleavage on H3K36-SCH_2CF_3. Control (top): calculated mass = 15266 Da, observed mass = 15266 Da. *C*-terminal cleaved product (bottom): calculated mass = 11548 Da, observed mass = 11547 Da). (c,d) Mass spectrum of AD-P cleavage on H3K79-SCH_2CF_3. Control (top): calculated mass = 15266 Da, observed mass = 15265 Da. N-terminal cleaved product (bottom): calculated mass = 8987 Da (not observed). The red star indicates a peak originating from AD-P. Colored bars correspond to epimeric starting material before AD-P treatment (blue), C-terminal cleaved product (beige) and uncleaved species after kinetic resolution (red).

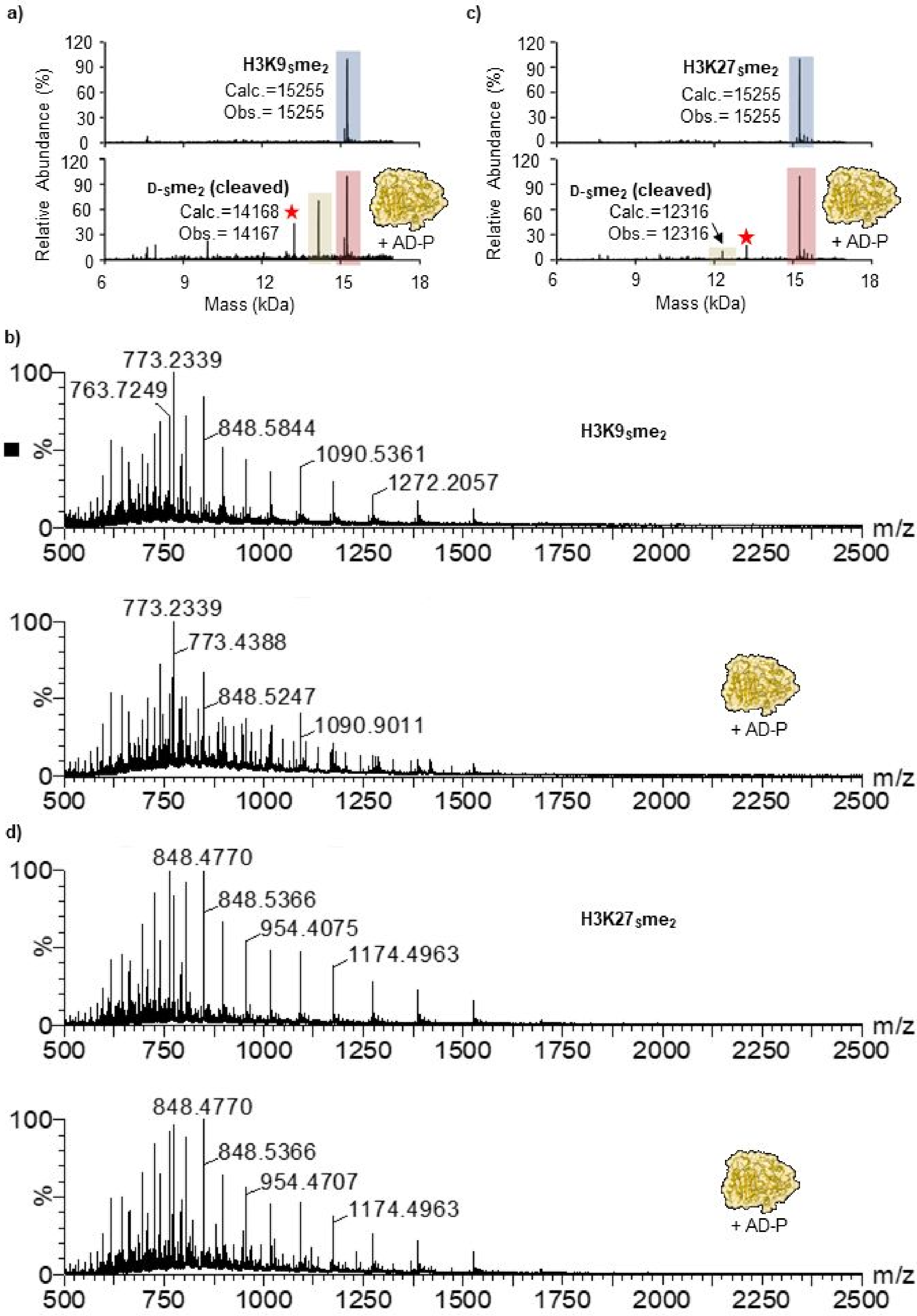
a)
c)
b)
d)
Relative Abundance (%)
H3K9$_S$me$_2$
Calc.=15255
Obs.= 15255
D-$_S$me$_2$ (cleaved)
Calc.=14168
Obs.= 14167
+ AD-P
Mass (kDa)
H3K27$_S$me$_2$
Calc.=15255
Obs.= 15255
D-$_S$me$_2$ (cleaved)
Calc.=12316
Obs.= 12316
773.2339
763.7249
848.5844
1090.5361
1272.2057
773.4388
848.5247
1090.9011
848.4770
848.5366
954.4075
1174.4963
954.4707
m/z
%

Fig. S17: Cleavage of dimethyllysine analog H3 variants ($K9_Sme_2$ and $K27_Sme_2$) by AD-P.

(a,b) Mass spectrum of AD-P cleavage of $H3K9_Sme_2$. Control (top): calculated mass = 15255 Da, observed mass = 15255 Da. C-terminal cleaved product (bottom): calculated mass = 14168 Da, observed mass = 14167 Da. (c,d) Mass spectrum of AD-P cleavage of $H3K27_Sme_2$. Control (top): calculated mass = 15255 Da, observed mass = 15255 Da. C-terminal cleaved product (bottom): calculated mass = 12316 Da, observed mass = 12316 Da. The red star indicates a peak originating from AD-P. Colored bars correspond to epimeric starting material before AD-P treatment (blue), *C*-terminal cleaved product (beige) and uncleaved species after kinetic resolution (red).

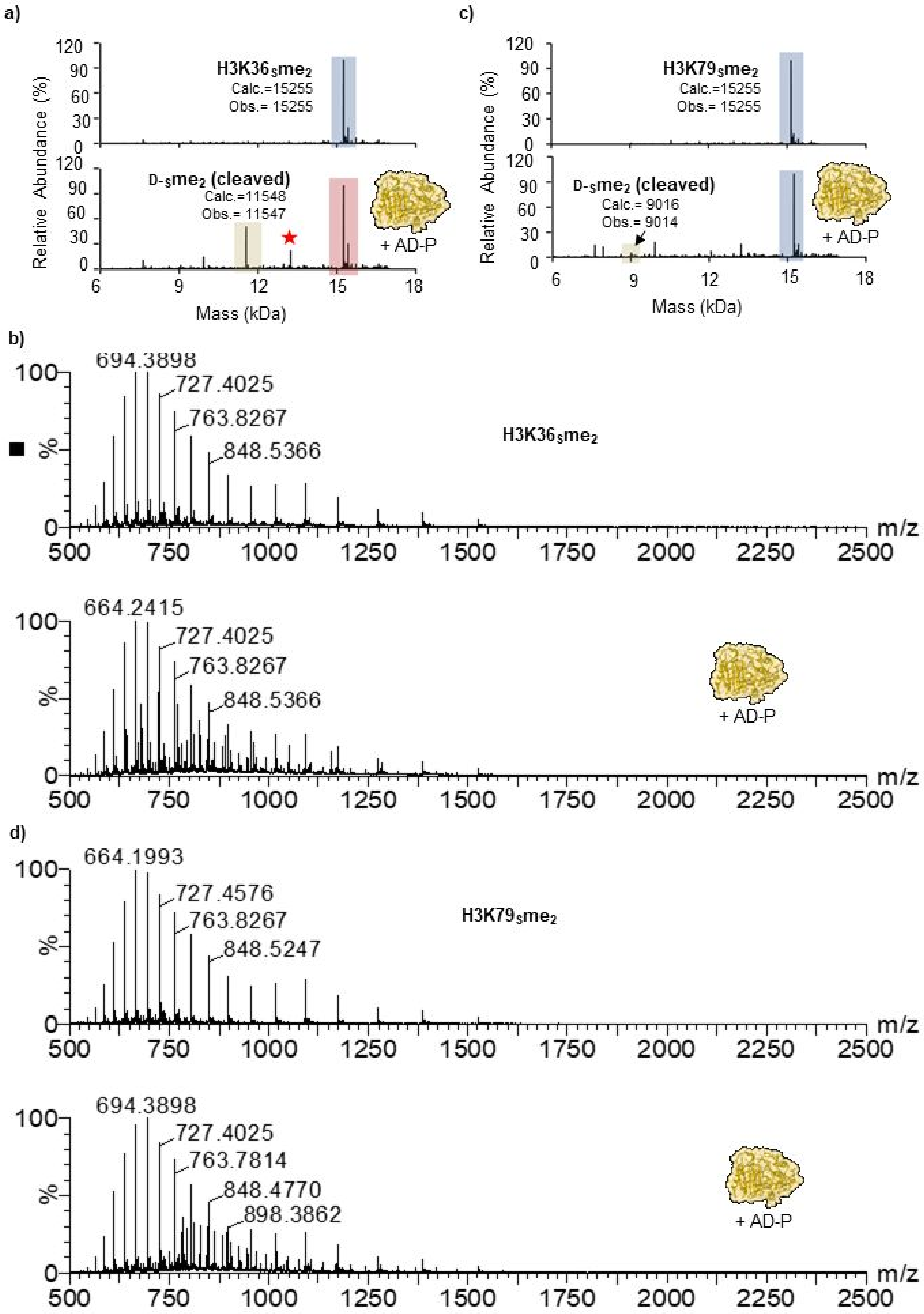
a)
H3K36$_s$me$_2$
Calc.=15255
Obs.= 15255
D-$_s$me$_2$ (cleaved)
Calc.=11548
Obs.= 11547
+ AD-P
Relative Abundance (%)
Mass (kDa)
c)
H3K79$_s$me$_2$
Calc.=15255
Obs.= 15255
D-$_s$me$_2$ (cleaved)
Calc.= 9016
Obs.= 9014
+ AD-P
Relative Abundance (%)
Mass (kDa)
b)
694.3898
727.4025
763.8267
848.5366
H3K36$_s$me$_2$
664.2415
727.4025
763.8267
848.5366
+ AD-P
d)
664.1993
727.4576
763.8267
848.5247
H3K79$_s$me$_2$
694.3898
727.4025
763.7814
848.4770
898.3862
+ AD-P
%
m/z

Fig. S18: Cleavage of dimethyl-lysine analog H3 variants (K36$_S$me$_2$ and K79$_S$me$_2$) by AD-P.

(a,b) Mass spectrum of AD-P cleavage of H3K27$_S$me$_2$. Control (top): calculated mass = 15255 Da, observed mass = 15255 Da. C-terminal cleaved product (bottom): calculated mass = 11548 Da, observed mass = 11547 Da. (c,d) Mass spectrum of AD-P cleavage of H3K9$_S$me$_2$. Control (top): calculated mass = 15255 Da, observed mass = 15255 Da. N-terminal cleaved product (bottom): calculated mass = 9016 Da, observed mass = 9014 Da. The red star indicates a peak originating from AD-P. Colored bars correspond to epimeric starting material before AD-P treatment (blue), C-terminal cleaved product (beige) and uncleaved species after kinetic resolution (red).

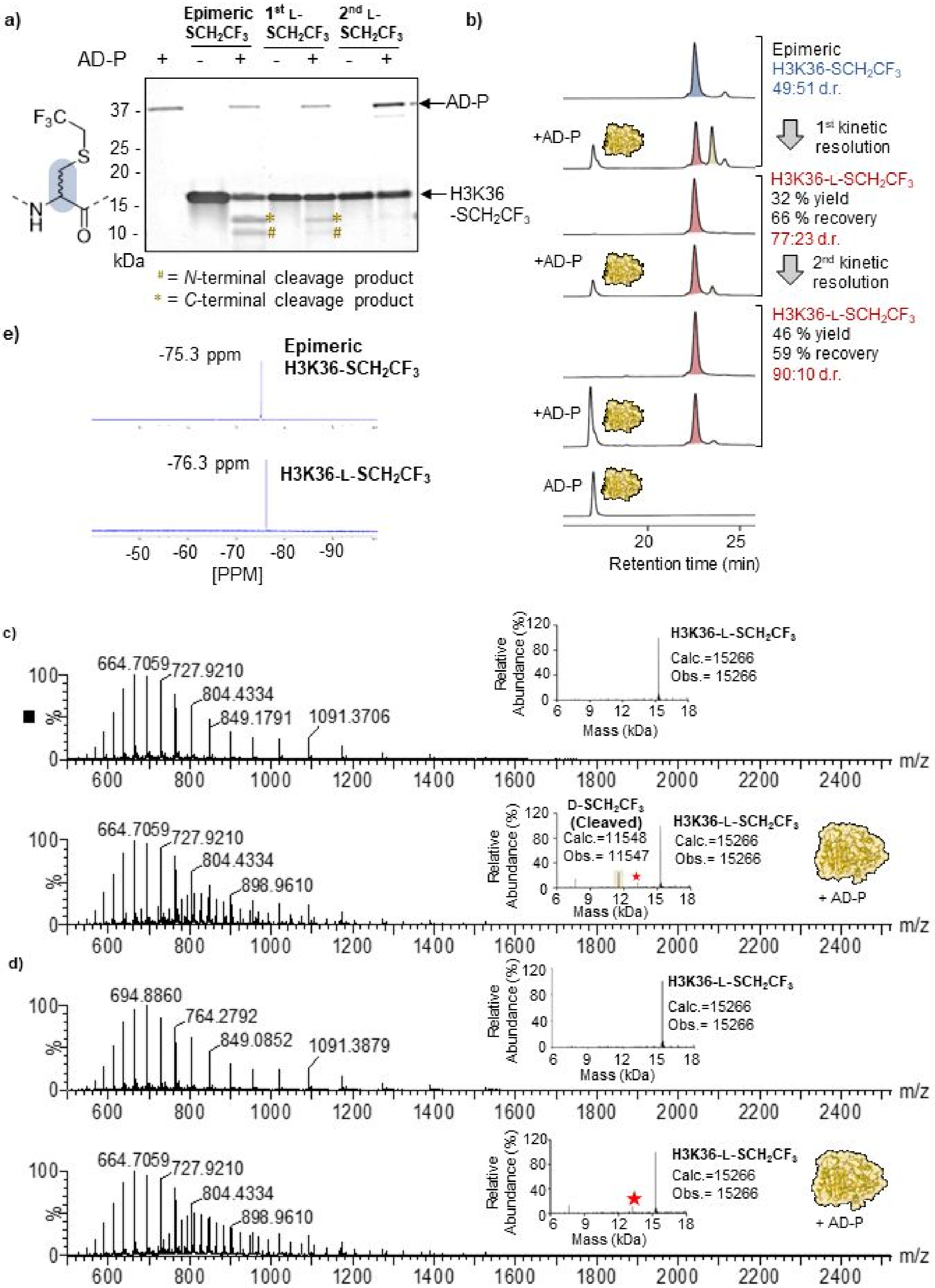

Fig. S19: Stereoselective preparation of H3K36-*L*-SCH$_2$CF$_3$.

(a) Stereoselective preparation of H3K36-L-SCH$_2$CF$_3$ analysed by SDS-PAGE. N- and C-terminal cleavage products are indicated by brown hashes and asterisks, respectively. (b) Stereoselective

preparation of H3K36-L-SCH_2CF_3 analysed by RP-HPLC. At each stage of the process (after 0, 1 or 2 kinetic resolution steps), purified H3K36-SCH_2CF_3 is shown on top and (re)cleavage with AD-P to evaluate stereochemical purity is shown below. Epimeric mixtures are indicated in blue, enriched H3K36-L-SCH_2CF_3 in red, and cleaved D-isomer in beige. (c) Mass spectrum of AD-P cleavage (after 1 kinetic resolution step) on H3K36-L-SCH_2CF_3. Control (top): calculated mass = 15266 Da, observed mass = 15266 Da. C-terminal cleaved product (bottom, beige): calculated mass = 11548 Da, observed mass = 11547 Da. (d) Mass spectrum of AD-P cleavage (after 2 kinetic resolution steps) on H3K36-L-SCH_2CF_3. Control (top): calculated mass = 15266 Da, observed mass = 15266 Da. C-terminal cleaved product (bottom): calculated mass = 11548 Da (not observed). The red star indicates a peak originating from AD-P. C-terminal cleaved product is indicated with beige bar. (e) ^{19}F-NMR analysis of H3K36-SCH_2CF_3 – before AD-P cleavage (top), after 2 steps of kinetic resolution (bottom).

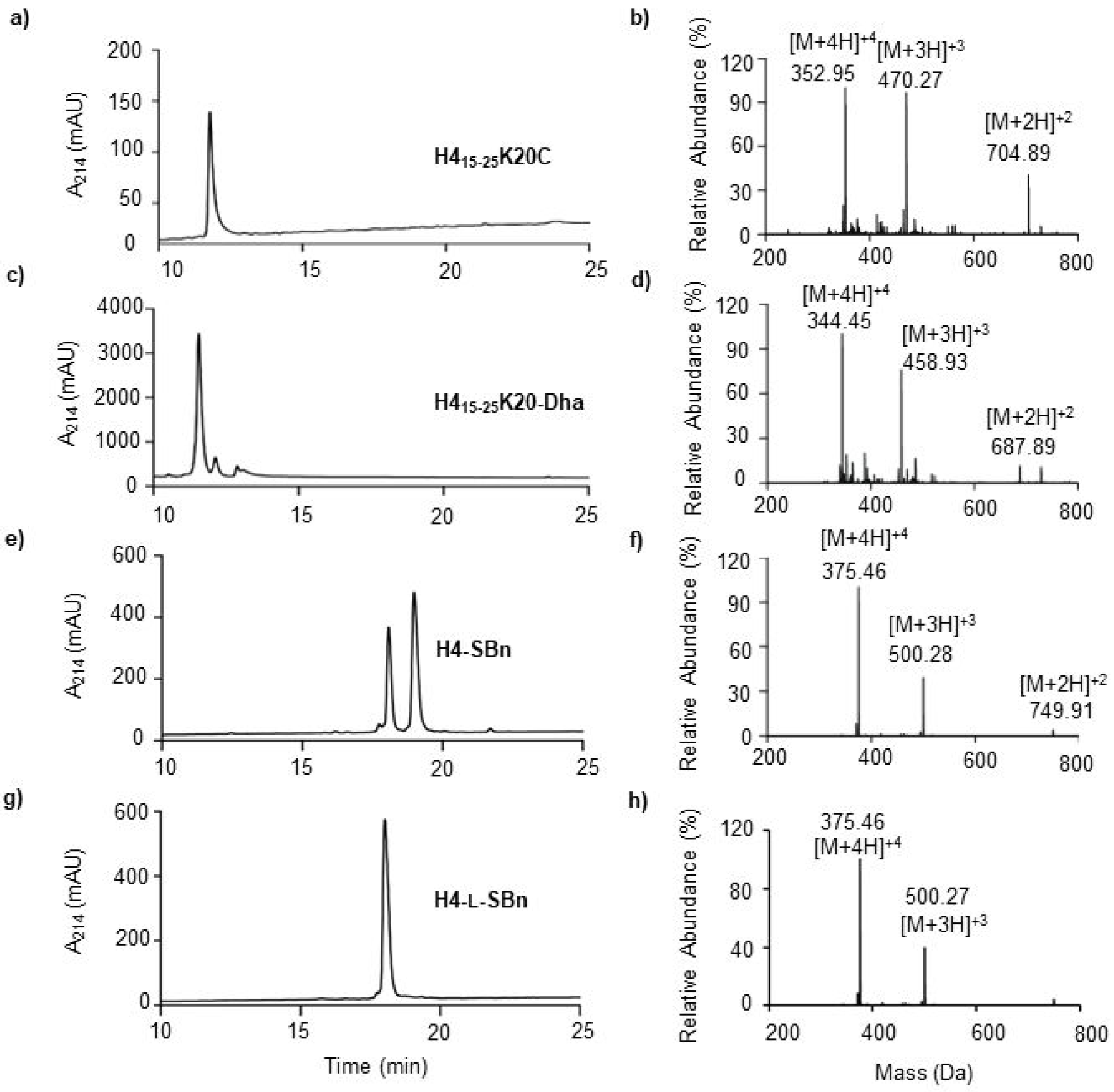

Fig. S20: Chemical modification process of $H4_{15\text{-}25}K20C$ to form epimeric H4-SBn.

(a) RP-HPLC analysis of $H4_{15\text{-}25}K20C$ derived peptide crude, with the expected peak retention time at 11.8 min. (b) MS analysis of crude $H4_{15\text{-}25}K20C$ (calculated mass = 1407.76 Da, observed mass = 1407.78 Da). (c) RP-HPLC analysis of H4-Dha, with the expected peak retention time at 11.6 min. (d) MS analysis of crude H4-Dha (calculated mass = 1373.78 Da, observed mass = 1373.77 Da). (e) RP-HPLC analysis of epimeric H4-SBn, with expected peak retention times at 17.9 and 18.8 min. (f) MS analysis of purified H4-SBn (calculated mass = 1497.81 Da, observed mass = 1497.82 Da). (g) RP-HPLC analysis of H4-L-SBn, with the expected peak retention time at 17.9 min. (h) MS analysis of purified H4-L-SBn (calculated mass = 1497.81 Da, observed mass = 1497.81 Da).

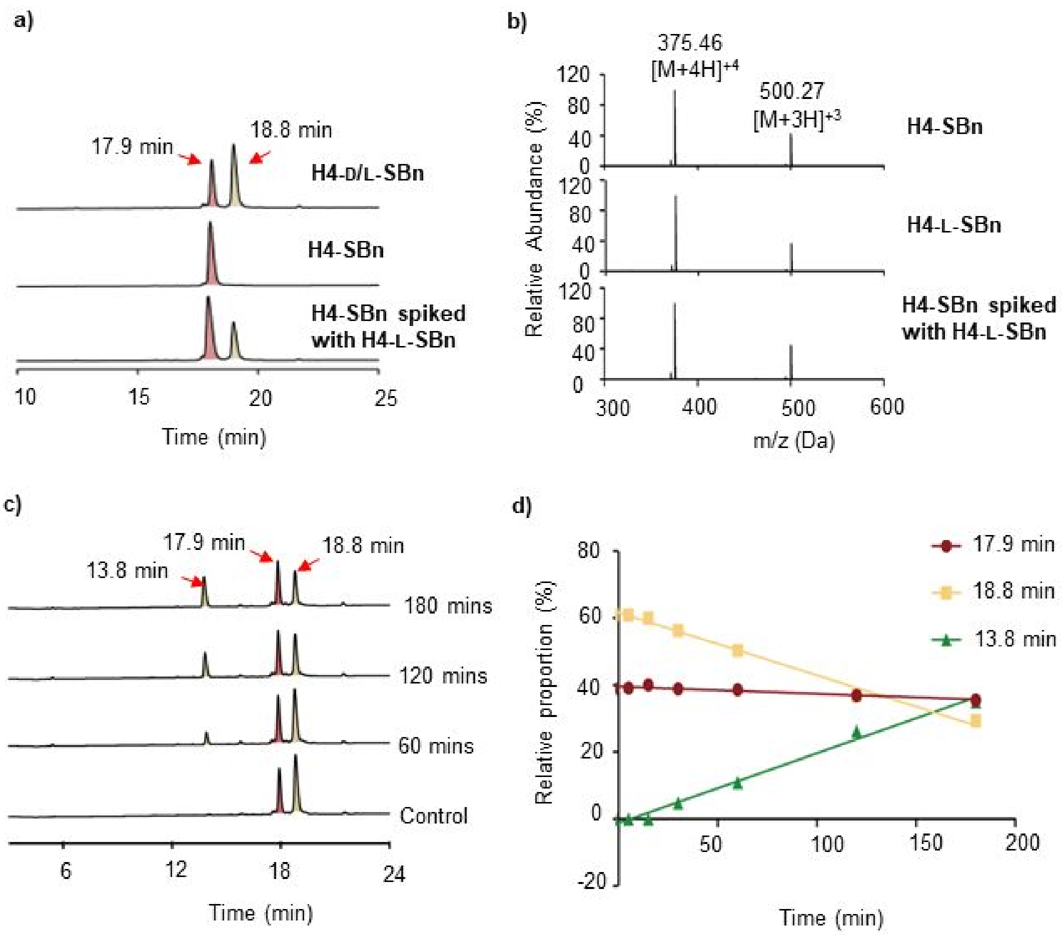

Fig. S21: Confirmation of D-stereoselectivity in AD-P cleavage.

(a) Assignment of H4-SBn epimers by HPLC. Top trace: mixture of peptide isomers containing both L- and D-SBn at position 20. Middle trace: reference HPLC trace of an all-L-isomer sample, synthesised by reacting $H4_{15\text{-}25}$K20C with BnBr. Bottom trace: epimeric mixture (top trace) spiked with the reference all-L-isomer (middle trace), revealing that the earlier-eluting peak (here at 17.9 min RT) corresponds to the L-isomer, and the later-eluting peak (here at 18.8 min RT) represents the D-isomer. (b) Mass spectrometry analysis of spike-in samples: For both L- and D-isomers, calculated mass = 1497.81 Da, observed mass = 1497.82 Da. (c) Time course of the H4-SBn peptide cleavage reaction monitored by RP-HPLC. (d) Quantification of the time course shown in (c). Relative proportions of each species (L, D, and cleaved) were determined by calculating peptide concentrations from HPLC peak areas, and dividing the concentration of each species by the sum of the total concentrations. Rates (cleavage of H4-L-SBn: < 0.01 h^{-1}; cleavage of H4-D-SBn: 0.67 ± 0.02 h^{-1}; formation of $H4_{15\text{-}20}$K20-D-SBn: 0.67 ± 0.02 h^{-1}) were calculated from the slopes of linear fits for each species (best fit value ± error of the fit).

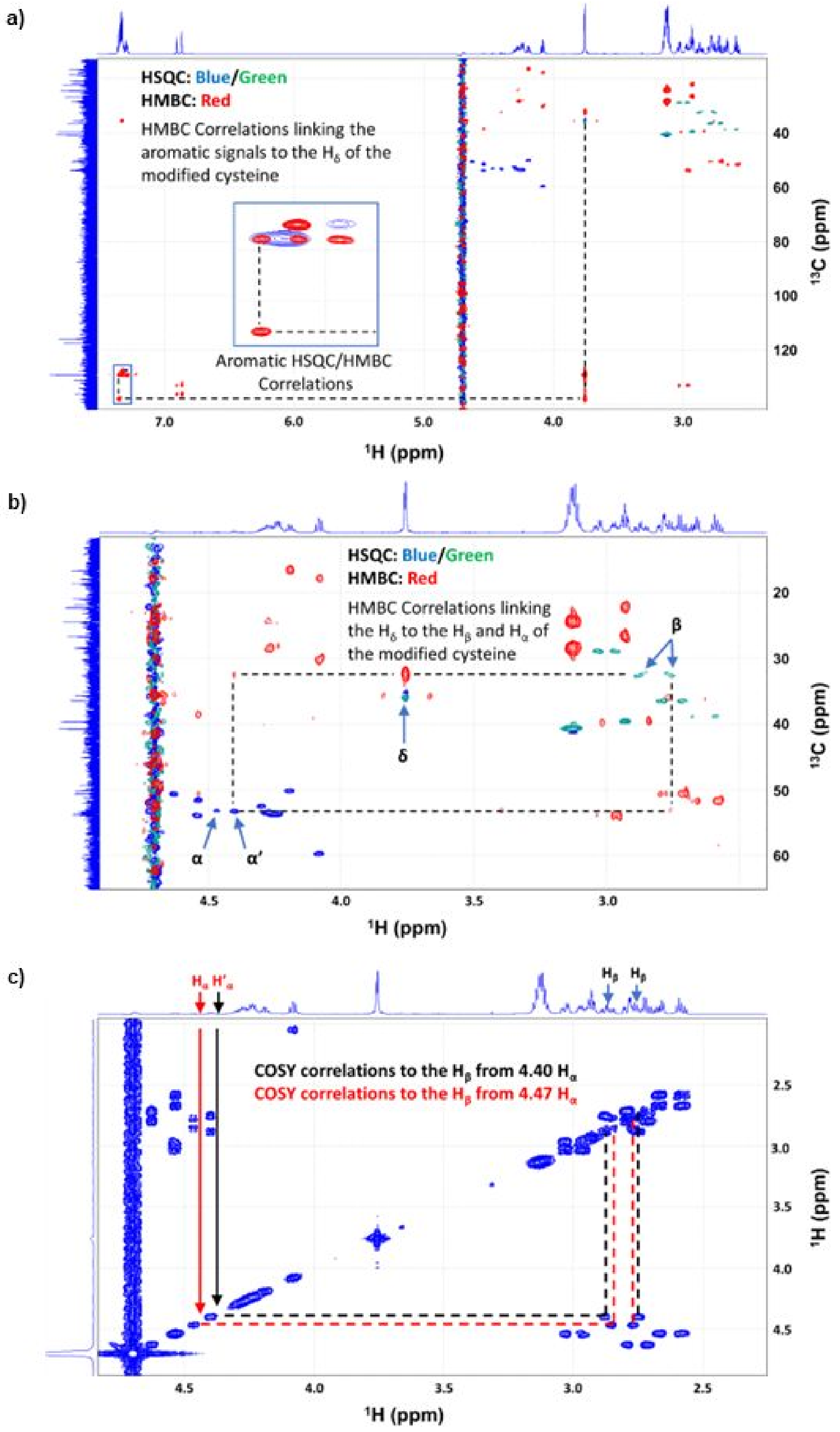

Fig. S22: NMR assignment of diastereotopic peptide peaks.

(a) HSQC (blue/green) and HMBC (red) correlations (dashed lines) connecting the aromatic benzyl signals (7.33 ppm) to the diastereotopic H_δ signals (3.75 and 3.76 ppm) for the modified Cys residue. The inset shows a zoom-in of the boxed aromatic region from the hsqcetgpsp.3 experiment with contour levels adjusted for clarity. (b) HSQC (blue/green) and HMBC (red) correlations (dashed lines)

connecting the $H_δ$ signals (ca. 3.76 ppm) to the prochiral $H_β$ (2.87 ppm and 2.76 ppm) and diastereotopic $H_α$ (4.40 ppm and 4.45 ppm) signals. Only the HMBC correlations for the $H_α$ signal at 4.40 ppm can be seen at the displayed contour levels. (c) COSY correlations for the signals at 4.45 ppm (red lines) and 4.40 ppm (black lines) showing coupling to the modified Cys pro-chiral $H_β$ signals, confirming that they are the diastereotopic $H_α$ signals of the modified Cys residue.

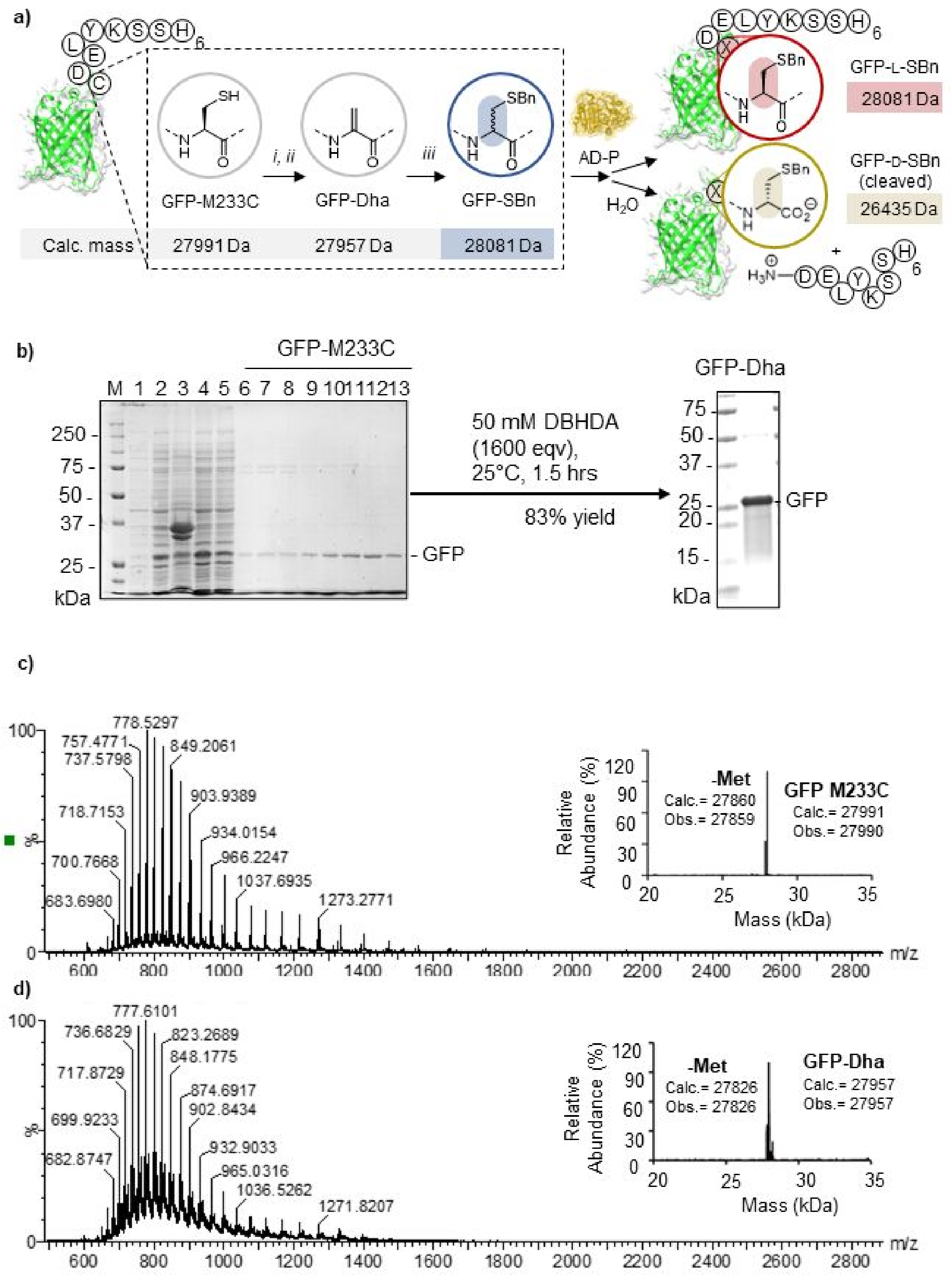

a)
GFP-M233C
GFP-Dha
GFP-SBn
i, ii
iii
AD-P
H2O
Calc. mass
27991 Da
27957 Da
28081 Da
GFP-L-SBn
28081 Da
GFP-D-SBn (cleaved)
26435 Da
b)
GFP-M233C
M 1 2 3 4 5 6 7 8 9 10 11 12 13
250
75
50
37
25
kDa
GFP
50 mM DBHDA (1600 eqv), 25°C, 1.5 hrs
83% yield
GFP-Dha
75
50
37
25
20
15
kDa
GFP
c)
778.5297
757.4771
737.5798
849.2061
903.9389
718.7153
934.0154
966.2247
700.7668
1037.6935
683.6980
1273.2771
m/z
-Met
Calc.= 27860
Obs.= 27859
GFP M233C
Calc.= 27991
Obs.= 27990
Relative Abundance (%)
Mass (kDa)
d)
777.6101
736.6829
823.2689
848.1775
717.8729
874.6917
902.8434
699.9233
932.9033
682.8747
965.0316
1036.5262
1271.8207
m/z
-Met
Calc.= 27826
Obs.= 27826
GFP-Dha
Calc.= 27957
Obs.= 27957
Relative Abundance (%)
Mass (kDa)

Fig. S23: Purification of GFP-M233C and installation of Dha.

(a) Schematic representation of the chemical conversion of GFP-M233C to GFP-SBn. Reagents: i: TCEP; ii: DBHDA; iii: Benzyl mercaptan. (b) (left) SDS-PAGE analysis of GFP-M233C purification via Ni-NTA chromatography: Lane M - Protein marker; Lane 1 - Cell lysate (before IPTG induction); Lane 2 - Cell lysate (after IPTG induction); Lane 3 - Pellet fraction; Lane 4 - Supernatant fraction; Lane 5 - Flow-through fraction; Lanes 6-10 - Elutions containing 70 mM imidazole, Lanes 10-13 - Elutions containing 150 mM imidazole; (right) SDS-PAGE analysis of GFP-M233Dha final product (c) Mass spectrum of GFP-M233C (calculated mass = 27991 Da (27860 Da -Met); observed mass = 27990 Da (27859 Da -Met)). (d) Mass spectrum of GFP-M233-Dha (calculated mass = 27957 Da (27826 Da -Met); observed mass = 27957 Da (27826 Da -Met).

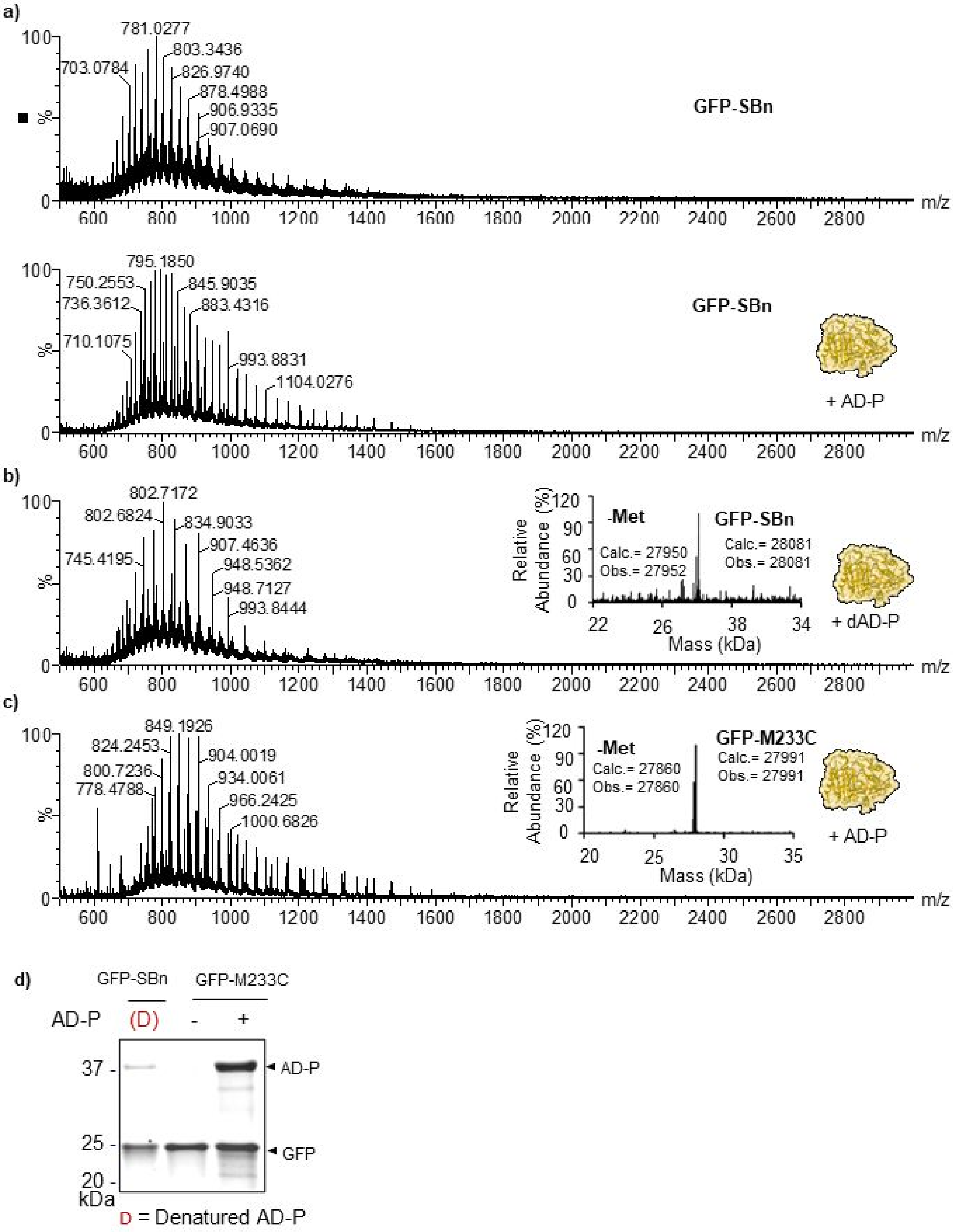
a)
781.0277
803.3436
703.0784
826.9740
878.4988
906.9335
907.0690
GFP-SBn
795.1850
750.2553
845.9035
736.3612
883.4316
710.1075
993.8831
1104.0276
GFP-SBn
+ AD-P
b)
802.7172
802.6824
834.9033
907.4636
745.4195
948.5362
948.7127
993.8444
-Met
Calc.= 27950
Obs.= 27952
GFP-SBn
Calc.= 28081
Obs.= 28081
Relative Abundance (%)
Mass (kDa)
+ dAD-P
c)
849.1926
824.2453
904.0019
800.7236
934.0061
778.4788
966.2425
1000.6826
-Met
Calc.= 27860
Obs.= 27860
GFP-M233C
Calc.= 27991
Obs.= 27991
Relative Abundance (%)
Mass (kDa)
+ AD-P
m/z
d)
GFP-SBn
GFP-M233C
AD-P
(D)
-
+
37 -
25 -
20 -
kDa
AD-P
GFP
D = Denatured AD-P

Fig. S24: Incorporation of -SBn modification on GFP-M233C and subsequent cleavage by AD-P.

(a) Mass spectrum of AD-P cleavage on GFP-SBn. Control (top), calculated mass = 28081 Da (27950 Da -Met); observed mass = 28081 Da. Cleavage reaction (bottom), *N*-terminal cleaved product: calculated mass = 26435 Da (26304 Da -Met); observed mass = 26435 Da (26304 Da -Met). (b) Mass spectrum of GFP-SBn and denatured (D) AD-P reaction (calculated mass = 28081 Da (27950 Da -Met); observed mass = 28081 Da (27952 Da -Met) (c) Mass spectrum of GFP-M233C and AD-P reaction (calculated mass = 27991 Da (27860 Da -Met); observed mass = 27991 Da (27860 Da -Met)). (d) SDS-PAGE analysis of negative controls of GFP-SBn cleavage by AD-P. Denatured AD-P is indicated as (D).

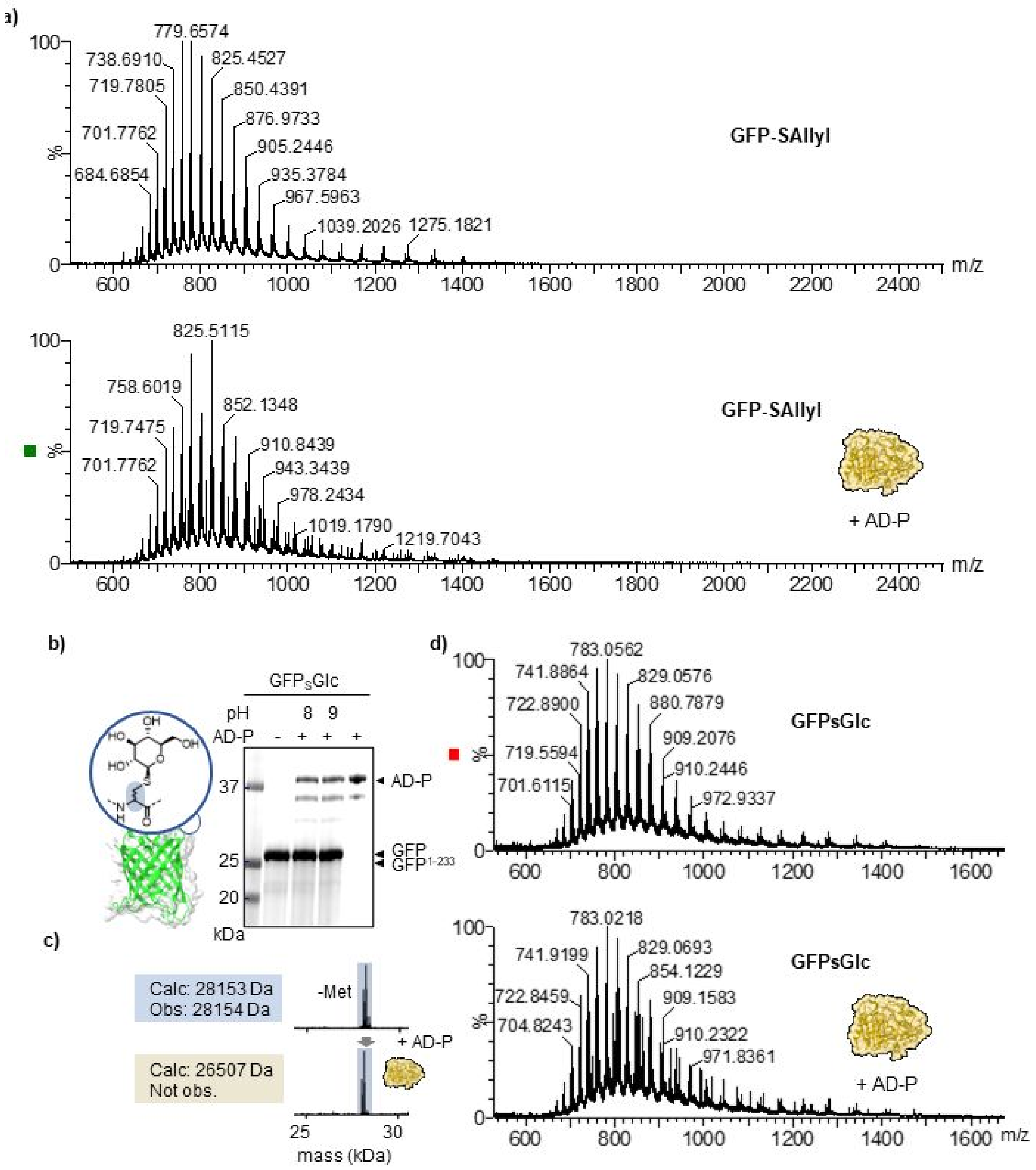

Fig. S25: Cleavage of Sallyl and $_{S}$Glc modifications on GFP by AD-P.

(a) Mass spectrum of AD-P cleavage on GFP-SAllyl. Control (top), calculated mass = 28031 Da (27900 Da -Met); observed mass = 28032 Da (27901 Da -Met). Cleavage reaction (bottom), *N*-terminal cleaved product: calculated mass = 26435 Da (26304 Da -Met); observed mass = 26435 Da (26304 Da -Met). (b) SDS-PAGE analysis of attempted GFP$_{S}$Glc cleavage by AD-P. (c,d) Mass spectrum of AD-P cleavage on GFP$_{S}$Glc. Control (top), calculated mass = 28153 Da (28022 Da -Met); observed mass = 28154 Da (28022 Da -Met). Cleavage reaction (bottom), *N*-terminal cleaved

product: calculated mass = 26507 Da (not observed). Colored bars correspond to epimeric starting material before AD-P treatment (blue).

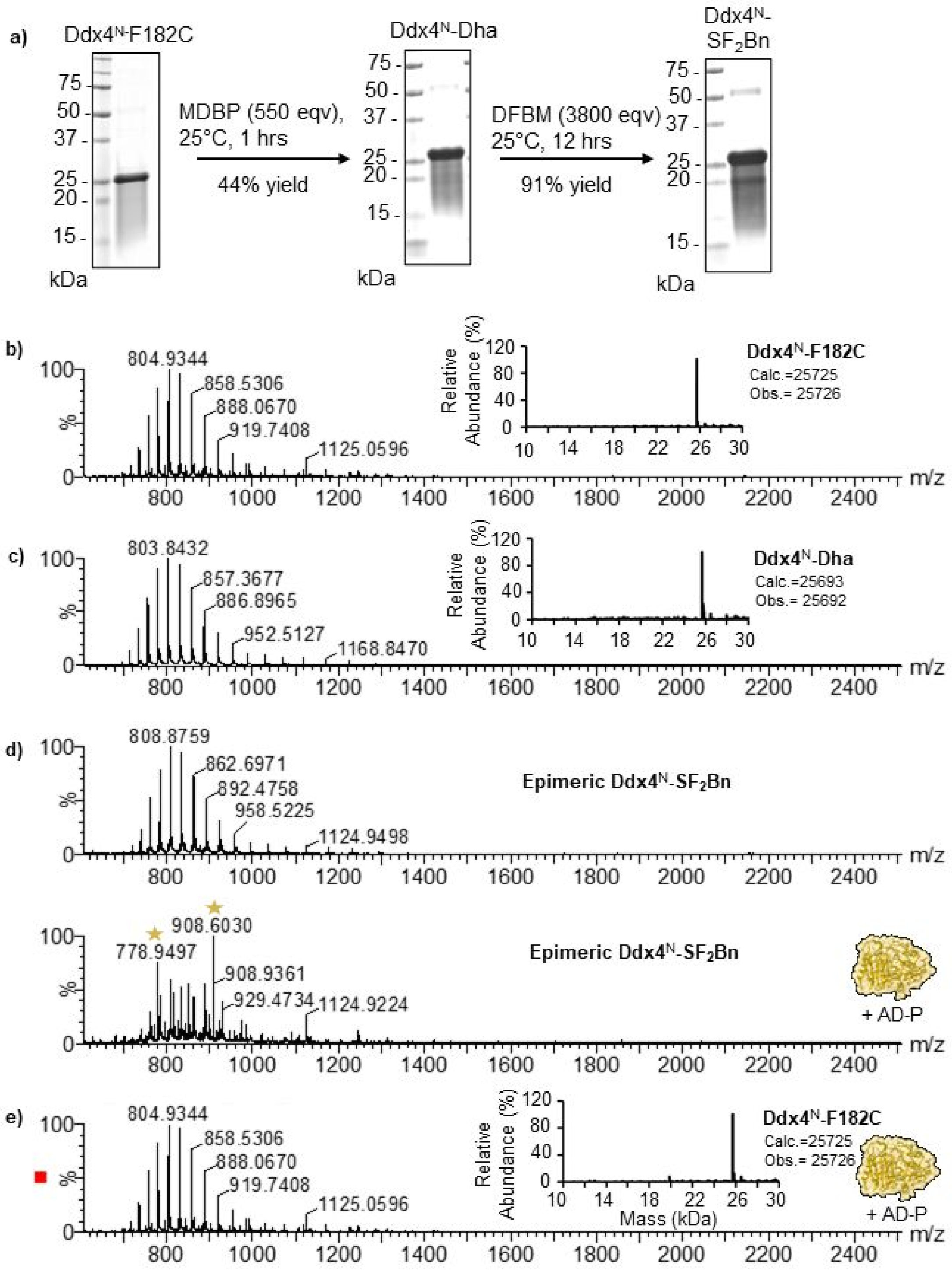

Fig. S26: Incorporation of SF_2Bn modification on $Ddx4^N$ and subsequent cleavage by AD-P.

(a) SDS-PAGE analysis of $Ddx4^N$-F182C purification (left), $Ddx4^N$-Dha (middle) and $Ddx4^N$-SF_2Bn (right). (b) Mass spectrum of $Ddx4^N$- F182C (calculated mass = 25725 Da; observed mass = 25726 Da. (c) Mass spectrum of $Ddx4^N$-Dha (calculated mass = 25693 Da; observed mass = 25692 Da.
(d) Mass spectrum of AD-P cleavage on $Ddx4^N$-SF_2Bn. Top: control, calculated mass = 25853 Da; observed mass = 25852 Da. Bottom: cleavage reaction, for the *N*-terminal cleaved product, calculated mass for cleaved D-isomer = 20424 Da; observed mass = 20424 Da. The *C*-terminal cleaved product, calculated mass for cleaved D-isomer = 5442.6 Da; observed mass = 5442.6 D. (e) Mass spectrum of AD-P cleavage on of $Ddx4^N$-F182C, calculated mass = 25725 Da; observed mass = 25726 Da. Beige stars correspond to the *C*-terminal cleavage product in (d).

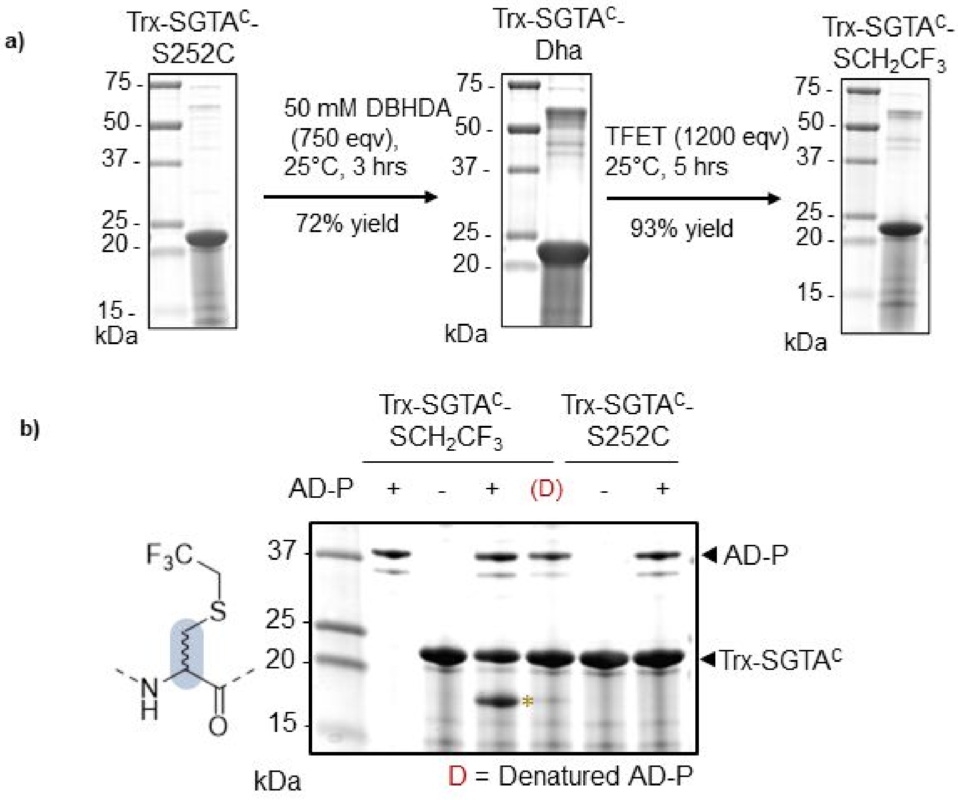

Fig. S27: Incorporation of -SCH_2CF_3 modification on Trx-SGTA^C and subsequent cleavage by AD-P.

(a) SDS-PAGE analysis of Trx-SGTA^C-S252C purification (left), Trx-SGTA^C-Dha (middle) and Trx-SGTA^C -SCH_2CF_3 (right). (b) SDS-PAGE analysis of Trx-SGTA^C-SCH_2CF_3 cleavage by AD-P. The *N*-terminal cleavage product is marked with a beige asterisk. Denatured AD-P is indicated with a red (D).

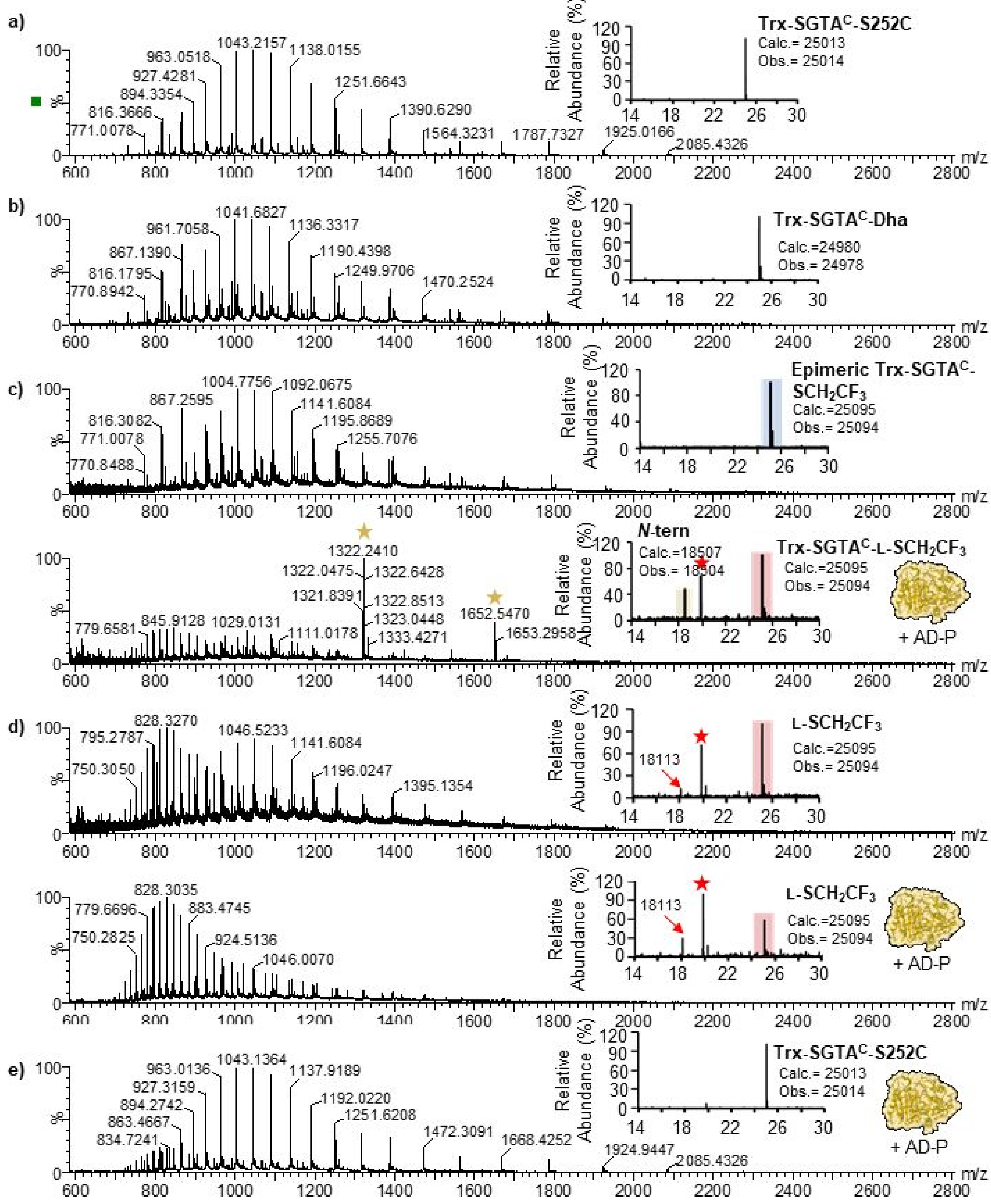

Fig. S28: Incorporation of -SCH_2CF_3 PTM on Trx-SGTA^C protein and Stereoselective preparation of Trx-SGTA^C-*L*-SCH_2CF_3.

(a) Mass spectrum of Trx-SGTA^C-252C (calculated mass = 25013 Da; observed mass = 25014 Da.
(b) Mass spectrum of Trx-SGTA^C-Dha (calculated mass = 24980 Da; observed mass = 24978 Da.
(c) Mass spectrum of AD-P cleavage on Trx-SGTA^C-SCH_2CF_3. Control (top), calculated mass = 25095 Da; observed mass = 25094 Da. Cleavage reaction (bottom), *N*-terminal cleaved product: calculated mass = 18507 Da; observed mass = 18504 Da; *C*-terminal cleaved product, calculated

mass = 6603.2 Da; observed mass = 6603.5 Da, The deconvoluted results are repeated here for clarity. (d) Re-cleavage of partially purified Trx-SGTA^{C}-L-SCH_2CF_3. Control (top), calculated mass = 25095 Da; observed mass = 25094 Da. Cleavage reaction (bottom), *N*-terminal cleaved product, calculated mass = 18507 Da; not observed. (e) Mass spectrum of AD-P cleavage of Trx-SGTA^{C}-S252C, calculated mass = 25013 Da; observed mass = 25014 Da. Red stars indicate peaks originating from AD-P. Beige stars correspond to the *C*-terminal cleavage product in (c). Colored bars correspond to epimeric starting material before AD-P treatment (blue), C-terminal cleaved product (beige) and uncleaved species after kinetic resolution (red).

Experimental Procedures

Materials and General Methods

Chemicals used in chemical mutagenesis procedures: Tris(2-Carboxyethyl)phosphine hydrochloride (TCEP), 2,2,2-Trifluoroethanethiol (TFET), Allyl mercaptan, N-Acetylcysteamine, and imidazole were purchased from Merck Sigma-Aldrich. 2,5-Dibromohexanediamide (DBHDA) and benzyl bromide were purchased from Fluorochem. Benzyl mercaptan,2-(dimethylamino) ethanethiol hydrochloride, were purchased from Fisher Scientific. 2-Propanethiol, Thiophenol, was purchased from Thermo Scientific. 3-Chloro-1-propanethiol, 3,5-Difluorophenyl) methanethiol, Cyclopentanethiol, and Methyl 2,5-dibromopentanoate were purchased from Fluorochem, Cyclohexanethiol was purchased from ChemCruz,

Peptide resins, Fmoc-L-amino acids, Oxyma and K-Oxyma, were purchased from Novabiochem. Diisopropylethylamine (DIEA) and piperidine were purchased by Merck Sigma-Aldrich and diisopropylcarbodiimide (DIC) from Merck. Peptide synthesis grade dimethylformamide (DMF) was purchased from Cambridge Reagents Ltd. N-Methyl-2-pyrrolidone (NMP), triisopropylsilane (TIS), thioanisole,1,2-ethanedithiol and dichloromethane (DCM) from Merck. Peptide grade trifluoroacetic acid (TFA) was purchased from FluoroChem. Dithiothreitol (DTT) was purchased from AnaSpec.Inc. Reagents and solvents were used without further purification.

For protein purification, Ni Sepharose 6 FastFlow beads were obtained from GE Healthcare. DNA and native protein concentrations were determined using a NanoDrop ND-8000, 8-sample spectrophotometer (Labtech). Plasmid sequencing was carried out by GENEWIZ. All competent cells were made in-house from commercial strains (TOP10 for cloning, BL21 for expression).

General Procedure for Reverse-Phase Chromatography

Analytical and semi-preparative reverse-phase high-performance liquid chromatography (RP-HPLC) was performed on an Agilent 1260 Infinity II instrument equipped with a dual wavelength UV-VIS detector. For analytical work, a 300SB-C3 4.6x150 mm column and an Eclipse XDB-C18 4.6x150 mm column was used at a constant flow of 1 mL/min at 25 °C. In semi-preparative mode, a 300SB-C3 9.4x250 mm column was used at a flow rate of 3.5 mL/min. HPLC purification of peptides were performed on an Agilent 1260 Preparative HPLC system using a reversed phase Zorbax 300SB-C3 21.2x150 mm 7 µm column running at 20 mL/min.

Typical gradients using mixture of H_2O containing 0.1% TFA (solvent A) and Acetonitrile containing 0.1% TFA (solvent B) are detailed below.

Gradient A: linear gradient from 35-70% B over 30 min; RT

Gradient B: linear gradient from 30-60% B over 30 min; RT

Gradient C: linear gradient from 0-70% B over 30 min; RT

Gradient D: linear gradient from 0-50% B over 30 min; RT

Gradient E: linear gradient from 0-40% B over 30 min; RT

Mass Spectrometry

High-resolution mass spectra (HRMS) were recorded on a Waters Xevo G2-XS QTof after separation by reverse phase chromatography on a Waters Acquity UPLC. The proteins were analysed on a Waters Acquity UPLC Protein BEH C4, 300A, 1.7 um, 2.1 x 50 mm column, and the peptides on a Waters Acquity UPLC BEH C18, 1.7 um, 2.1 x 50 mm column, with a constant flow of 0.2 mL/min gradient of water containing 0.1% formic acid (solvent C) and acetonitrile containing 0.1% formic acid (solvent D). MassLynx V4.2 (Waters Corp.) was used to process the data. Under the chromatographic conditions employed, the protein substrates and cleavage products co-elute. Therefore, the mass spectra are integrated over an entire peak range and the resulting ion series are then deconvoluted using a maximum entropy algorithm. All deconvoluted HRMS data were normalised between 0 and 1. Percentage conversions were derived from the intensity of peaks on the deconvoluted spectrum unless otherwise specified.

Methods

Protein and peptide preparation

Site-directed mutagenesis of GFP, $Ddx4^N$ and H3 proteins

Primers were designed using NEBaseChanger. The PCR reaction mixture was as follows: 1 µL pET15b-GFP-6His, pET-Sumo-2HTb-Ddx4 or pET30-H3 plasmid (diluted to 10 ng/µL), 5 µL Q5 reaction buffer, 10 µM forward and reverse primers (listed in Table S1), 10 mM dNTP, 0.5 µL Q5 DNA polymerase and 15.75 µL H_2O were added (final volume = 25 µL). The mixture was placed in a thermocycler, programmed to 30 sec of initial denaturation at 98 °C, followed by 35 cycles of 10 sec of denaturation at 98 °C, and 20 sec of annealing at the recommended annealing temperature (Ta), and a 3 min extension at 72 °C. After 35 cycles, the mixture was held at 72 °C for 5 min in a final extension. The mixture was incubated overnight at 37 °C with Dpn1. Ligation was carried out by incubation of the following reaction mixture at room temperature for 4 hr: 10 µL PCR product, 2 µL 10x kinase buffer, 2 µL 10x ligase buffer, 2 µL 100 mM ATP, 1 µL 200 mM DTT, 1 µL PNK and 1 µL DNA ligase. TOP10 cells were transformed with 5 µL of the reaction mixture, streaked on an agar plate containing 100 µg/mL ampicillin for pET15b-GFP-6His and 50 µg/mL kanamycin for pET-Sumo-2HTb-Ddx4 & pET30-H3. The plates incubated overnight at 37 °C. Single colonies were expanded overnight at 37 °C in 5 mL LB medium with corresponding antibiotics shaking at 220 rpm. Cells were lysed and the plasmid was purified using a plasmid miniprep kit (Thermo Fisher Scientific). Plasmids were sent to Azenta Life Science (UK) company for sequencing and stored at -20 °C.

AD-P expression and purification

An *Escherichia coli* transformant expressing AD-P was prepared as follows. A gene encoding AD-P[2] was ordered from Azenta Life Sciences (UK) and subcloned into a pET-15b vector in frame with a C-terminal hexahistidine tag (6His). AD-P was expressed and purified as previously described.[2]The

vector expressing AD-P-6His was transformed into an *E. coli* expressing strain BL21 (DE3). A colony containing the AD-P-6His plasmid was subcultured overnight (220 rpm, 37 ºC) in 5 mL LB medium supplemented with 100 µg/mL ampicillin. The starter culture was subsequently used to inoculate 2 L LB medium supplemented with 100 µg/mL ampicillin and the mixture cultured for 3 h (220 rpm, 37 ºC) until an optical density of A_{600} = 0.4 was achieved. After further incubation at 23 ºC for 1 h, protein expression was induced by addition of 0.5 mM IPTG. The culture was shaken overnight at 23 ºC. Cells were harvested by centrifugation (30 min, 4200 rpm, 4 ºC), and subsequently washed and resuspended in buffer A. Cells were disrupted with an ultrasonic oscillator (25 kPsi). The insoluble fraction was removed as a pellet by centrifugation (1 h, 16500 rpm, 4 ºC). Supernatant was collected (and stored at 4 ºC if needed). Cell lysate was loaded onto a 3 mL Ni-NTA gravity column and washed with buffer A (containing 10 mM imidazole). 10 mL buffer A (containing 70 mM imidazole) was added, and fractions were collected. AD-P was eluted and collected with 10 mL buffer A (containing 150 mM imidazole). The presence of AD-P in eluted fractions was confirmed by SDS-PAGE. Purest AD-P fractions were collected and underwent buffer exchange into buffer B (0.1 M HEPES pH 7.6, 100 mM NaCl, 10% Glycerol). Aliquots were flash frozen and stored at -80 ºC. The final protein purity and mass were confirmed by SDS page analysis **(Figure S3a)** and mass spectrometry (**Figure S3b**). Aliquots were flash frozen and stored at -80 ºC.
AD-P Buffer A = 20 mM Sodium phosphate (pH 8.0), 100 mM NaCl,

H3 protein expression and purification

H3 cysteine mutants (K9C, K27C, K36C and K79C) were expressed and purified from inclusion bodies as previously described with some minor modifications.[3] Expression plasmids transformed into BL21, and 500 mL (50 µg/mL kanamycin) culture were grown to OD_{590} = 0.6 at 37 ºC and expression was induced with the addition of 0.5 mM IPTG for 3 hours. The inclusion body was prepared by lysing cells in lysis buffer (50 mM Tris pH 7.6, 100 mM NaCl, 1 mM EDTA, 1 mM DTT), followed by centrifuging at 4 ºC for 30 min at 15,000 rpm. The supernatant was decanted, and the pellet resuspended in lysis buffer + 1% Triton X and spun again at 15,000 rpm 4ºC for 15 min. The pellet was washed once more in lysis buffer without Triton and spun 15,000 rpm at 4 ºC for 15 min. The inclusion body pellet was then resuspended in buffer containing 6 M guanidine, 20 mM Tris, 1 mM EDTA, 100 mM NaCl, 1 mM DTT pH 8 and left to mix in the cold room for 2 hr before being centrifuged for 30 min 15,000 rpm. The supernatant was collected, acidified with TFA, filtered, and injected onto analytical and preparative HPLC systems. Analytical data:
K9C: PR-HPLC gradient 35-70% B, r_t = 22.4 min. Calculated mass = 15184, observed mass = 15183 Da (**Figure S1).**
K27C: PR-HPLC gradient 30-60% B, r_t = 29.2 min. Calculated mass = 15184, observed mass = 15184 Da (**Figure S31a, b).**
K36C: PR-HPLC gradient 30-60% B, r_t = 29.2 min. Calculated mass = 15184, observed mass = 15184 Da (**Figure S32a, b).**
K79C: Calculated mass = 15184, observed mass = 15184 Da (**Figure S33a).**

GFP expression and purification

GFP cysteine mutant (GFP-M233C) was expressed and purified as previously described.[4] The vector expressing GFP was transformed into *E. coli* expressing strain BL21 (DE3). A colony containing the GFP plasmid were subcultured overnight (220 rpm, 37 ºC) in 5 mL LB medium supplemented with 100 µg/mL ampicillin. The starter culture was subsequently used to inoculate 1 L LB medium supplemented with 100 µg/mL ampicillin and the suspension cultured for 4 h (220 rpm, 37 ºC) until an optical density of A_{600} = 0.4 was achieved. Protein expression was induced by addition of 0.5 mM IPTG. The culture was shaken overnight at 25 ºC (220 rpm). Cells were harvested by centrifugation (30 min, 4200 rpm, 4 ºC), and subsequently washed and resuspended in buffer A. After cells were disrupted, the lysate was centrifuged (40 min, 13000 rpm, 4 ºC) to pellet any insoluble cell debris. Supernatant was collected and applied to 3 mL Ni-NTA gravity column and washed with buffer A (containing 10 mM imidazole). 10 mL buffer A (containing 70 mM imidazole) was added, and fractions were collected. GFP was eluted and collected with 10 mL buffer A (containing 150 mM imidazole). The presence of GFP in eluted fractions was confirmed by SDS-PAGE (**Figure S23b left**). Purest GFP fractions were combined, and mass was confirmed by mass spectrometry (**Figure S23c**). Aliquots were flash frozen and stored at -80 ºC.
GFP Buffer A = 10 mM Tris (pH 8.0), 150 mM NaCl, 1 mM PMSF, 0.5 mM DTT.

$Ddx4^N$-F182C expression and purification

A single Cys residue was introduced into the intrinsically disordered region (residues 1-236) of the DEAD-Box Helicase 4 F182C ($Ddx4^N$-F182C) by site-directed mutagenesis using a Cys-free version of the pET-His-SUMO-$Ddx4^N$ expression plasmid described previously.[5] Plasmids were transformed into calcium competent BL21 *E. coli* cells made in-house. Transformants were grown in Luria broth with 50 µg/mL kanamycin at 37°C, shaking at 220 RPM, overnight. The preculture was diluted in 1-in-100 into Luria broth with 50 µg/mL kanamycin and further growth was carried out at 37°C, shaking at 220 RPM until reaching an OD_{600} between 0.6-0.8. Protein expression was then induced with 0.4 mM IPTG at 18°C, shaking at 180 RPM, overnight. Harvested cells were washed with PBS and resuspended in 6 M GdnHCl, 50 mM Tris pH 8.0, 500 mM NaCl, 2 mM TCEP and incubated for 30 minutes, on ice. Cells were passed through a cell-disruptor three times and centrifuged at 18,000 RPM, at 4°C for 30 minutes. The resulting supernatant was loaded onto 4 mL Protino™ Ni-NTA agarose (Macherey-Nagel Bioanalysis™) and incubated at 4°C, rotating for 2 hours. Flowthrough was discarded, and bound protein was washed with 50 mM Tris pH 8.0, 500 mM NaCl, 20 mM imidazole, 2 mM TCEP. 2 mg of His-ULP1 (made in-house) was added to the resin and incubated at 4°C, rotating overnight. Cleaved protein was collected in eluent, concentrated to under < 5 mL using Vivaspin 10 kDa MWCO centrifugal concentrator (Sartorius), and buffer exchanged into 20 mM NaPi, 500 mM NaCl, 2 mM TCEP, pH 7.5 by size exclusion chromatography using the HiLoad® 16/600 Superdex® 75 prep grade (Cytiva) at 4°C. Fractions of pure protein were pooled, aliquoted into 2 mL fractions and stored at -80°C in 10% glycerol. The sample was then analysed by SDS-PAGE **(Figure S26a left)** and HRMS **(Figure S26b)**. Analytical data: Calculated mass $Ddx4^N$-F182C = 25725 Da; observed mass = 25726 Da**.**

Trx-SGTA^C protein expression and purification

Chemically competent *E. coli* T7 Express cells (New England Biolabs) were transformed with a pET-28a (+) vector containing thioredoxin-tagged Trx-SGTA^C S252C (residues 213-313) and the gene for kanamycin resistance, and then plated on antibiotic-selective LB agar plates. Single colonies of transformed cells were used to inoculate 10 mL LB + 50 µg/mL kanamycin starter cultures and grown shaking at 220 rpm at 37 °C for 16 hours. The starter cultures were then used to inoculate 0.5 L LB + 50 µg/mL kanamycin expression cultures which were grown at 37°C until OD_{600} = 0.6 - 0.8 before being induced with 0.5 mM Isopropyl ß-D-1-thiogalactopyranoside (IPTG) and the temperature reduced to 18 °C for 18 - 20 hours. Cells were then pelleted at 4200 RPM for 40 minutes, resuspended in lysis buffer (20 mM potassium phosphate (pH 8), 300 mM NaCl, 10 mM imidazole, 0.5 mM DTT, 0.1mg/mL DNase I, one cOmplete EDTA-free protease inhibitor cocktail tablet per 50 mL buffer) and homogenised by a single pass through a One Shot Cell Disruptor at 25 KPSi. Homogenised cells were then ultracentrifuged at 32,000 RPM for 45 minutes to pellet cell debris. The soluble fraction was applied via a 50 mL Superloop to a Cytiva HisTrapFF 5 mL pre-equilibrated with HisTrap Buffer A (20 mM potassium phosphate (pH 8), 300 mM NaCl, 10 mM imidazole, 0.5 mM DTT). Protein was eluted by HisTrap Buffer B (20 mM potassium phosphate (pH 8), 300 mM NaCl, 300 mM imidazole, 0.5 mM DTT) and collected in 2 mL fractions. Fractions were analysed for protein content by SDS-PAGE then pooled and concentrated using a 3 kDa MWCO centrifugal concentrator unit. Once concentrated to < 2.5 mL, protein was loaded onto a 120 mL Superdex 75 gel filtration column (Cytiva) and eluted by Size Exclusion Buffer (10 mM potassium phosphate (pH 8), 100 mM NaCl, 0.5 mM DTT). Eluate was collected in 2 mL fractions and concentrated according to downstream requirements. Final protein concentration was calculated by measuring A280 and dividing by the extinction coefficient for Trx-SGTA (15470.00 $M^{-1}cm^{-1}$). The sample was then analysed by SDS-PAGE **(Figure S27a left)** and HRMS **(Figure S28a)**. Analytical data: Calculated mass Trx-SGTA^C S252C = 25013 Da; observed mass = 25014 Da.

Peptide synthesis and purification0020

H4K20C$_{15-25}$ and OrexinB analog peptide were prepared using automated solid-phase peptide synthesis on a Biotage Alstra Initiator+. Rink amide resin (0.222 g) was washed and swelled with 10 mL of DMF for 20 min (70 °C) in a reaction vessel. The N-terminal Fmoc group was removed with 20% (v/v) piperidine in DMF reacting at room temperature for 10 min. Coupling took place with Fmoc-AAs (5 eq), DIC/Oxyma (5 eq) for 5 min at 75 °C. The above deprotection, coupling steps were repeated to align all other amino acids in the correct sequence. After the completion of coupling, beads were treated with 10 mL capping solution (DMF/Acetic anhydride) for 10 min at room temperature to block the unreacted amine at *N*-terminus of peptide (OrexinB analog peptide excluded). Side chain protection groups of the peptide were removed with concomitant cleavage from the resin using a cleavage cocktail (95% TFA, 2.5% deionized water, 2.5% triisopropyl silane) for 1.5

hr at room temperature. The solution was separated from the resin through filtration. The filtered peptide solution was precipitated by 20 mL of cold ether, centrifuged at 14,000 g for 10 min, same precipitation method was repeated three times, followed by allowing the pellet to dry at room temperature.

The dried pellet was dissolved in 5 mL 50% acetonitrile and purified by HPLC using the following methods:

For peptide $H4_{15\text{-}25}$ K20C, the crude product was not purified and used directly in the following alkylation reactions. Analytical data: PR-HPLC gradient 0-70% B, r_t = 11.8 min; Calculated mass 1407.76 Da, observed mass: 1407.78 Da (**Figure S20a, b**)

For peptide OrexinB analog: the crude product was purified via preparative RP-HPLC. Analytical data: PR-HPLC gradient 0-50% B, r_t = 21.0 min; Calculated mass 2838.59 Da, observed mass: 2838.57 Da, obtained yield = 12.7% (**Figure S3d, e top**).

Chemical mutagenesis of peptide and proteins

Preparation of H4 K20-L/D-SBn

Dha installation: 43 mg of lyophilized $H4_{15\text{-}25}$ K20C peptide crude was dissolved in 1500 µl (final concentration = 20 mM) of reaction buffer (6 M guanidine hydrochloride, 100 mM sodium phosphate, pH 8.4), and 225 µl (4 eq) of DBHDA stock solution (0.5 M in DMF) was added. The crude product was purified by semi-preparative RP-HPLC. Analytical data: PR-HPLC gradient 0-70% B, r_t = 11.6 min; Calculated mass = 1373.78 Da, observed mass = 1373.77, obtained yield = 11% (**Figure S20c, d**).

Functionalizing Dha with benzyl mercaptan: 500 µl peptide (7.3 mM in buffer 6 M guanidine hydrochloride, 100 mM sodium phosphate, pH 8.4) was taken, and 3 µl of benzyl mercaptan (7 eq) was added, with subsequent incubation at RT for 90 min (800 rpm). The following extraction steps were repeated in triplicate: diethyl ether (500 µL) was added to the reaction mixture, lightly vortexed for 15 s, and then centrifuged for 5 min (15000 rpm). Analytical data: PR-HPLC gradient 0-40% B, r_t = 17.9 min and 18.8 min; Calculated mass = 1497.81 Da, observed mass = 1497.82 Da. Obtained yield = 16% (**Figure S20e, f)**

Preparation of H4K20-L-SBn

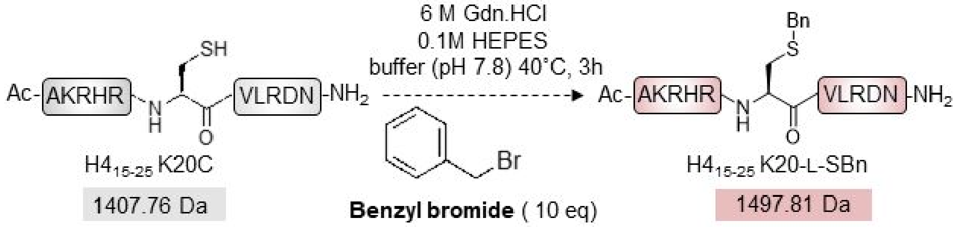

Fig. S29: General scheme for alkylation of $H4_{15\text{-}25}$ K20C.

In the synthesis of H4K20-L-BSn peptide, a general procedure illustrated in (**Figure S29**) was used. 1.5 mg of $H4_{15\text{-}25}$ K20C peptide crude was dissolved in 500 µL of reaction buffer (6 M GdnCl, 1 M HEPES, pH 7.8) to a final concentration of 2.1 mM, followed by the addition of 1.2 µl (10 eq) benzyl bromide. The reaction proceeded at 40 °C by stirring at 1200 rpm for 3 hours. The resulting H4K20-L-BSn peptide was subsequently purified using semi-preparative RP-HPLC. Analytical data: RP HPLC gradient 0-40% B, r_t = 17.9 min; Calculated mass = 1497.81 Da, observed mass = 1497.81 Da. Obtained yield = 11% (**Figure S20g, h)**

Chemical mutagenesis on H3 variants (K9C, K27C, K36C, K79C)

Installing Dha

Dha was introduced to H3 variants K9C, K27C, K36C and K79C using a protocol described by Wright et al.[6] The protein Cys mutant (20 mg of lyophilized powder) was dissolved in 1 ml of degassed reaction buffer 1 (6 M guanidine hydrochloride, 100 mM sodium phosphate, pH 8.4), and 60 mg of DTT was added. The resultant protein solution was shaken at room temperature for 30 min, and excess DTT was removed by passing it through a PD-10 column (equilibrated with the same buffer), 200 µl of DBHDA stock solution (0.6 M in DMF) (91 eq) was added. The reaction was shaken for 60 min at 25 °C at 800 rpm. Next, the excess DBHDA was removed by passing it through a PD-10 column (equilibrated with the same buffer) in the same way as described above. The mixture was then shaken for 30 min at 37 °C at 1000 rpm. Analytical data:
H3K9-Dha: Calculated mass = 15150 Da, observed mass 15149 Da (**Figure S2a).** Used without further characterization.
H3K27-Dha: PR-HPLC gradient 35-70% B, r_t = 22.6 min; Calculated mass = 15150, observed mass = 15150 Da. Obtained yield = 72.5% (Calculated using HPLC analysis of the ratio of starting material to product. Note: This method of quantification was applied to all histone-based modifications unless specified otherwise.) (**Figure S31c, d).**
H3K36-Dha: PR-HPLC gradient 35-70% B, r_t = 22.6 min; Calculated mass = 15150, observed mass = 15150 Da. Obtained yield = 70% (**Figure S32c, d).**
H3K79-Dha: Calculated mass = 15150, observed mass = 15150 Da (**Figure S33b).** Used without further characterization.

Functionalizing Dha with trifluoroethane thiol ($-SCH_2CF_3$)

For introducing $-SCH_2CF_3$ modification to H3 variants (K9C, K27C, K36C and K79C), 20 µl of 2,2,2-trifluoroethanethiol (TFET, 500 eq) was added to the protein (800 µl, ~560 µM), and then lightly vortexed and sonicated. The reaction took place at room temperature for 1.5 h (800 rpm). The following extraction steps were repeated in triplicate: diethyl ether (800 µL) was added to the reaction mixture, lightly vortexed for 15 sec, and then centrifuged for 5 min (15000 rpm).
All variants were purified by HPLC using following methods:

H3K9-SCH_2CF_3: Semi-preparative RP-HPLC was used to purify. Analytical data: RP-HPLC gradient 35-70% B, r_t = 22.6 min; Calculated mass = 15266 Da, observed mass = 15266 Da. Obtained yield = ~33% (based on H3K9C) **(Figure S30b, Figure S15a, b top).**

H3K27-SCH_2CF_3: Semi-preparative RP-HPLC was used to purify. Analytical data: PR-HPLC gradient 35-70% B, r_t = 22.6 min; Calculated mass = 15266 Da, observed mass = 15266 Da. Obtained yield = 61% (based on H3K27-Dha) (**Figure S31e, Figure S15c, d top).**

H3K36-SCH_2CF_3: Semi-preparative RP-HPLC was used to purify. Analytical data: PR-HPLC gradient 35-70% B, r_t = 22.6 min; Calculated mass = 15266 Da, observed mass = 15266 Da. Obtained yield = 55% (based on H3K36-Dha) (**Figure S32e, Figure S16a, b top).**

H3K79-SCH_2CF_3: Semi-preparative RP-HPLC was used to purify. Analytical data: PR-HPLC gradient 35-70% B, r_t = 22.6 min; Calculated mass = 15266 Da, observed mass = 15265 Da. Obtained yield = ~30% (based on H3K79C) **(Figure S33c, Figure S16c, d top).**

Functionalising H3 (K9C, K27C, K36C, K79C)-Dha with other thiol reagents

SBn modification: 500 µL of H3-K9Dha (560 µM in buffer: 6 M guanidine hydrochloride, 100 mM sodium phosphate, pH 8.4) were incubated with 5 µl benzyl mercaptan (152 eq). The protein solution was then reacted at 25 °C for 3 hr at 1000 rpm. The following extraction steps were repeated in triplicate: diethyl ether (500 µL) was added to the reaction mixture, lightly vortexed for 15 s, and then centrifuged for 5 min (15000 rpm). The final product was purified with RP-HPLC; Analytical data: PR-HPLC gradient 35-70% B, r_t = 22.8 min; Calculated mass = 15274 Da, observed mass = 15273 Da. Obtained yield = ~32 % (based on H3K9C) (**Figure S30 a, S2b**).

The same procedures were employed for other modifications with the following adjustments:

SLeu modification: 144 µl of 2-Propanethiol (5500 eq) was added to 500 µL of H3-Dha proteins (560 µM). Analytical data:

H3K9-SLeu: Semi-preparative RP-HPLC was used to purify. Analytical data: RP-HPLC gradient 35-70% B, r_t = 22.5 min; Calculated mass = 15226 Da, observed mass = 15225 Da. Obtained yield = 30% (based on H3K9C) **(Figure 30c, S13a, b top).**

H3K27-SLeu: Semi-preparative RP-HPLC was used to purify. Analytical data: RP-HPLC gradient 35-70% B, r_t = 22.6 min; Calculated mass = 15226 Da, observed mass = 15226 Da. Obtained yield = 34% (based on H3K27C) (**Figure S31f, S13c, d top).**

H3K36-SLeu: Semi-preparative RP-HPLC was used to purify. Analytical data: RP-HPLC gradient 35-70% B, r_t = 22.7 min; Calculated mass = 15226 Da, observed mass = 15225 Da. Obtained yield = 35% (based on H3K36C) (**Figure S32f, S14a, b top).**

H3K79-SLeu: Semi-preparative RP-HPLC was used to purify. Analytical data: RP-HPLC gradient 35-70% B, r_t = 24.7 min; Calculated mass = 15226 Da, observed mass = 15225 Da. Obtained yield = ~28% (based on H3K79C) **(Figure S33d, S14c, d top).**

Dimethyl-lysine mimic modification (K-$_S$me$_2$): Protocols were used from previous studies with minor modifications as detailed below.[7] 184 µL of approximately 5 M 2-(dimethylamino) ethanethiol stock

solution (122 mg dissolved in 160 µL 6 M guanidine and 100 mM sodium phosphate buffer pH 8; 300 eq) were added to 500 µL of H3-Dha proteins (560 µM);

H3K9-$_{S}$me$_2$: Semi-preparative RP-HPLC was used to purify. Analytical data: RP-HPLC gradient 35-70% B, r_t = 22.8 min; Calculated mass = 15255 Da, observed mass = 15255 Da. Obtained yield = 34% (based on H3K9C) **(Figure S30d, S17a, c, top).**

H3K27-$_{S}$me$_2$: Semi-preparative RP-HPLC was used to purify. Analytical data: RP-HPLC gradient 35-70% B, r_t = 22.8 min; Calculated mass = 15255 Da, observed mass = 15255 Da. Obtained yield = 28% (based on H3K27C) **(Figure S31g, Figure S17c, d top).**

H3K36-$_{S}$me$_2$: Semi-preparative RP-HPLC was used to purify. Analytical data: RP-HPLC gradient 35-70% B, r_t = 22.7 min; Calculated mass = 15255 Da, observed mass = 15255 Da. Obtained yield = 35% (based on H3K36C) (**Figure S32g, S18a, b top**).

H3K79-$_{S}$me$_2$: Semi-preparative RP-HPLC was used to purify. Analytical data: RP-HPLC gradient 35-70% B, r_t = 24.2 min; Calculated mass = 15255 Da, observed mass = 15255 Da. Obtained yield = ~28% (based on H3K36C) **(Figure S33e, S18c, d top).**

H3K9-SChex modification:10 µL of Cyclohexane-thiol (300 eq) were added to 500 µL of H3-Dha proteins (560 µM).

Semi-preparative RP-HPLC was used to purify. Analytical data: RP-HPLC gradient 35-70% B, r_t = 22 min; Calculated mass = 15266 Da, observed mass = 15266 Da. Obtained yield = 41% (based on H3K9C) **(Figure S7a, c top).**

H3K9-SCpen modification:10 µL of Cyclopentane-thiol (330eq) were added to 500 µL of H3-Dha proteins (560 µM).

Semi-preparative RP-HPLC was used to purify. Analytical data: RP-HPLC gradient 35-70% B, r_t = 21.9 min; Calculated mass = 15252 Da, observed mass = 15252 Da. Obtained yield = 30.7% (based on H3K9C) **(Figure S7b, d top).**

H3K9-SPh modification:100 µL of Thiophenol (3500eq) were added to 500 µL of H3-Dha proteins (560 µM). The reaction took place at 37 °C for 2 days (800 rpm)

Semi-preparative RP-HPLC was used to purify. Analytical data: RP-HPLC gradient 35-70% B, r_t = 22.2 min; Calculated mass = 15260 Da, observed mass = 15260 Da. Obtained yield = 34.1% (based on H3K9C) **(Figure S8a, c top).**

H3K9-SF$_2$Bn modification:20 µL of 3,5-Difluorobenzyl-mercaptan (DFBM, 530 eq) were added to 500 µL of H3-Dha proteins (560 µM). The reaction took place at 25 °C for 3h (800 rpm)

Semi-preparative RP-HPLC was used to purify. Analytical data: RP-HPLC gradient 35-70% B, r_t = 21.9 min; Calculated mass = 15310 Da, observed mass = 15310 Da. Obtained yield = 30.1% (based on H3K9C) **(Figure S8b, d top).**

H3K9-isoHis modification: Protocols were used from previous studies with minor modifications as detailed below.[8] 11 mg of imidazole (580eq) were added to 500 µL of H3-Dha proteins (560 µM). The reaction took place at 37 °C for 2 days (800 rpm).

Semi-preparative RP-HPLC was used to purify. Analytical data: RP-HPLC gradient 35-70% B, r_t = 22.1 min; Calculated mass = 15218 Da, observed mass = 15219 Da. Obtained yield = 39.2% (based on H3K9C **(Figure S9a, c top).**

H3K9-SAllyl modification:10 μL of Allyl mercaptan (430 eq) were added to 500 μL of H3-Dha proteins (560 μM).
Semi-preparative RP-HPLC was used to purify. Analytical data: RP-HPLC gradient 35-70% B, r_t = 22 min; Calculated mass = 15224 Da, observed mass = 15224 Da. Obtained yield = 41% (based on H3K9C) **(Figure S9b, d top).**
H3K9-SClp modification:5 μL of 3-Chloro-1-propanethiol (180 eq) were added to 500 μL of H3-Dha proteins (560 μM). The reaction took place at 37 °C for 2 days (800 rpm)
Semi-preparative RP-HPLC was used to purify. Analytical data: RP-HPLC gradient 35-70% B, rt = 22.1 min; Calculated mass = 15260 Da, observed mass = 15260 Da. Obtained yield =39% (based on H3K9C) **(Figure S10a, c top).**
H3K9-SAlkyne modification:5 μL of pH adjusted (pH 9) in-situ generated propargylthiol (1 g/ml) (2470 eq) were added to 50 μL of H3-Dha proteins (560 μM).
Semi-preparative RP-HPLC was used to purify. Analytical data: RP-HPLC gradient 35-70% B, r_t = 22.1 min; Calculated mass = 15222 Da, observed mass = 15222 Da. Obtained yield =39% (based on H3K9C) **(Figure S10b, d top).**
H3K9$_S$ac modification:3 μL of N-Acetylcysteamine (100 eq) were added to 500 μL of H3-Dha proteins (560 μM).
Semi-preparative RP-HPLC was used to purify. Analytical data: RP-HPLC gradient 35-70% B, r_t = 22.1 min; Calculated mass = 15269 Da, observed mass = 15269 Da. Obtained yield =39% (based on H3K9C) **(Figure S11a, b top).**

Chemical mutagenesis on GFP-M233C

Installing Dha

GFP-M233-Dha was prepared as previously described with some minor modifications.[9] 0.5 M TCEP (1.5 μL) was added to 150 μL GFP (~50 uM, in 20 mM sodium phosphate, pH 8.4), and the solution was shaken at room temperature for 1 h (400 rpm). After this time, the protein solution was desalted via buffer exchange on Vivaspin 10 kDa spin concentrator (Cytiva) (with 20 mM sodium phosphate, pH 8.4) to remove excess TCEP. Subsequently, 20 μL 0.6 M DBHDA in DMF (1600 eq) was added to the reduced protein. The reaction was conducted at room temperature for 1.5 h while shaking at 800 rpm. Any remaining precipitate was removed by centrifugation (2 min, 15000 rpm). The protein solution was passed through a PD MiniTrap G-25 column (GE Healthcare) and eluted with 1 mL of the same buffer. The resulting protein solution was concentrated back down to 150 μL using the same spin concentrator. The protein was shaken at 37 °C for a final 30 min (1000 rpm) to promote elimination. The sample was then analysed by SDS page (**S23b right)** and HRMS (**Figure S23d)**. Analytical data: Calculated mass for GFP-M233-Dha = 27957 Da (27826 Da -Met); observed mass = 27957 Da (27826 Da -Met). Obtained yield = 83% (calculated based on GFP-Dha concentrations)**.**

Functionalising Dha with benzyl mercaptan

For making GFP-SBn, benzyl mercaptan (2 µL, 17 µmol, 3407 eq), was added to the protein (100 µL 40 µM), and then the cloudy green emulsion was shaken at room temperature for 1.5 h (800 rpm). The following extraction steps were repeated in triplicate: diethyl ether (200 µL) was added to the reaction mixture, lightly vortexed for 15 s, and then centrifuged for 5 min (15000 rpm). The sample was then analysed by HRMS **(Figure S24b, top).** Analytical data: Calculated mass for GFP-SBn = 28081 Da (27950 Da -Met); observed mass = 28081 Da. The obtained yield is 64% (The yields were quantified by measuring the protein amounts before and after reactions. Protein amounts were determined using the formula: Concentration (mg/ml) * Volume (ml). Concentrations were measured using a NanoDrop at an absorbance wavelength of 280 nm. The concentration of GFP was calculated from the absorbance readings using an extinction coefficient of 55,000 M^{-1} cm^{-1}.[10]This method was consistently applied for all GFP quantifications (**Figure S24a top).**

GFP-SAllyl

1 µL of Allyl mercaptan (2400 eq) was added to the protein (100 µL 40 µM) and the mixture was shaken at 37 °C for overnight (800 rpm). The following extraction steps were repeated in triplicate: diethyl ether (200 µL) was added to the reaction mixture, lightly vortexed for 15 s, and then centrifuged for 5 min (15000 rpm). Analytical data: Calculated mass for GFP-SAllyl = 28031 Da (27900 Da -Met); observed mass = 28032 Da (27901 Da -Met). 28081 Da (27950 Da -Met); Obtained yield = 60% (Calculated based on GFP-Dha concentration) **(Figure S25a top).**

GFP_SGlc

150 µL of 1 M 1-Thio-β- D-glucose sodium (pH 8) (30000 eq) were added to the protein (100 µL 40 µM) and he mixture was shaken at 25 °C for 2h (800 rpm) The following buffer exchange was used to desalt the reaction mixture, repeated in triplicate: 20 mM sodium phosphate buffer (450 µL, pH 8.5) was added to the reaction mixture in a Vivaspin 10 kDa spin concentrator (Cytiva) and centrifuged for 3 min (15000 rpm) where flowthrough was discarded. The resulting 50 µL solution (1.05 mg/mL) was analysed by HRMS. Analytical data: Calculated mass for GFPsGlc = 28153 Da (28022 Da -Met); observed mass = 28154 Da (28022 Da -Met). Obtained yield = 65%
(Calculated based on GFP-Dha concentration) **(Figure S25c, d top).**

Chemical mutagenesis on Trx-SGTA^C protein

Installing Dha

0.5 M DTT (10 µL) was added to 1000 µL of Trx-SGTA^C (S252C) (~640 uM, in 20 mM potassium phosphate (pH 8), 300 mM NaCl), and the solution was kept at 4 °C overnight. Then the protein was desalted by passing it through a PD-10 column (equilibrated with the same buffer). After this, the protein was diluted into 9 mL of the same buffer to ~70 uM, then 50 mM of DBHDA (0.6 M in DMF) was added. The reaction was shaken for 3 h at 25 °C at 800 rpm. Next, the excess DBHDA was removed by passing it through a PD-10 column (equilibrated with the same buffer) as described

above. The mixture was then shaken for 30 minutes at 37 °C at 1000 rpm to promote elimination. The sample was then analysed by SDS-PAGE **(Figure S27a, middle)** and HRMS. Analytical data: Calculated mass for Thioredoxin- Trx-SGTA^{C}-Dha = 24980; observed mass = 24978. Obtained yield = 72% (The yields were quantified by measuring the protein amounts before and after reactions. Protein amounts were determined using the formula: Concentration (mg/ml) * Volume (ml). Concentrations were measured using a NanoDrop spectrophotometer.) **(Figure S27b)**

<u>Functionalizing Dha with trifluoroethane thiol (-SCH_2CF_3)</u>

For introducing -SCH_2CF_3 modification, 10 µl of 2,2,2-trifluoroethanethiol (TFET, 1400 eq) was added to the protein (~80.3 µM), and then lightly vortexed and sonicated. The reaction took place at room temperature for 5 h (1000 rpm). The following extraction steps were repeated in triplicate: diethyl ether (500 µL) was added to the reaction mixture, lightly vortexed for 15 sec, and then centrifuged for 5 min (15000 rpm). The sample was then analysed by SDS-PAGE **(Figure S27a, right)** and HRMS. Analytical data: Calculated mass Trx-SGTA^{C}- SCH_2CF_3 = 25095; observed mass = 25094. Obtained yield = 93% (The yields were quantified by measuring the protein amounts before and after reactions. Protein amounts were determined using the formula: Concentration (mg/ml) * Volume (ml). Concentrations were measured using a NanoDrop spectrophotometer.) **(Figure S28c, top).**

Chemical mutagenesis on Ddx4^{N}-F182C

<u>Installing Dha</u>

10 µL of Methyl 2,5-Dibromopentanoate (MDBP)[11] (550 eqv) was added to 500 µL of Ddx4^{N}-F182C (~69 uM, in 100 mM potassium phosphate (pH 8), 500 mM NaCl), the reaction was shaken for 1 h at 25 °C at 800 rpm. The resulting Ddx4^{N}-F182-Dha was purified with HPLC. Analytical data: Preparative RP HPLC gradient 25-35% B, r_t = 26.04 min; The sample was then analysed by SDS-PAGE **(Figure S26a, middle)** and HRMS. Analytical data: Calculated mass for Ddx4^{N}-F182-Dha = 25693; observed mass = 25692. Obtained yield = 44% (The yields were quantified by measuring the protein amounts before and after reactions. Protein amounts were determined using the formula: Concentration (mg/ml) * Volume (ml). Concentrations were measured using a NanoDrop spectrophotometer.) **(Figure S26c).**

<u>Functionalizing Dha with trifluoroethane thiol (-SF_2Bn)</u>

For introducing -SF_2Bn modification, 1 mg lyophilized Ddx4 ^{N}-Dha was dissolved in 300 µL buffer (20 mM Sodium phosphate (pH 8), 500 mM NaCl). 1 µl of 3,5-Difluorobenzyl-mercaptan (DFBM, 3800 eq) was added to the protein, and the solution lightly vortexed and sonicated. The reaction took place at room temperature with shaking overnight (1000 rpm). The following extraction steps were repeated in triplicate: diethyl ether (500 µL) was added to the reaction mixture, lightly vortexed for 15 sec, and then centrifuged for 5 min (15000 rpm). The sample was then analysed by SDS-PAGE **(Figure S26a, right) and** HRMS. Analytical data: Calculated mass for Ddx4 ^{N}-F182- SF_2Bn = 25853; observed mass

= 25853. Obtained yield = 91%. (The yields were quantified by measuring the protein amounts before and after reactions. Protein amounts were determined using the formula: Concentration (mg/ml) * Volume (ml). Concentrations were measured using a NanoDrop spectrophotometer. calculated based on Dha concentrations) **(Figure S26d, top).**

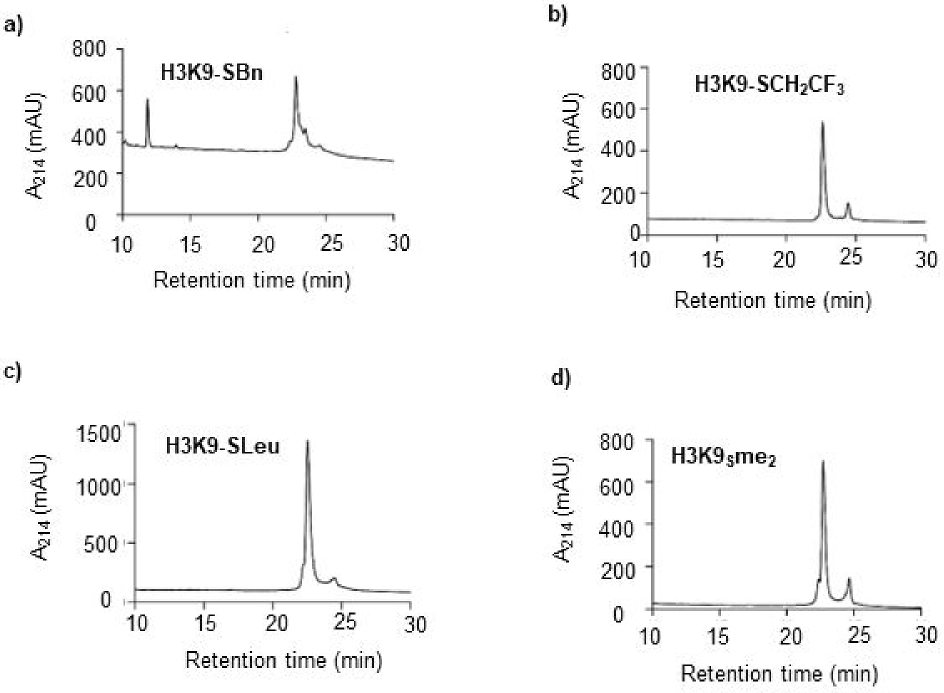

Fig. S30: HPLC analysis of chemical modifications of H3K9 species.

(a) H3K9-SBn. (b) H3K9-SCH_2CF_3. (c) H3K9-SLeu. (d) H3K9$_S$me$_2$.

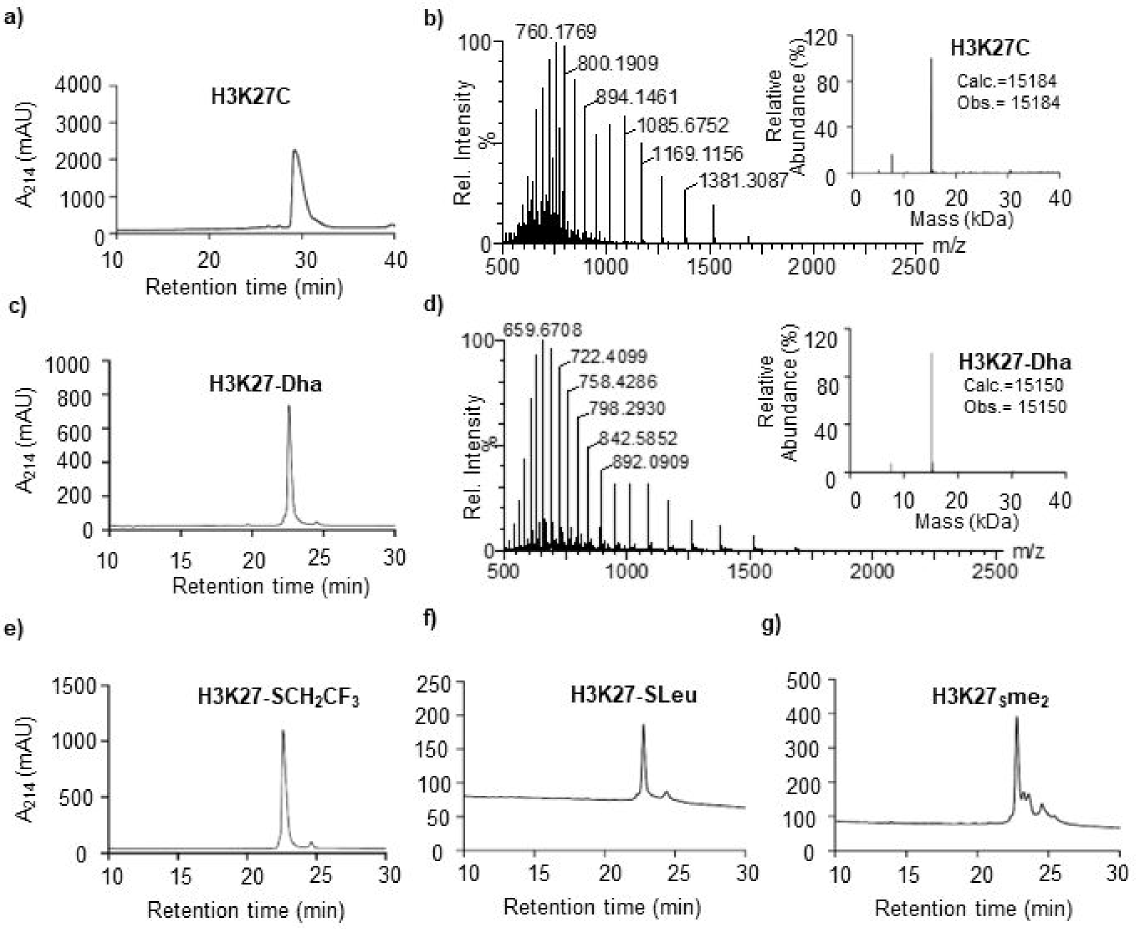

Fig. S31: Analysis of chemical modifications of H3K27 species.

(a) HPLC analysis of H3K27C. (b) Mass spectrum of H3K27C (calculated mass = 15184 Da, observed mass = 15184 Da). (c) HPLC analysis of H3K27-Dha. (d) Mass spectrum of H3K27-Dha (calculated mass = 15150 Da, observed mass = 15150 Da). (e) HPLC analysis of H3K27-SCH_2CF_3. (f) HPLC analysis of H3K27-SLeu. (g) HPLC analysis of H3K27$_S$me$_2$.

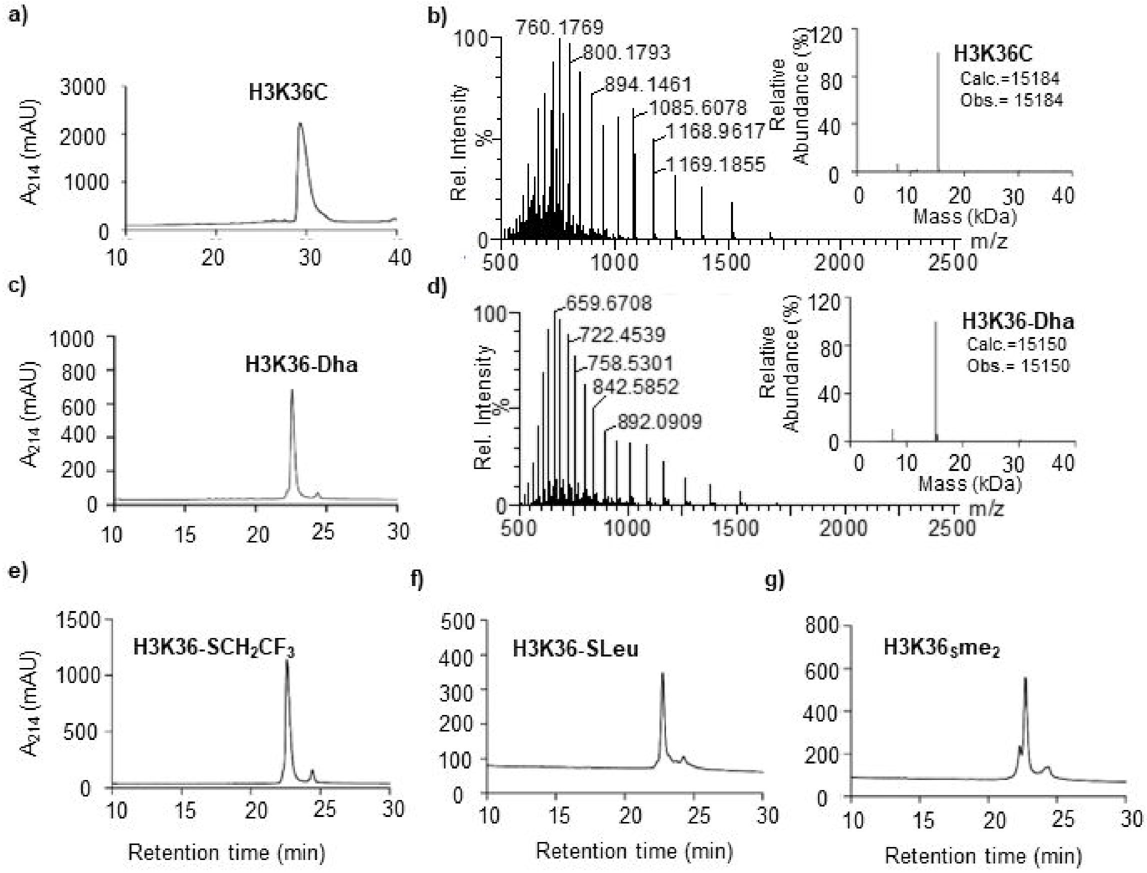

Fig. S32: Analysis of chemical modifications of H3K36.

(a) HPLC analysis of H3K36C. (b) Mass spectrum of H3K36C (calculated mass = 15184 Da, observed mass = 15184 Da). (c) HPLC analysis of H3K36-Dha. (d) Mass spectrum of H3K36-Dha (calculated mass = 15150 Da, observed mass = 15150 Da). (e) HPLC analysis of H3K36-SCH_2CF_3. (f) HPLC analysis of H3K36-SLeu. (g) HPLC analysis of H3K36$_S$me$_2$.

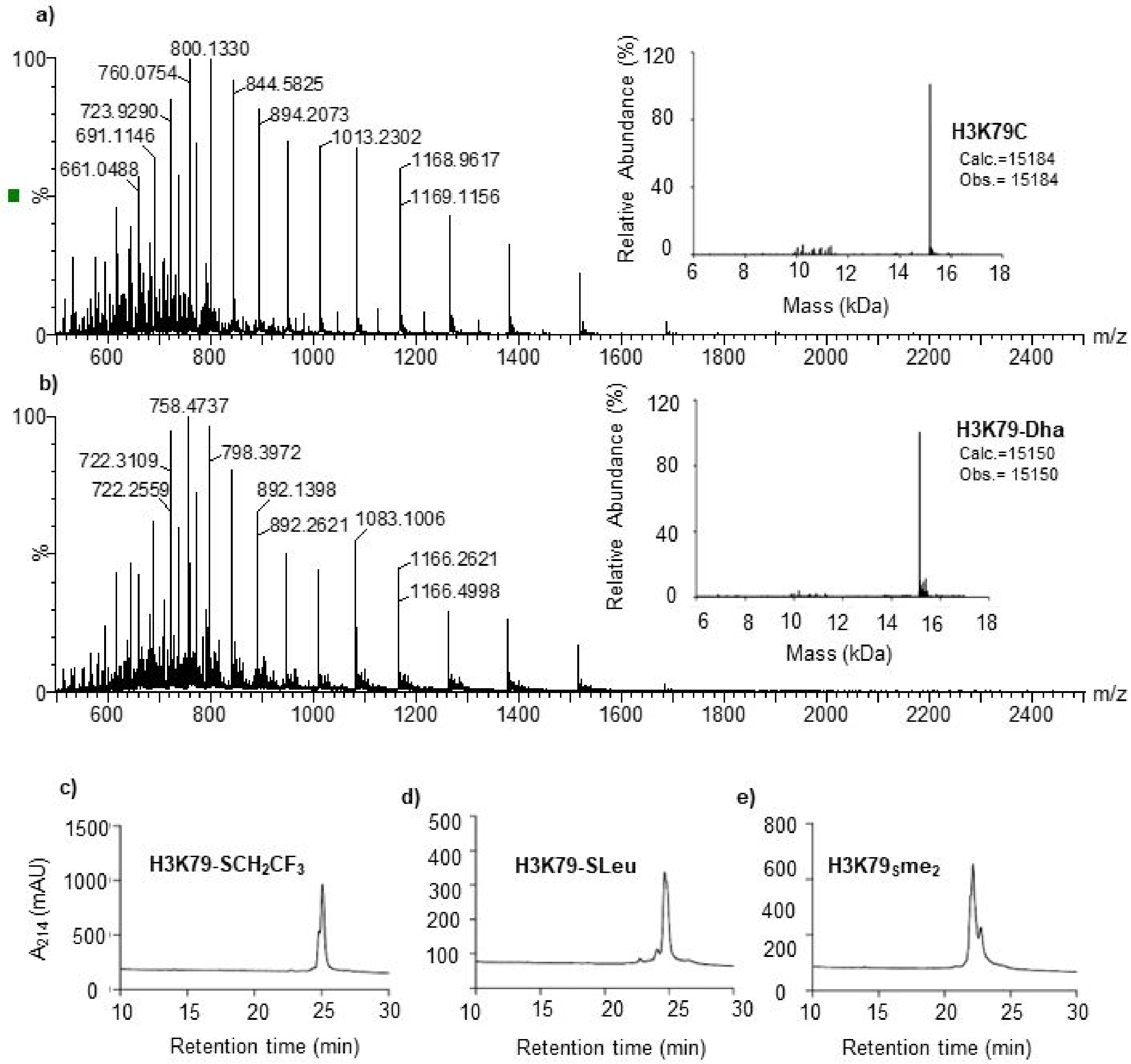

Fig. S33: Analysis of chemical modifications of H3K79 species.

(a) Mass spectrum of H3K79C (calculated mass = 15184 Da, observed mass = 15184 Da). (b) Mass spectrum of H3K79-Dha (calculated mass = 15150 Da, observed mass = 15150 Da). (c) HPLC analysis of H3K79-SCH_2CF_3. (d) HPLC analysis of H3K79-SLeu. (e) HPLC analysis of H3K79$_S$me$_2$.

Cleavage reactions with AD-P

Analytical cleavage of H3 variants by AD-P

~4 mg/mL stock solutions of H3 variants were prepared by dissolving 2 mg lyophilized protein powder into 500 µL 0.1 M sodium carbonate buffer (pH 9.3). A 1 M solution of L-Arg was added dropwise to a final concentration of 125 mM (H3K9, K27 & K36 variants) or 150 mM (H3K79 variants). Reaction mixtures contained 20 µL histone +L-Arg stock, 20 µL sodium carbonate buffer (0.1 M, pH 9.3) and 20 µL AD-P (0.8 mg/mL). The reactions (42~50 mM L-Arg) were incubated at 30 °C for 1 h (or the specified time for time course reactions). 1 µL aliquots of the resulting reaction mixtures were diluted 100-fold in H_2O for HRMS analysis, and the remaining reaction volume was quenched with 4X SDS loading buffer for subsequent gel analysis.
For analytical RP-HPLC analysis of K27-SCH_2CF_3 and K36- SCH_2CF_3 cleavage, the same reaction procedure was conducted. The reaction mixture was injected into the analytical HPLC using a C3 column, with a gradient of 35-70% B.

Preparative cleavage of H3 variants by AD-P

11.7 mg/mL stock solutions of H3 variants were prepared by dissolving 11.7 mg lyophilized protein powder into 1 ml 0.1 M sodium carbonate buffer (pH 9.3). A 1 M solution of L-Arg was added dropwise to a final concentration of 125 mM. Reaction mixtures contained done by mixing 100 µL histone stock, 100 µL sodium carbonate buffer (0.1 M, pH 9.3) and 100 µL AD-P (0.8 mg/mL). The reactions (42 mM L-Arg) were incubated at 30 °C for overnight. Remained uncleaved L-isomers were further purified with semi-preparative RP-HPLC using C3 Column, gradient 30-70% B.

H4K20$_{15\text{-}25}$SBn peptide cleavage with AD-P

~1.3 mg/mL (868 µM) H4K20$_{15\text{-}25}$SBn stock solutions were prepared by dissolving 0.75 mg lyophilized powder of H4K20$_{15\text{-}25}$SBn into 500 µl water.
For full cleavage, a reaction mixture was prepared by mixing 20 µL peptide stock (final concentration 347 µM), 10 µL carbonate buffer (0.1 M, pH 9.3), 20 µL AD-P (2 mg/mL in 0.1 M HEPES pH 7.6, 100 mM NaCl, 10% glycerol, final concentration 20.4 µM). The reaction was conducted at 30 °C for 12 hrs.
For time course cleavage, a reaction mixture was prepared by mixing 5 µL peptide stock (final concentration 217 µM), 10 µL carbonate buffer (0.1 M, pH 9.3), 10 µL AD-P (2 mg/mL in 0.1 M HEPES pH 7.6, 100 mM NaCl, 10% glycerol, final concentration 20.4 µM). The reaction was conducted at 30 °C for various lengths of time (0, 5, 15, 30, 60, 120, 180 mins).
The reactions were quenched with 50% (v/v) quenching solution (50% methanol, 5% TFA). The reaction products were analysed via HPLC using a C3 analytical column, and individual peaks were analysed with HRMS. Controls in the absence of AD-P. Analytical data for cleaved product: RP-HPLC gradient 0-40% B, (for L-isomer) r_t = 17.6 min; calculated mass = 1497.81 Da, observed mass = 1497.82 Da; (for cleaved N-terminal D-isomer) r_t = 13.3 min; calculated mass = 901.47, observed mass = 901.47 Da; (for cleaved C-terminal D-isomer) r_t = 4.94 min; calculated mass = 614.35 observed mass = 614.36 Da **(Figure 4b,c, S20c).**

GFP-SBn cleavage by AD-P

The small scale (10 µL) reaction mixtures contained 15 µM AD-P and 8 µM GFP-SBn, in a 0.1 M sodium carbonate buffer. Control reactions were prepared in the same way, with phosphate buffer used to supplement missing volumes. GFP-M233C in the negative controls were reduced with TCEP prior to AD-P addition. The reactions were incubated at 30 °C for 2h. 1 µL aliquots of the resulting reaction mixtures were diluted 100-fold in H_2O for HRMS analysis, and the remaining reaction volume was quenched with 4X SDS loading buffer for subsequent analysis by SDS-PAGE.

GFP-SAllyl cleavage by AD-P

The small scale (60 µL) reaction mixtures contained 6.9 µM AD-P and 17 µM GFP- SAllyl, in a 0.1 M sodium carbonate buffer (pH 9.3). The reactions were incubated at 30 °C for 1 h. 1 µL aliquots of the resulting reaction mixtures were diluted 100-fold in H_2O for HRMS analysis, and the remaining reaction volume was quenched with 4X SDS loading buffer for subsequent analysis by SDS-PAGE.

GFP_SGlc cleavage by AD-P

The small scale (60 µL) reaction mixtures contained 6.9 µM AD-P and 17 µM GFP- GFP_SGlc, in a 0.1 M sodium carbonate buffer (pH 9.3) and 0.2 M HEPES buffer (pH8.0). The reactions were incubated at 30 °C for Overnight. 1 µL aliquots of the resulting reaction mixtures were diluted 100-fold in H_2O for HRMS analysis, and the remaining reaction volume was quenched with 4X SDS loading buffer for subsequent analysis by SDS-PAGE.

Analytical cleavage of $Ddx4^N$-SF_2Bn by AD-P

The small scale (27 µL) reaction mixtures contained 1.5 µM AD-P and 23 µM $Ddx4^N$-SF_2Bn, in a 0.1 M sodium carbonate buffer (Containing 500 mM KCL, pH 9.3). Control reactions were prepared in the same way, with H_2O used to supplement missing volumes. $Ddx4^N$-F182C in the negative controls were reduced with TCEP prior to AD-P addition. The reactions were incubated at 30 °C for 1-2 h. 1 µL aliquots of the resulting reaction mixtures were diluted 100-fold in H_2O for HRMS analysis, and the remaining reaction volume was quenched with 4X SDS loading buffer for subsequent analysis by SDS-PAGE.

Analytical cleavage of Trx-$SGTA^C$-SCH_2CF_3 by AD-P

The small scale (60 µL) reaction mixtures contained 6.8 µM AD-P and 13 µM Trx-$SGTA^C$-SCH_2CF_3, in a 0.1 M sodium carbonate buffer (pH 9.3). Control reactions were prepared in the same way, with H_2O used to supplement missing volumes. Unmodified Trx-$SGTA^C$S*252C* in the negative controls was reduced with TCEP prior to AD-P addition. The reactions were incubated overnight at 30 °C. 1 µL aliquots of the resulting reaction mixtures were diluted 100-fold in H_2O for HRMS analysis, and the remaining reaction volume was quenched with 4X SDS loading buffer for subsequent analysis by SDS-PAGE.

Preparative cleavage of Trx-SGTA^{C}-SCH_2CF_3 by AD-P

The large-scale (9 mL) reaction mixtures contained 3 mL of AD-P (0.8 mg/mL), 3 mL of Trx-SGTA^{C}-SCH_2CF_3 (2.2 mg/mL), and 3 mL of 0.1 M sodium carbonate buffer (pH 9.3). The reaction was incubated at 30 °C overnight. Any remaining uncleaved L-isomers were further purified. Specifically, the sample was concentrated to 400 µL and loaded onto a Superdex 75 gel filtration column using a 1 mL capillary loop. The sample was added to the column at a rate of 1 mL/min and eluted with Size Exclusion Buffer (10 mM potassium phosphate (pH 8), 100 mM NaCl) at a rate of 0.8 mL/min and collected in 0.7 mL fractions. Fractions were analyzed by SDS-PAGE and HRMS for protein content and then concentrated using a 10 kDa MWCO centrifugal concentrator to 2 ml at a concentration of 1.164 mg/ml. The obtained yield was 37%. The yields were quantified by measuring the protein amounts before and after purification. Total protein amounts were determined using the formula: Concentration (mg/ml) * Volume (ml). Concentrations were measured using a NanoDrop spectrophotometer, The L/D- isomers were quantified by determining the band intensities with ImageJ and calculating the relative percentage of cleaved and uncleaved species. Relative concentrations were obtained by dividing the relative band intensities by the molecular weight of the cleaved and uncleaved species.

NMR analysis methods

NMR analysis of diastereotopic peptides

All experiments were performed using a Bruker Avance 800 spectrometer equipped with a CP TCI 800S4 H-C/N-D-05 helium-cooled cryoprobe at a temperature of 303 K, with an L/D- peptide mixture concentration of 1 mM in 20 mM carbonate buffer (pH 9.3) and 10% D_2O. Assignment of the peaks for the modified Cys-BSn residue within the peptide was conducted using water-suppressed ^{1}H 1D (zgesgp), water-suppressed ^{1}H-^{1}H COSY (cosygpprqf), ^{1}H-^{13}C HSQC (hsqcetgpsp.3 and hsqcedetgp) and ^{1}H-^{13}C HMBC (hmbcgplpndprqf).

Time course measurements were performed using a Bruker Avance 800 spectrometer equipped with a CP TCI 800S4 H-C/N-D-05 helium-cooled cryoprobe at a temperature of 303 K, with an L/D- peptide mixture concentration of 1 mM and alkaline D-peptidase concentration of 12 µM in 20 mM carbonate buffer (pH 9.3) and 10% D_2O. In-situ reaction monitoring experiments were timed from addition of the enzyme to the peptide sample to account for the delay in recording the first time point from tuning and shimming the sample in the spectrometer.

To track the cleavage of the peptide by alkaline D-peptidase over time, alternating water-suppressed ^{1}H 1D (zgesgp) and ^{1}H-^{13}C HSQC (hsqcetgpsp) experiments were performed over the course of 17 hr. ^{1}H 1D experiments were performed using 16 scans (ns) with a spectral window of 12.5 ppm to -3 ppm. ^{1}H-^{13}C HSQC experiments were performed using 4 scans (ns), 16 dummy scans (ds) and a coupling constant of 145 Hz (cnst2). The spectral window for the ^{1}H-^{13}C HSQC experiment was 9.4 ppm to 0 ppm for the ^{1}H (F2) axes and 140 ppm to 10 ppm for the ^{13}C (F1) axes. In the F2 and F1 frequency axes, 2048 and 256 data points were collected, respectively.

^{19}F-NMR analysis of H3K36-SCH_2CF_3

Experiments were performed using a Bruker Avance 400 spectrometer equipped with a PA BBO 400S1 BBF-H-D-05 Z SP probe at a temperature of 298 K, with 30 μM H3K36-SCH_2CF_3 in 0.1 M carbonate buffer (pH 9.3). Fluorine spectra were recorded using the pulse program zgfhigqn.2 with a transmitter offset frequency (o1p) of -100 ppm and a spectral width (sw) of 240 ppm.

Synthesis of prop-2-ynyl *S*-thioacetate

The method for synthesis of prop-2-ynyl *S*-thioacetate was adapted from literature.[12] To a cold (0 °C), stirred suspension of NaH (1.68 g of a 60% oil suspension, 42 mmol) in anhydrous THF (60 mL), a solution of thioacetic acid (2.85 mL, 40 mmol) in THF (10 mL) was added dropwise. The mixture was stirred for 30 minutes at r.t. (until hydrogen gas evolution ceased and the solution became transparent). After cooling the solution again to 0 °C, propargyl bromide (80% w/w in Toluene) (2.14 mL, 20 mmol) was added dropwise; the mixture was stirred for 45 minutes at r.t. The reaction mixture was quenched using water (50 mL) and then extracted with diethyl ether (3 x 50 mL). The combined organic layers were washed with water (3 x 50 mL) and dried with $MgSO_4$. After filtration and removal of the solvents in vacuo, the yellow crude product was dry loaded on a silica gel column, washed with hexanes and eluted with 5:95 diethyl ether:hexane. A light orange oil of prop-2-ynyl *S*-thioacetate (855 mg, 7.5 mmol, 38%) was achieved. ^1H NMR (400 MHz, $CDCl_3$) δ 3.64 (d, J = 2.7 Hz, 2H), 2.36 (s, 3H), 2.20 – 2.14 (m, 1H). ^{13}C NMR (101 MHz, $CDCl_3$) δ 193.86 (Cq), 78.86 (Cq), 70.97 (CH), 30.24 (CH_3), 17.55 (CH_2). RP-HPLC gradient 0-70% B, rt = 10.8 min.

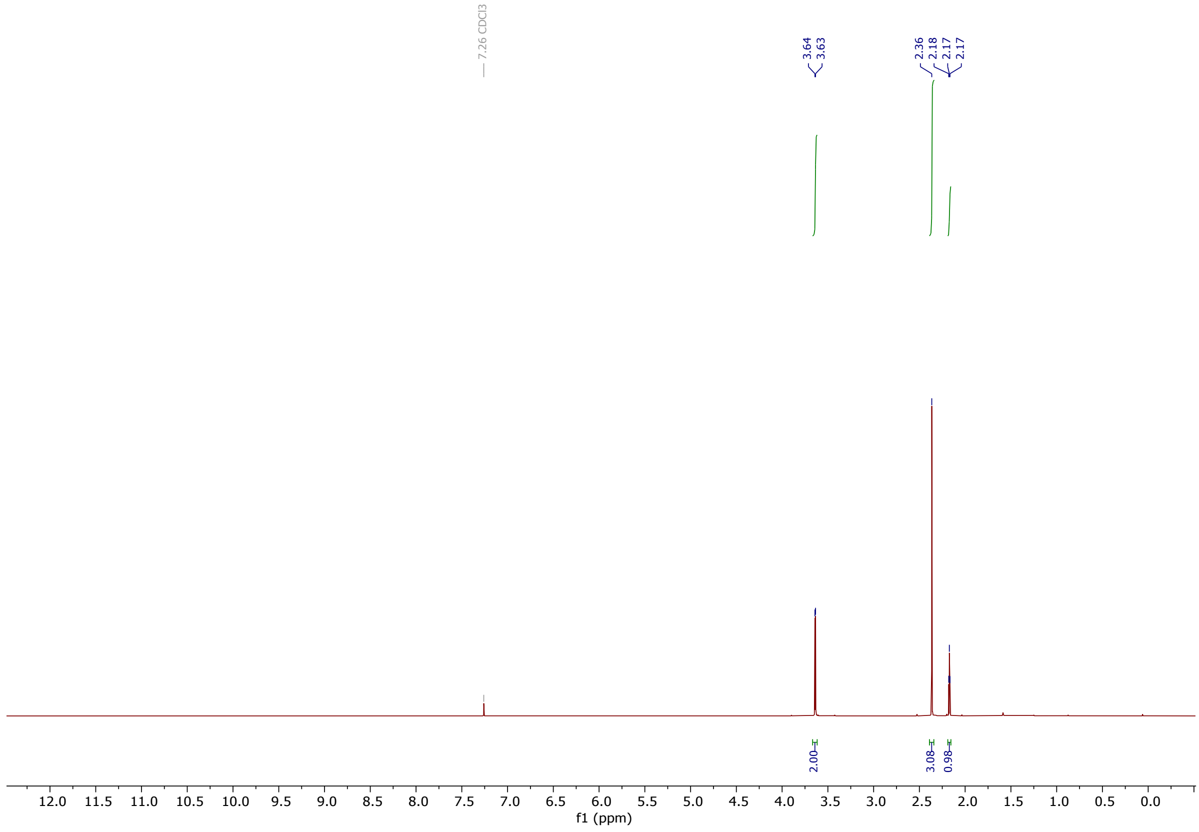

Fig. S34: ^{1}H-NMR spectra of purified prop-2-ynyl *S*-thioacetate rotated at 20 Hz in $CDCl_3$ at 298 K.

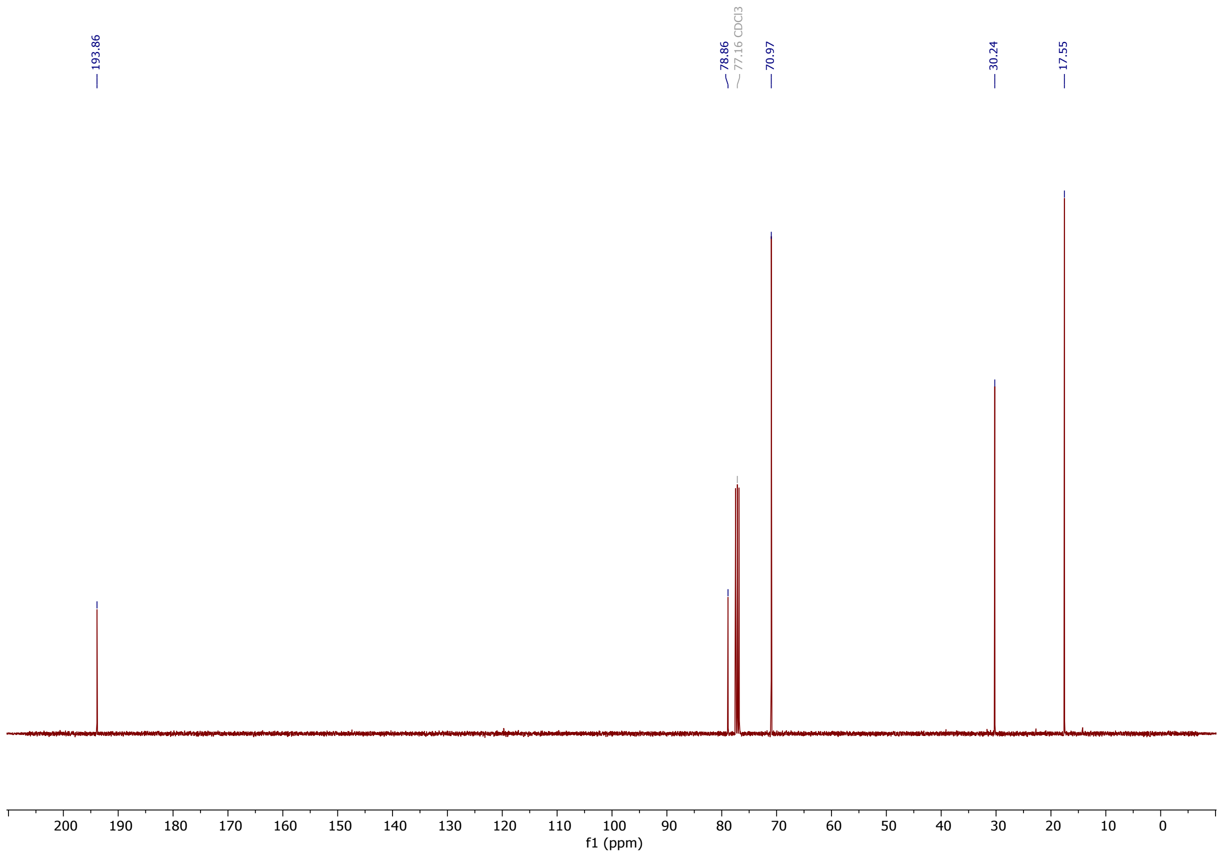

Fig. S35: ^{13}C-NMR spectra of purified prop-2-ynyl *S*-thioacetate in $CDCl_3$ at 298 K.

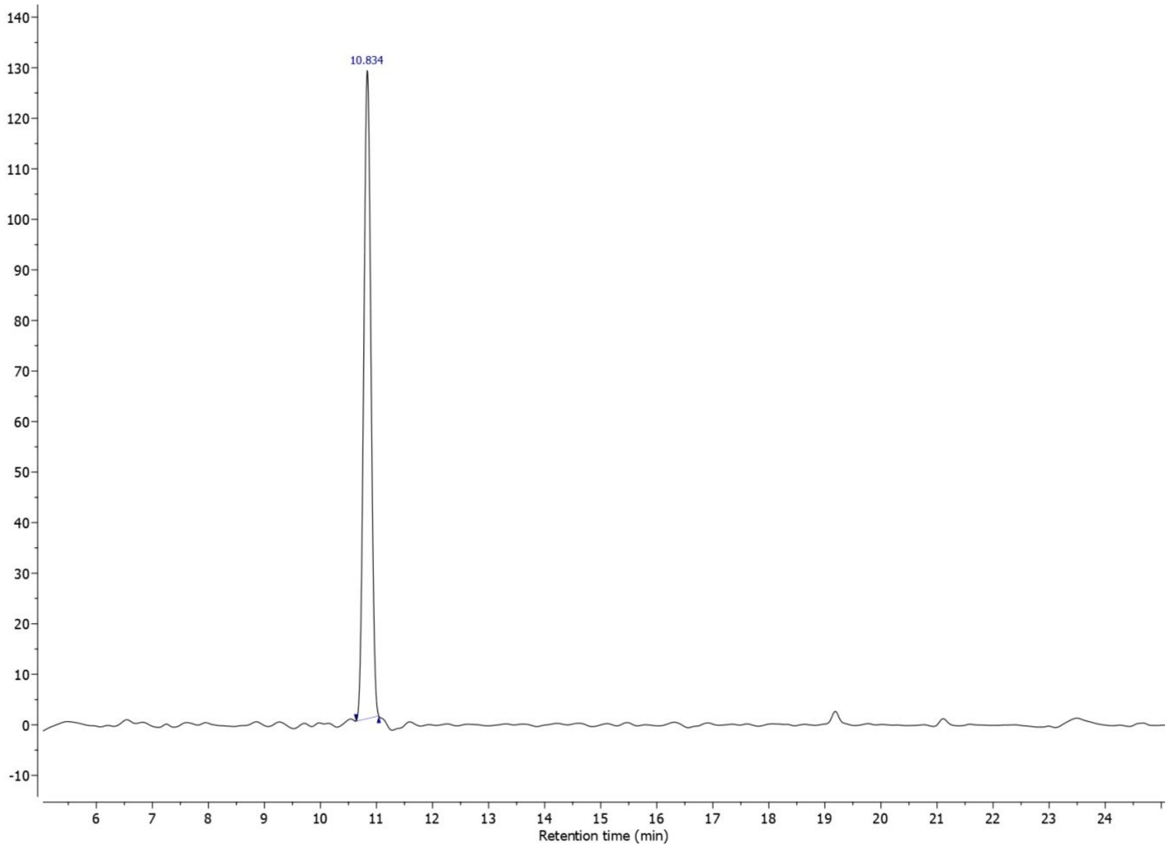

Fig. S36: HPLC analysis of prop-2-ynyl *S*-thioacetate.

Synthesis of propargyl thiol

Synthesis of propargyl thiol was adapted from work done by Drueckhammer *et al.*[13] 2-Propyne-*S*-thioacetate (109 mg, 0.9 mmol) was added into a flask and dissolved in 1.0 mL methanol. After Ar-flushing, NaOH (47 mg, 8.37 mmol) dissolved in 0.5 mL methanol was added (upon addition solution went orange from a pale orange). The reaction was stirred at r.t. for 30 minutes and then filtered using gravity filtration. The reaction was quenched by lowering the pH to 9 using 1 M HCl that was cooled on ice. The resulting compound was used for an *in situ* reaction with H3K9Dha without further workup or purification.

Protein Sequences

<u>ADP-6His</u>

<u>DNA sequence</u>

Codon-optimised for expression in *E. coli*, NCBI Gene ID: D86380.1 [1]
ATGAAAACACGTAGTCAAATTACATGTGCAAGTCTTGCCCTTTTAATAGCTGGAAGTTCCCTGTTA
TACACAACGCAAACCTTAATTGTAAAAGCAGAACCTACTCAAAGTGTATCTAGTTCGGTACAAACA
AGTACTCAACGAGATCGTAATTCTGTCAAGCAAGCAGTGCGGGATACATTGCAACTTGGATTCCC
GGGGATACTTGCTAAAACTTCTGAGGGTGGAAAAACATGGAGTTATGCCGCTGGGGTAGCGAAT

CTGAGCAGCAAGAAACCCATGAAAACAGATTTTCGCTTTCGCATTGGTAGCGTGACGAAGACGTT
CACGGCAACAGTTGTACTTCAATTAGCCGAAGAGAACCGCTTGAATCTAGACGACTCTATTGAAA
AATGGTTGCCTGGTGTCATTCAAGGAAATGGGTATGATGATAAACAGATTACTATCCGGCAATTA
TTGAACCACACAAGTGGTATCGCTGAATACACAAGGTCAAAAAGTTTTGATCTTATGGATACTAAA
AAATCGTATAGGGCTGAAGAATTAGTAAAGATGGGGATTTCGATGCCCCCAGATTTTGCTCCAGG
AAAGAGCTGGTCTTATTCAAATACAGGATACGTATTACTTGGTATCCTTATTGAAACAGTAACCGG
GAACAGCTATGCGGAAGAGATTGAAAATCGGATTATTGAACCGCTTGAATTATCGAATACATTCTT
ACCTGGCAATTCAAGCGTGATTCCAGGAACCAAGCATGCCCGTGGATATATCCAACTAGACGGA
GCAAGTGAGCCAAAAGATGTTACTTATTATAACCCAAGTATGGGGAGCTCGGCTGGAGATATGAT
TTCTACTGCTGATGATTTAAACAAATTCTTCTCTTACTTACTTGGGGGTAAATTACTAAAAGAACAG
CAACTAAAACAGATGCTTACTACAGTTCCAACAGGAGAAGCTGCACTTGGCAGATATGGTCTTGG
AATCTATGAAACTAAGCTTCCAAACGGTGTCTCAATATGGGGACACGGAGGTAGCATTCCAGGGT
TTGTTACTTTTGCTGGAGGCACACTTGGAGGCAAGCATACATTAGCTGTCAATTTGAACAGCCTT
AATGCTGAGAGTCCTGATCCTTTTAAAAATATTTTACTTGCTGAATTTAGCAAGGGCAGCAGCCAT
CACCACCATCATCACTAA

Protein sequence

MKTRSQITCASLALLIAGSSLLYTTQTLIVKA(*)EPTQSVSSSVQTSTQRDRNSVKQAVRDTLQLGFPGI
LAKTSEGGKTWSYAAGVANLSSKKPMKTDFRFRIGSVTKTFTATVVLQLAEENRLNLDDSIEKWLPGV
IQGNGYDDKQITIRQLLNHTSGIAEYTRSKSFDLMDTKKSYRAEELVKMGISMPPDFAPGKSWSYSNT
GYVLLGILIETVTGNSYAEEIENRIIEPLELSNTFLPGNSSVIPGTKHARGYIQLDGASEPKDVTYYNPSM
GSSAGDMISTADDLNKFFSYLLGGKLLKEQQLKQMLTTVPTGEAALGRYGLGIYETKLPNGVSIWGH
GGSIPGFVTFAGGTLGGKHTLAVNLNSLNAESPDPFKNILLAEFSKGSSHHHHHH

32 N-terminal residues acids before (*) were previously identified as signal peptide and are cleaved during protein production and purification process.[2]

GFP-M233C

MVSKGEELFTGVVPILVELDGDVNGHKFSVRGEGEGDATNGKLTLKFIS**(C48S)**TTGKLPVPWPTLVT
TLTYGVQM**(C70M)**FSRYPDHMKQHDFFKSAMPEGYVQERTIFFKDDGNYKTRAEVKFEGDTLVNRIE
LKGIDFKEDGNILGHKLEYNYNSHNVYIMADKQKNGIKVNFKIRHNIEDGSVQLADHYQQNTPIGDGPV
LLPDNHYLRTQSALSKDPNEKRDHMVLLEFVTAAGITLG**C(M233C)**DELYKSSHHHHHH

H3K9C

M/ARTKQTAR**C(K9C)**STGGKAPRKQLATKAARKSAPATGGVKKPHRYRPGTVALREIRRYQKSTELLI
RKLPFQRLVREIAQDFKTDLRFQSSAVMALQEAAEAYLVGLFEDTNLAAIHAKRVTIMPKDIQLARRIR
GERA

H3K27C

M/ARTKQTARKSTGGKAPRKQLATKAAR**C(K27C)**SAPATGGVKKPHRYRPGTVALREIRRYQKSTEL
LIRKLPFQRLVREIAQDFKTDLRFQSSAVMALQEAAEAYLVGLFEDTNLAAIHAKRVTIMPKDIQLARRI
RGERA

H3K36C

M/ARTKQTARKSTGGKAPRKQLATKAARKSAPATGGV**C(K36C)**KPHRYRPGTVALREIRRYQKSTEL
LIRKLPFQRLVREIAQDFKTDLRFQSSAVMALQEAAEAYLVGLFEDTNLAAIHAKRVTIMPKDIQLARRI
RGERA

H3K79C

M/ARTKQTARKSTGGKAPRKQLATKAARKSAPATGGVKKPHRYRPGTVALREIRRYQKSTELLIRKLP
FQRLVREIAQDF**C(K79C)**TDLRFQSSAVMALQEAAEAYLVGLFEDTNLAAIHAKRVTIMPKDIQLARRI
RGERA

Trx-SGTA^{C} (residues 213-313)

M/SDKIIHLTDDSFDTDVLKADGAILVDFWAEWCGPCKMIAPILDEIADEYQGKLTVAKLNIDQNPGTAP
KYGIRGIPTLLLFKNGEVAATKVGALSKGQLKEFLDANLAGSGSGHMHHHHHHSSGENLYFQGSTGG
VGSFDIAGLLNNPGFMSMASNLMNNPQIQQLMSGMI**C(S242C)**GGNNPLGTPGTSPSQNDLASLIQA
GQQFAQQMQQQNPELIEQLRSQIRSRTPSASNDDQQE

Ddx4^{N}-F182C (residues 1-236)

GAMGSMGDEDWEAEINPHMSSYVPIFEKDRYSGENGDNFNRTPASSSEMDDGPSRRDHFMKSGFA
SGRNFGNRDAGESNKRDNTSTMGGFGVGKSFGNRGFSNSRFEDGDSSGFWRESSNDSEDNPTRN
RGFSKRGGYRDGNNSEASGPYRRGGRGSFRGSRGGFGLGSPNNDLDPDESMQRTGGL**C(F182C)**
GSRRPVLSGTGNGDTSQSRSGSGSERGGYKGLNEEVITGSGKNSWKSEAEGGES

Supplementary References

[1] Asano Y, *et al.,* An alkaline D-stereospecific endopeptidase with beta-lactamase activity from Bacillus cereus. *The Journal of Biological Chemistry*. **271,** 30256-30262 (1996)

[2] Nakano, S. *et al.,* Structural and computational analysis of peptide recognition mechanism of class-C type penicillin binding protein, alkaline D-peptidase from *Bacillus cereus* DF4-B. *Sci Rep* **5**, 13836 (2015)

[3] Nguyen, U. *et al.,* Accelerated chromatin biochemistry using DNA-barcoded nucleosome libraries. *Nat Methods* **11**, 834–840 (2014).

[4] Arpino JA, *et al.,* Crystal structure of enhanced green fluorescent protein to 1.35 Å resolution reveals alternative conformations for Glu222. PLoS One. **7**, e47132 (2012).

[5] Crabtree M, D. *et al*., Ion binding with charge inversion combined with screening modulates DEAD box helicase phase transitions, Cell Reports. **42(11)** : 2211-1247 (2023).

[6] Wright TH, *et al.,* Posttranslational mutagenesis: A chemical strategy for exploring protein side-chain diversity. *Science* **32**, 173-179 (2016)

[7] Bernardes, G. J. L. *et al.,* Facile conversion of cysteine and alkyl cysteines to dehydroalanine on protein surfaces: Versatile and switchable access to functionalized proteins. *JACS* **130**, 5052-5053 (2008).

[8] Dadová, J. *et al*., Precise Probing of Residue Roles by Post-Translational β,γ-C,N Aza-Michael Mutagenesis in Enzyme Active Sites. *ACS Central Science* **3**, 1168-1173 (2017)

[9] Ge, Y. *et al.,* A Chemical Mutagenesis Approach to Insert Post-translational Modifications in Aggregation-Prone Proteins. ACS Chem Neurosci 13, 1714-1718 (2022)

[10] Shelley R, J. *et al*., Rapid purification of EGFP, EYFP, and ECFP with high yield and purity. *Protein Expression and Purification* **41(1)** : 121-127 (2005)

[11] Morrison PM, J. *et al*., Chemical generation and modification of peptides containing multiple dehydroalanines. *Chem Commun (Camb)*. **51(70)** : 13470-3 (2015).

[12] Castro, J. *et al*., A Convenient Laboratory Preparation of Propargylthiol and Its Derivatives. *Synthesis* **1997**(5): 518-520 (1997)

[13] Pil-Je Um and Dale G. Drueckhammer, Dynamic Enzymatic Resolution of Thioesters. *JACS* **120** (23), 5605-5610 (1998)
